# Supramolecular Complex of Photochromic Diarylethene and Cucurbit[7]uril: Fluorescent Photoswitching System for Biolabeling and Imaging

**DOI:** 10.1021/jacs.2c05036

**Published:** 2022-07-27

**Authors:** Dojin Kim, Ayse Aktalay, Nickels Jensen, Kakishi Uno, Mariano L. Bossi, Vladimir N. Belov, Stefan W. Hell

**Affiliations:** † Department of NanoBiophotonics, Max Planck Institute for Multidisciplinary Sciences (MPI-NAT), 37077 Göttingen, Germany; ‡ Department of Optical Nanoscopy, Max Planck Institute for Medical Research (MPI-MR), 69120 Heidelberg, Germany

## Abstract

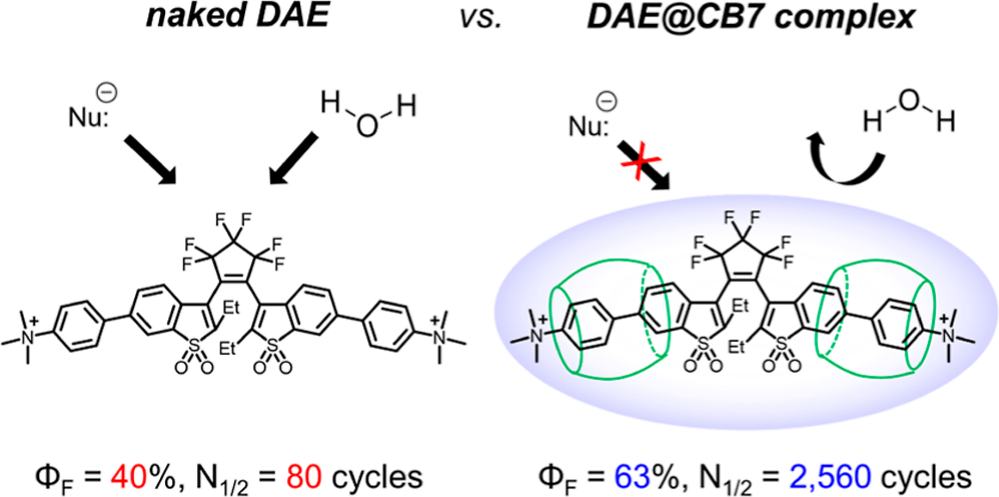

Photoswitchable fluorophores—proteins and synthetic dyes—whose emission is reversibly switched on and off upon illumination, are powerful probes for bioimaging, protein tracking, and super-resolution microscopy. Compared to proteins, synthetic dyes are smaller and brighter, but their photostability and the number of achievable switching cycles in aqueous solutions are lower. Inspired by the robust photoswitching system of natural proteins, we designed a supramolecular system based on a fluorescent diarylethene (**DAE**) and cucurbit[7]uril (CB7) (denoted as **DAE**@CB7). In this assembly, the photoswitchable **DAE** molecule is encapsulated by CB7 according to the host–guest principle, so that **DAE** is protected from the environment and its fluorescence brightness and fatigue resistance in pure water improved. The fluorescence quantum yield (Φ_fl_) increased from 0.40 to 0.63 upon CB7 complexation. The photoswitching of the **DAE**@CB7 complex, upon alternating UV and visible light irradiations, can be repeated 2560 times in aqueous solution before half-bleaching occurs (comparable to fatigue resistance of the reversibly photoswitchable proteins), while free **DAE** can be switched on and off only 80 times. By incorporation of reactive groups [maleimide and *N*-hydroxysuccinimidyl (NHS) ester], we prepared bioconjugates of **DAE**@CB7 with antibodies and demonstrated both specific labeling of intracellular proteins in cells and the reversible on/off switching of the probes in cellular environments under irradiations with 355 nm/485 nm light. The bright emission and robust photoswitching of **DAE-Male3**@CB7 and **DAE-NHS**@CB7 complexes (without exclusion of air oxygen and addition of any stabilizing/antifading reagents) enabled confocal and super-resolution RESOLFT (reversible saturable optical fluorescence transitions) imaging with apparent 70–90 nm optical resolution.

## Introduction

Since the discovery of reversible fluorescence photoswitching in a natural protein (green fluorescent protein, GFP) in 1997,^1^ fluorescence photoswitching has been used in bioimaging, for protein tracking^2,3^ and super-resolution fluorescence microscopy.^4−10^ Repeated fluorescence switching based on reversible photoisomerization of the probes allows to apply them in time-lapse microscopy of living cells and optical nanoscopy based on the principle of reversible saturable optical fluorescence transitions (RESOLFT).^11^ The reversibility of switching in natural proteins relies on the protection of the photochromic unit by the three-dimensional (3D) barrel-shaped polypeptide chain. The barrel provides a noncovalent H-bonding net, in which photoswitchable chromophores (undergoing cis/trans isomerization or photoinduced proton transfer) are surrounded by amino acid residues.^12,13^ Such a kind of natural architecture enhances the fluorescence emission (mostly by suppressing internal conversion pathways)^14^ and dramatically increases the number of switching cycles. Indeed, the surrounding polypeptide acts as a host “container” protecting the chromophore from the environment (nucleophiles, ions, and solvent) and harmful effects of photobleaching (e.g., via photoionization from excited states in water).^15^ Therefore, reversibly photoswitchable fluorescent proteins can have good photochemical/physical properties, such as bright emission, high fatigue resistance, and photostability, and thus operate in aqueous solutions without addition of artificial stabilizers (reducing agents, oxygen scavengers, etc.). The drawbacks of proteins, however, are their relatively large size (∼28 kDa, 4.2 nm) and possible artifacts due to overexpression.^16^

Photoswitching of fluorescence has also been demonstrated by using synthetic probes based on small photochromic molecules, such as azobenzenes, spiropyrans, and diarylethenes (DAEs).^17^ Among them, DAEs, which undergo ring-opening and ring-closure reactions upon alternate irradiations with light of two distinct wavelengths, have been considered as some of the most promising photochromes. They possess thermally stable states, and their switching is reversible and can be repeated multiple hundreds or thousands of times.^18^ The first example of a synthetic photoswitch detectable at a single-molecule level was a dyad composed of a fluorescent dye and a photochromic DAE.^19^ After this seminal work, many dyad-type Förster resonance energy transfer or photo-induced electron transfer photoswitches have been reported.^20−22^ However, the recently developed sulfone DAEs turned out to be superior to the dyad-type photoswitches in terms of the structure simplicity (it unites the switching and fluorescent units), fluorescence turn-on property (i.e., the initially nonfluorescent state converts to a fluorescent one upon irradiation), and high fluorescence quantum yields (up to ∼90%).^23−27^ Simple sulfone DAE cores are hydrophobic and aggregate in water. For applications in biolabeling and imaging, water-soluble polar groups, such as carboxylic or sulfonic acid residues, have been attached to sulfone DAE derivatives.^28−33^ However, simple water-soluble sulfone DAEs without any branched polycarboxylated side chains have low photostability in aqueous solutions, and their switching capacity does not exceed a few tens of cycles (typically 20–80). The low reversibility limits their use as switches in life sciences. For example, the resolution improvement in RESOLFT microscopy is known to be directly proportional to the number of switching cycles.^4,34^ Recently, we reported sulfone DAE with 12 carboxylic acid groups, which exhibited several hundred full switching cycles (up to 1,000) in aqueous solutions.^32^ The high photostability was assumed to be due to the protecting effect of branched linkers terminated with numerous carboxylic acid groups, protecting the core from the solvent and nucleophiles. Though the protective effect against water is important, we also learned that the water-soluble sulfone DAEs drastically lose their switching ability in conjugates with proteins.^28,31−33^ Therefore, new approaches toward protection of the DAE chromophore and increasing its switching performance are highly desirable, though challenging.

Host–guest supramolecular chemistry based on the noncovalent bonding has made tremendous progress^35^ and opened great perspectives in photochemistry fields.^36^ By encapsulating chromophores inside the host–guest complex, many kinds of hydrophobic dyes (e.g., aromatic hydrocarbons) were applied as fluorescent probes in aqueous media and successfully utilized in biological studies.^37^ In addition, complexation could maintain or even improve photophysical properties, such as fluorescence quantum yield,^38,39^ photoisomerization ability,^40^ and photostability.^41−44^ Among various host macrocycles, cucurbit[7]uril (CB7) caught our attention because it exhibits a much higher binding affinity (typically from 10^4^ to 10^15^ M^–1^) than other host molecules, such as cyclodextrins and calixarenes.^36,45,46^ The high affinity of CB7 for guest molecules with a suitable geometry (e.g., ferrocene and adamantane) is similar to binding between natural proteins and their cognate ligands, for example, avidin and biotin (*K*_bind_ ∼ 10^15^ M^–1^).^47^ In addition, CB7 has high water solubility (20–30 mM) and low cytotoxicity (IC_50_ = 0.53 mM),^48^ and its complexes with guest molecules are known to be biocompatible without significant cell damage.^49−51^

**Figure 1:**
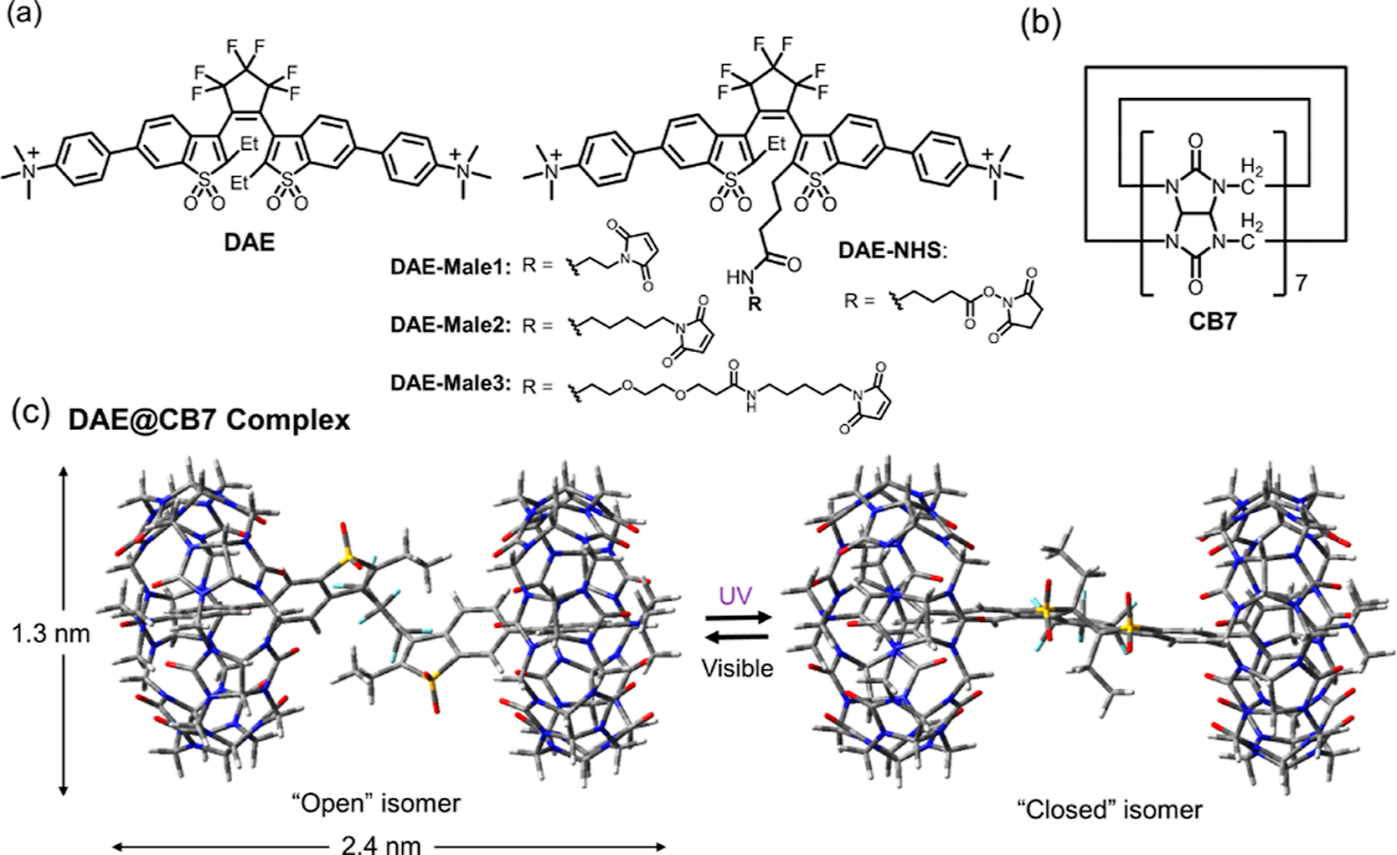
(a) Structures of photochromic diarylethenes as guest molecules (**DAE**, **DAE-Male1**, **DAE-Male2**, **DAE-Male3**, and **DAE-NHS**). (b) Structure of a cucurbit[7]uril host molecule (CB7). (c) DFT-optimized geometry and the photoswitching reaction of the **DAE**@CB7 complex. The cylinder of a **DAE**@CB7 complex has a length of 2.4 nm and a diameter of 1.3 nm.

In the present work, we designed a unique photoswitchable probe for bioimaging, which (1) has a compact size, (2) has a chromophore protected from the environment, (3) provides highly reversible photoswitching with high fluorescence on/off contrast in pure water, and (4) has a reactive group for bioconjugation and imaging of specific subcellular structures in an optical microscope. To this end, we have prepared supramolecular complexes composed of photoswitchable fluorescent sulfone DAE as the guest and CB7 as the host (Figure 1). We incorporated reactive groups [maleimide and *N*-hydroxysuccinimidyl (NHS) ester] into the DAE@CB7 complexes.

## Experimental Section

### Materials

All reagents (incl. CB7) and solvents were purchased from Sigma-Aldrich GmbH, abcr GmbH, and TCI GmbH and used as received. Primary and secondary antibodies were purchased from Abcam PLC (anti-alpha tubulin rabbit) and Jackson ImmunoResearch (AffiniPure Goat anti-Rabbit Polyclonal IgG (H + L)), respectively. Desalting columns (7K MWCO 0.5 ml Zeba Spin) were acquired from Thermo Fisher. Analytical high-performance liquid chromatography (HPLC) was performed with a KNAUER Azura system with a 20 μL injection loop, a 150 × 4 mm column (Knauer, Eurospher II 100-10 C18A), and a photodiode array detector; a flow rate of 1.2 mL/min with water/MeCN gradient (both solvents contained 0.1% of TFA) was used. Flash chromatography was performed on a Biotage Isolera 3.0 flash purification system. Preparative HPLC was performed on a puriFlash 4250 2X preparative HPLC/flash hybrid system (Interchim) with a 2 mL injection loop and a 200–600 nm UV–vis detector. The preparative column was an Interchim Uptisphere Strategy C18-HQ system: 10 μm, 250 × 21.2 mm (US10C18HQ-250/212, Interchim), and flow rate 20 mL/min.

### Nuclear Magnetic Resonance and High-Resolution Mass Spectrometry

Nuclear magnetic resonance (NMR) spectra (^1^H, ^13^C, and ^19^F) were recorded on an Agilent 400MR DD2 (400 MHz for ^1^H) spectrometer. ESI-high-resolution mass spectrometry was performed on a micrOTOF spectrometer (Bruker) equipped with an Apollo ion source and a direct injector with an LC-autosampler Agilent RR 1200 system.

### Photophysical Measurement

Absorption and emission spectra were recorded in a Cary 5000 UV–vis–NIR spectrometer and in a Cary Eclipse fluorescence spectrophotometer, respectively (Agilent Technologies). The fluorescence quantum yields were determined with a Quantaurus-QY absolute photoluminescence (PL) quantum yield spectrometer (model C11347-12, Hamamatsu). Fluorescence lifetimes were recorded and fitted with a Quantaurus-Tau fluorescence lifetime spectrometer (model C11367-32, Hamamatsu). Isomerization quantum yields and fatigue resistance on ensemble (cuvette) experiments were performed on a home-built setup.^52^

### Quantum Chemical Calculation

Semiempirical calculations (PM6) were carried out using the Gaussian 09 quantum chemical package to roughly optimize geometry in vacuum.^53^ Subsequently, geometry optimization of each isomer and host–guest complexes was performed according to the density functional theory (DFT) on B3LYP/6-31G(d,p) in water (CPCM).^54^ Vibration frequency calculations at the same level were performed for the obtained structures to confirm the stable minima.

### Isothermal Titration Calorimetry

The affinity between **DAE** and CB7 was measured by isothermal titration calorimetry (ITC) using a MicroCal PEAQ-ITC instrument (Malvern). All titrations were performed at 25 °C and in pure water. The **DAE** solution (1000 μM) was titrated in 18 injections of 2 μL to CB7 (100 μM) with an injection spacing of 150 s. As the reference run, titration of **DAE** into pure water was performed. The obtained thermogram was baseline-corrected, and the binding signals were corrected for the control measurement. The analysis was conducted using the software supplied by the manufacturer.

### Thiol-Reactive Conjugation of Antibodies

The pH of the antibody solution (0.24 mg in 100 μL) was adjusted to pH ≈ 8 with bicarbonate buffer, and ethylenediaminetetraacetic acid (EDTA) was added to a final concentration of 5 mM. Then, Traut’s reagent [2-iminothiolane in dimethyl formamide (DMF), 10 mM] was added in 40-fold molar excess with respect to the protein, and the solution was incubated for 1 h at room temperature. The samples were overlaid with argon to prevent oxidation of the freshly formed thiols. The excess of Traut’s reagent was removed with a desalting column, equilibrated in 100 mM phosphate buffer adjusted at pH = 8, and supplemented with 5 mM EDTA. The resulting thiolated antibody was immediately mixed with 6-fold molar excess of **DAE-Male** derivatives (from a 5 mM stock solution in DMF) and incubated in the dark at room temperature for 90 min. Unreacted dye was removed using a desalting column equilibrated in PBS.

### Amine-Reactive Conjugation of Antibodies

The pH of the antibody solution (0.24 mg in 100 μL) was adjusted to pH ≈ 8 with a bicarbonate buffer (IM). Then, a 10-fold molar excess of **DAE-NHS** (from a 5 mM stock solution in DMF) was added slowly with stirring, and the mixture was incubated in the dark at room temperature for 1 h. The unreacted dye was removed using a desalting column previously equilibrated with PBS.

### Determination of the Degree of Labeling

UV–vis measurements were used for the determination of the degree of labeling (DOL) or the number of dyes per protein (antibody). The DOL was calculated using the equation given below, where *A*, *c*, and ε correspond to the absorptions, concentrations, and extinction coefficients (M^–1^ cm^–1^), respectively. The absorption of the closed form (CF) at 445 nm was not detectable; therefore it was neglected in the calculations, and it was assumed that all markers were in the open form (OF). The absorption of the OF was detectable at 333 nm, and the extinction coefficient at this wavelength was used to calculate the concentration. This concentration was used to subtract the absorption of the OF from the total absorption at 280 nm to yield the absorption of the antibody at this wavelength, from which the concentration of the antibody was calculated using the extinction coefficient for IgG (210,000 M^–1^ cm^–1^).
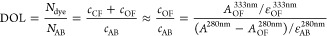

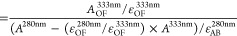


### Cell Culture and Sample Preparation

Cos7 and CV1 cells were grown in full cell culture medium for 12–72 h on glass coverslips and washed twice with PBS (pH 7.4). Then, the cells were fixed with cold methanol (−20 °C) for 5 min and finally washed twice with PBS. To reduce unspecific binding, blocking buffer (2% BSA in PBS) was added, followed by incubation for 30–60 min at room temperature. The coverslips were overlaid with the primary antibody (anti-alpha tubulin) diluted 1:100 in staining buffer (1% BSA in PBS) and incubated in a humid chamber for 60 min at room temperature. Following this, the coverslips were washed with PBS (3 × 5 min). The coverslips were overlaid with the corresponding secondary antibody solution diluted in the staining buffer and incubated in the dark in a humid chamber for 60 min at room temperature. Last, coverslips were washed with PBS (3 × 5 min) and mounted with PBS. When indicated, the medium was exchanged to PBS containing CB7 (2 mM).

### Fluorescence Imaging

RESOLFT and corresponding confocal images were acquired on an Abberior STED microscope (Expert Line, Abberior Instruments). The microscope was equipped with 355 nm (0.5 μW) and 488 nm (13 μW) excitation lines, a 488 nm (28 μW) doughnut-shaped beam off-switching line, and an Olympus UPlanSApo 100×/1.40 oil lens. RESOLFT imaging was performed pixel by pixel (30 × 30 nm pixel size) with a pulse sequence consisting of on-switching with a 355 nm laser (50 μs), followed by off-switching with a doughnut-shaped 488 nm laser (1000 μs), and then excitation with a 488 nm laser (40 μs); detection was set in the range of 506–594 nm. The corresponding confocal image was acquired after the RESOLFT image using the same settings, except for the off-switching step with the doughnut-shaped beam. All images show raw data. Switching experiments were performed by a t-scan in a selected position on the central pixel with the following sequence repeated over 2 s: excitation with the 485 nm laser (500 μs) for probing the off-state, activation with the 355 nm laser (1 ms), excitation by the 485 nm laser (500 μs) for probing the on-state, and finally a longer irradiation step with the 485 nm laser (2 ms) for completion of the off-switching.

## Results and Discussion

### Design and Synthesis

The molecular design of this work is based on encapsulation of a photoswitchable dye as a “guest” into the host–guest supramolecular structure resembling the protective structure of the natural FP (a chromophore located inside a 3D barrel-like structure). A special class of DAEs, which have two oxidized benzothiophene units as the core structure and phenyl rings at C-6,6′ on both sides,^25^ were used as photoswitchable “guest” dyes. These DAEs have high fluorescence quantum yields and a very high on/off fluorescence photoswitching contrast. As the host molecule, CB7 was chosen because it is highly water-soluble and biocompatible and, among macrocyclic hosts, has the highest binding ability toward the aromatics with elongated structures. We expected that the combination of the sulfone DAE and CB7 is a perspective way to achieve highly reversible fluorescence photoswitching in aqueous conditions.

**Scheme 1:**
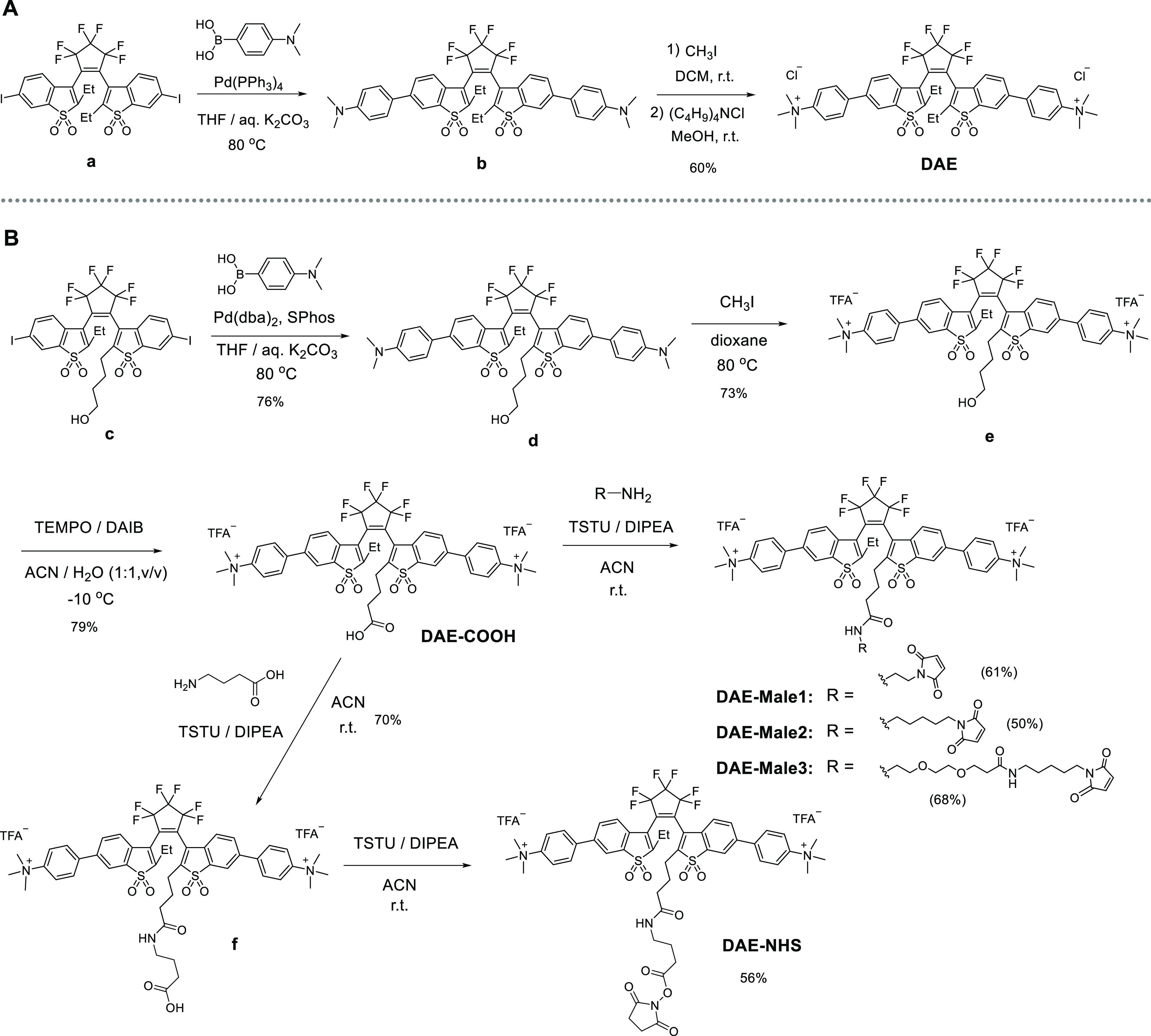
Synthesis of (a) Symmetric **DAE** and (b) Asymmetric **DAEs**: **DAE-COOH**, **DAE-Male1**, **DAE-Male2**, **DAE-Male3**, and **DAE-NHS** For abbreviations, see the Supporting Information.

As shown in Figure 1b, CB7 possesses a hydrophobic cavity and polar urea rims at both sides, thus tending to encapsulate positively charged hydrophobic molecules (e.g., aromatic cations).^55^ To provide affinity to CB7, we synthesized a series of sulfone **DAEs** with positively charged trimethyl ammonium groups at both sides (Figure 1a). First, we prepared the symmetric **DAE**, which was obtained by methylation of the *N*,*N*-dimethyl amino compound **b**,^56^ followed by the counterion exchange (Scheme 1a). This **DAE** contains two charged residues in the structure; it is water-soluble and forms with CB7 the whole assembly **DAE**@CB7. The photoswitching of the amino compound **b** is extremely slow due to a strong intramolecular charge transfer.^56,57^ The switching of **DAE** was expected to be rapid because the transformation of the dimethyl amino group (−NMe_2_) to trimethyl ammonium (−NMe_3_^+^) sharply decreases the electron-donating power of the substituents and reduces the degree of charge transfer between the central sulfone core (acceptor) and the substituents attached to it (also acceptors). The structure of the **DAE**@CB7 (1:2) complex in water was obtained from the DFT calculations (Figure 1c) which show that the chromophore **DAE** is encapsulated inside the complex. The length of the cylinder of the **DAE**@CB7 complex is ∼2.4 nm, and its diameter is ∼1.3 nm. It is noteworthy that the cylindrical volume (calculated by π × *d*^2^ × *L*/4, where *d* and *L* are the diameter and the length of the cylinder, respectively) of **DAE**@CB7 is ca. 6 times smaller than that of the GFP barrel (4.2 nm length × 2.4 nm diameter).^58^

**Figure 2:**
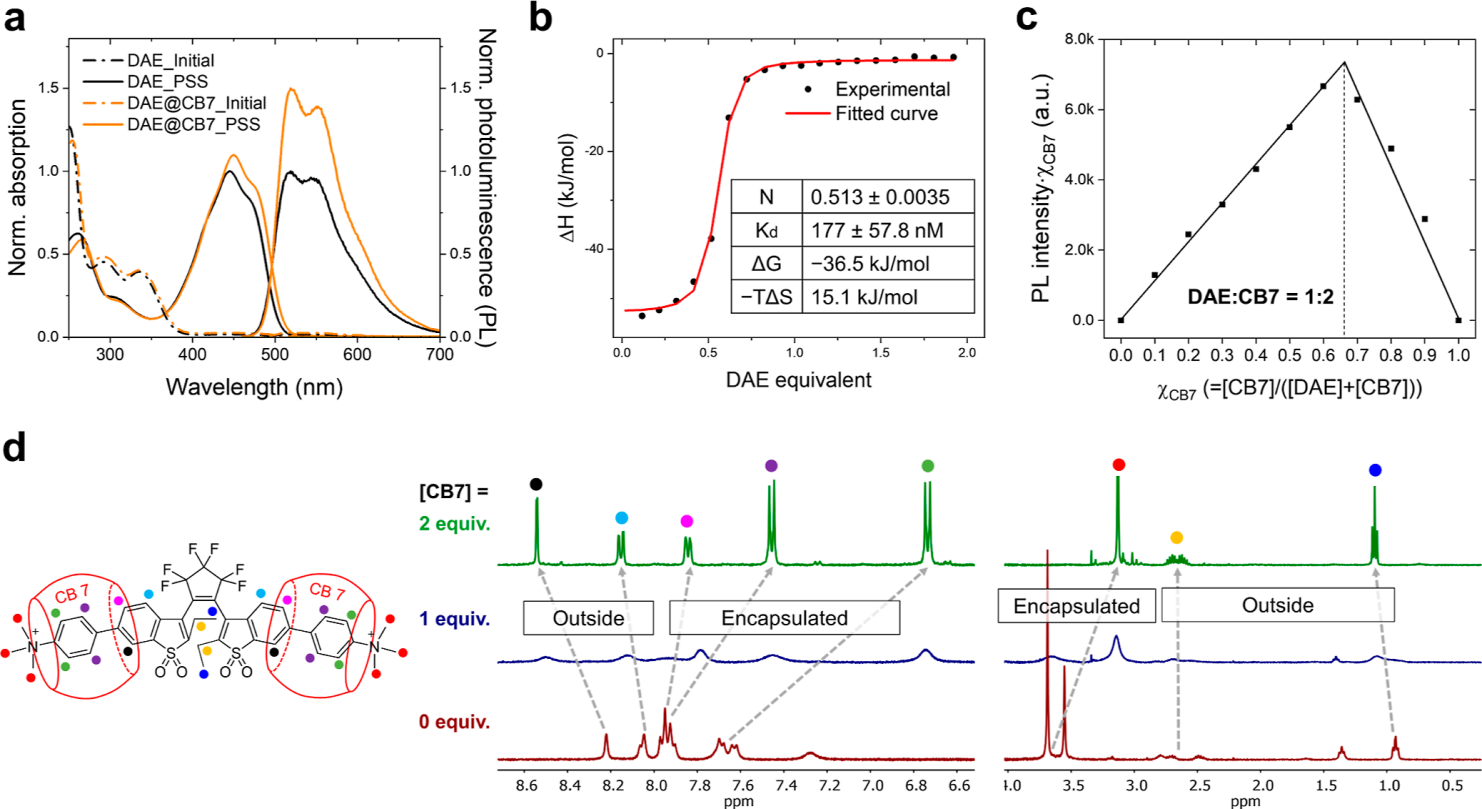
(a) Normalized absorption and PL spectra of **DAE** (black dashed lines for the initial state and black solid lines for the PSS) and **DAE**@CB7 complex (orange dashed lines for the initial state and orange solid lines for the PSS) in aqueous solution (10 μM). Irradiation with UV light to reach PSS states. (b) ITC profiles: integrated heat (experimental) and fitted curve of titrating 1000 μM **DAE** into 100 μM CB7 in water at 25 °C. (c) Job’s plot derived from PL intensities observed for complexation of **DAE** and CB7 ([**DAE**] + [CB7] = 10 μM, χ_CB7_ is the molar fraction of CB7 in a mixture). (d) ^1^H NMR spectra of the open-ring isomer **DAE** upon addition of CB7 in D_2_O ([**DAE**] = 2 mM).

However, the initial nonfunctionalized **DAE**@CB7 complex does not have any functionality in its structure. Therefore, we decided to add a reactive group which can be used for bioconjugation of the **DAE**@CB7 complex. The structure of **DAE**@CB7 was confirmed by DFT calculations and NMR spectroscopy (Figures 1c and 2d). Importantly, ethyl groups at the central carbon atoms (C-2 and C-2′) of **DAE** are located outside of CB7. Indeed, the multiplets of the ethyl groups in the ^1^H NMR spectra were shifted downfield upon complexation. Therefore, we decided to attach the carboxylic acid group to the terminus of the alkyl chain connected with C-2 (Figure 1a). That would make it accessible for the reactions with proteins. Further on, we prepared three maleimide derivatives with various linker lengths (**DAE-Male1**, **DAE-Male2**, and **DAE-Male3** in Figure 1a) because it was observed that the sulfone DAEs may lose their emission properties in “tight” bioconjugates with proteins.^28,31,33^ As the linker length increased, the fluorescent intensity of DAEs was expected to be less influenced by the contact with external biomolecules. In practice, the fluorescence of DAEs is most often increased in the unbound state, or when there is no energy transfer from the excited state of DAE to the π-systems of the amino acids, DNA residues, and other biomolecules.

The synthesis of the functionalized **DAEs** started from compound **c** (Scheme 1b), which has a hydroxyalkyl group at one of the central carbon atoms (C-2/C-2′).^33^ Diiodide **c** and 4-(*N*,*N*-dimethylamino)phenylboronic acid underwent a Suzuki–Miyaura coupling reaction and gave compound **d**. Then, the *N*,*N*-dimethylamino groups were methylated with an excess of iodomethane, and intermediate **e** was prepared. The hydroxyl group of compound **e** was oxidized to the carboxylic acid group by (diacetoxyiodo)benzene in the presence of 2,2,4,4-tetramethyl-1-piperidinyloxyl as a catalyst. The carboxylic acid **DAE-COOH** was amidated with several maleimide derivatives (R–NH_2_ in Scheme 1b) to give **DAE-Male1**, **DAE-Male2**, and **DAE-Male3**. This reaction was “clean,” when we used the peptide coupling reagent *N*,*N*,*N*′,*N*′-tetramethyl-*O*-succinimidyluronium tetrafluoroborate (TSTU) and diisopropylethylamine (DIPEA) as a base. For the preparation of NHS ester, **DAE-COOH** was directly coupled with unprotected γ-aminobutyric acid to give compound **f**. It was isolated, and the addition of TSTU and DIPEA gave NHS ester **DAE-NHS**. The detailed description of the synthesis and the spectra of all compounds (^1^H NMR, ^13^C NMR, and mass) are given in the Supporting Information.

### Complexation between **DAE** and CB7 and the Photophysical Properties of Free **DAE** and **DAE**@CB7 Complex

The photophysical properties of **DAE** and its complex **DAE**@CB7 in an aqueous solution were studied by UV–vis and PL spectroscopies (Figure 2a). At the initial state, **DAE** is in the OF and has the absorption bands in the UV region (λ_max,abs_ = 291 and 333 nm) (see Table 1 and Figure S1). Upon illumination with UV light at 365 nm, the nonfluorescent open-ring isomers of **DAEs** are gradually transformed to the fluorescent closed-ring isomers with absorption and emission in the visible-light region (λ_max,abs_ = 444 nm and λ_max,emi_ = 519, 548 nm). HPLC analysis revealed that the conversion at the photostationary state (PSS) (α_PSS_) is complete (see Table 1). Subsequent visible-light irradiation (λ > 420 nm) induces the reverse (ring-opening) reaction, which recovers the initial absorption bands in the UV-light region, with a decrease in the emission intensity. Thus, the forward and backward photoswitching reactions were found to be fully reversible upon irradiations at two different wavelengths, with both isomers being thermally stable. The cyclization and cycloreversion quantum yields (Φ_OF→CF_ and Φ_CF→OF_) of the free, noncomplexed **DAE** in water were determined as 0.34 and 0.0064, respectively (see Table 1 and Figure S2). Due to the turn-on switching property (i.e., from the nonfluorescent to fluorescent state), the fluorescence on/off contrast is extremely high. The PL quantum yield (Φ_fl_) and the fluorescence lifetime (τ_fl_) of closed-ring DAE were found to be 0.40 and 1.83 ns, respectively (Table 1 and Figure S3a). These values are typical for water-soluble sulfone DAEs in aqueous media.^28,30−33^

**Table 1:** Absorption Maxima (λ_abs_^max^), Extinction Coefficients (ε), Emission Maxima (λ_em_^max^), Fluorescence Quantum Yields (Φ_fl_), Fluorescence Lifetimes (τ_fl_), Isomerization Quantum Yields (Φ_isom_), Conversion Degree at Photostationary State upon 365 nm Irradiation (α_365nm_ = [CF]/*C*_0_) and upon 470 nm Irradiation (α_470nm_ = [OF]/*C*_0_), and Photofatigue Resistance (*N*_1/2_) of Diarylethenes **DAE** and **DAE-COOH** and Their Complexes with CB7 (**DAE**@CB7 and **DAE-COOH**@CB7) Measured in Aqueous Solutions. (OF: Open Form, CF: Closed Form)

	λ_abs_^max^ (nm)/ε × 10^–3^ (M^–1^ cm^–1^)		λ_em_^max^ (nm)	Φ_fl_	τ_fl_ (ns)	Φ_isom_				
	OF	CF	CF	CF	CF	OF → CF	CF → OF	α_365nm_	α_470nm_	*N*_1/2_
**DAE**	291/15.6, 333/13.6	444/27.9	519, 548	0.40	1.83	0.34	6.4 × 10^–3^	1.00	1.00	80
**DAE**@CB7		449/29.3	519, 548	0.63	2.33	0.35	6.9 × 10^–3^			2560
**DAE-COOH**	294/18.7, 327/15.8	446/31.6	519, 550	0.41	1.80	0.29	9.1 × 10^–3^	1.00	1.00	19
**DAE-COOH**@CB7		450/33.9	520, 553	0.62	2.30	0.35	9.2 × 10^–3^			286

The **DAE**@CB7 complex, with its red-shifted and more intense absorption and emission bands, switches similarly to the free **DAE** compound (Figure 2a and Table 1). Thus, the photoswitching of **DAE** not only occurs in the complex with CB7 but is even improved. Interestingly, the enhancement of emission is about 50%, while that of absorption is only about 5%. The resultant PL efficiency (Φ_fl_) of the **DAE**@CB7 complex was determined as 0.63 by using the absolute method (see the Experimental Section); this value is among the highest Φ_fl_ of all fluorescent DAEs dissolved in water. The lifetime of the excited state (τ_fl_) of **DAE**@CB7 was measured to be 2.3 ns (see Figure S3b), which is much longer than that of free **DAE** and corresponds to a ∼2-fold decrease in the nonradiative rate constant upon binding (calculated *k*_nr_ = 3.3 × 10^8^ s^–1^ and 1.6 × 10^8^ s^–1^ for **DAE** and **DAE**@CB7, respectively). To investigate the effect of dissolved oxygen on Φ_fl_ and τ_fl_ of **DAE** and **DAE**@CB7, we measured these values for the aqueous solutions purged with argon gas (see Figure S4 and Table S1). The Φ_fl_ and τ_fl_ values were found to be same in the presence and absence of oxygen.

The complexation and change in the emission signal were monitored by a titration experiment (Figure S5). Before titration, a **DAE** aqueous solution (3 mL, 10 μM) was irradiated with 365 nm light to convert the nonemissive initial state to the emissive PSS state. Then, the PL intensity was monitored by adding small portions (10 μL) of a concentrated CB7 aqueous solution (1 mM) with titration increments of 0.33 equiv. Upon addition of CB7, the PL intensity gradually rose until 2 equiv of CB7 was added (quenching was observed upon adding the first portion of CB7). Further addition (beyond 2 equiv) did not increase the PL intensity any more. In order to determine the value of the binding constant from the titration data, various fitting methods were applied, but none of them provided a reliable value due to the initial quenching observed in the PL spectra and very small changes in the absorption spectra. Instead, ITC measurements were carried out, and the result is shown in Figures 2b and S6. The ITC curve shows that the **DAE**@CB7 complex has a 1:2 stoichiometry (**DAE**/CB7) and is formed in a single-step process (i.e., the two binding sites of **DAE** are indistinguishable). The binding constant was determined to be *K*_a_ = 5.65 × 10^6^ [i.e., the dissociation constant (*K*_**d**_) = 177 nM], which proves quite tight binding and is well within the range of the binding constants reported between positively charged aromatic rings and CB7.^59^ The Job’s plot analysis based on the PL spectra (Figures 2c and S7) showed a maximum emission at a molar fraction of ca. 0.67, and thus it confirmed a 1:2 **DAE**/CB7 stoichiometry for the **DAE**@CB7 complex. The shape of the Job’s plot is a sharp triangle, a strong indication of high binding affinity between two components.^60^ The electrospray ionization mass-spectrum (ESI-MS) of an aqueous solution containing **DAE** and CB7 (Figure S8) suggests the same value (1:2) for the complex **DAE**@CB7. Based on all data, along with DFT calculations, the **DAE**@CB7 is clearly shown to be a 1:2 inclusion complex, and the binding is remarkably strong with enhanced emission of the guest.

To further study the complex formation and the more detailed structure of **DAE**@CB7, we analyzed the ^1^H NMR spectra of **DAE** in D_2_O by varying amounts of CB7 from 0 to 1 and then 2 equiv (see Figures 2d and S9). Initially (without CB7), several proton multiplets overlap because the open-ring isomers of most DAEs exist as mixtures of antiparallel and parallel conformers in solution.^18^ With 1 equiv of CB7, all NMR signals were broadened and blurred, which is due to the equilibrium between free and encapsulated **DAE** with transitions (in the NMR time scale) producing such kind of dynamic effects. With 2 equiv of CB7, the NMR signals become sharp and resolved again, with the new and changed chemical shifts observed upon complete complexation. Most aromatic protons (10 out of a total of 14; highlighted as green, purple, and magenta dots in Figure 2d) and the protons of −NMe_3_^+^ groups (highlighted as red dots in Figure 2d) were shifted upfield (= shielded by outer CB7). These results clearly reveal that the complex of **DAE** (guest) and CB7 (host) is built with two CB7 molecules, and the latter encapsulates a large portion of the guest molecule in the aqueous solution.

Interestingly, with two equiv of CB7, only the signals of an antiparallel conformer were observed. It is indeed a rare case because free DAEs display signals of both conformers (anti-parallel and parallel) in the NMR spectra recorded for solutions.^18^ We performed DFT calculations and optimized the geometry for both antiparallel and parallel conformers of **DAE** with or without CB7. The optimized structures of **DAE**@CB7 (Figure 1c) showed that the trimethylammonium residues and most parts of the aromatic systems are located inside the CB7 cavities, which agrees with the NMR data. As shown in Figure S10, the antiparallel conformer of **DAE**@CB7 is more stable than the parallel conformer (+3.5 kcal/mol vs +8.7 kcal/mol), whereas antiparallel and parallel conformers of free **DAE** have very similar energy levels (+4.2 kcal/mol vs +4.9 kcal/mol, respectively; Figure S11). The antiparallel conformer is photoactive (undergoes the ring-closure), while the parallel conformer is photo-inactive.^18^ We assumed that CB7 complexation would be advantageous for the photocyclization upon UV irradiation. The measured values of the cyclization and cycloreversion quantum yields are given in Table 1 (for details, see Figures S2 and S12–S14). Contrary to the increased amounts of the antiparallel conformer, the cyclization quantum yields of **DAE** derivatives showed only slight increases upon CB7 complexation (from 0.34 to 0.35 for **DAE** and from 0.29 to 0.35 for **DAE-COOH**). The reason may be a result of two opposite effects. A closer observation of the radiative and nonradiative constants (Table S1) suggest that the major fluorescence enhancement comes from a large reduction on the nonradiative constant upon binding. The isomerization rate (*k*_iso_) is probably reduced due to the rigidized environment.^61^ Thus, the increase in the antiparallel conformer may be counteracted by this effect.

**Figure 3:**
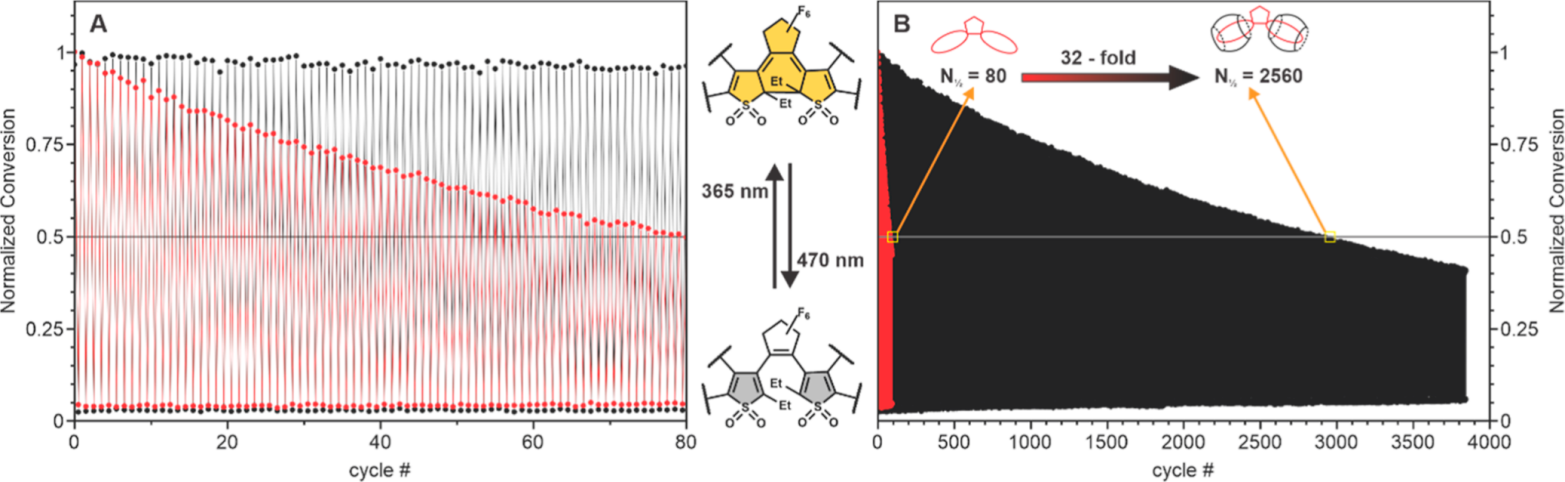
Reversible photoswitching of free **DAE** and **DAE**@CB7 complex in aqueous solutions: (a) zoom into the first 80 cycles; (b) full span of 3800 cycles.

In order to evaluate the photofatigue resistance (N_1/2_; the number of full switching cycles until half of DAE molecules are bleached), the absorptions of free **DAE** and **DAE**@CB7 complex were monitored under repeated irradiations with UV and visible light (Figure 3). Although DAEs are known to have an extremely high photofatigue resistance (e.g., 1.4 × 10^4^ cycles in methylcyclohexane solution^62^ and 3.0 × 10^4^ cycles in the solid state^63^), their switching capacity in aqueous solutions is limited to several tens of switching cycles.^28,30,31,33^ As it can be seen in Figure 3a (see also Table 1), after 80 switching cycles in water, the absorption of the free **DAE** gradually decreases by half. In sharp contrast, the **DAE**@CB7 complex hardly bleaches during the first 80 photoswitching cycles. To evaluate the photofatigue resistance of the complex, the photoswitching experiments were continued up to 3800 cycles (Figure 3b). Strikingly, the monoexponential fit revealed that the *N*_1/2_ value of the **DAE**@CB7 complex in water is 2560 (Table 1), which is 32 times larger than that of free **DAE**. This dramatic enhancement of the photofatigue resistance upon CB7 binding is most likely due to the protective effect of the supramolecular structure which preserves the **DAE** guest from the harmful contacts with water (hydrolysis, oxygen) and photogenerated peroxides. Indeed, HPLC and LC–MS analyses after the repetitive photoswitching experiments showed different byproducts for **DAE** and **DAE**@CB7, respectively (see Figure S15). At the same time, CB7 encapsulation may hinder the unwanted aggregation of **DAE** residues. It should be noted that the binding between **DAE** and CB7 is stable and maintained during the repetitive conformational changes of the guest **DAE** molecule between OF and CF. The stable binding over thousands switching cycles is due to the high binding affinity between the host and guest parts and also because the structural change of the **DAE** framework in the course of the photochromic reaction is very small (see Figure 1c).

### Asymmetric **DAEs** with Reactive Group (**DAE**-Male1,2,3 and **DAE**-NHS) and Their Bioconjugates

Encouraged by the enhanced photophysical properties of **DAE** upon CB7 complexation, we embarked on a synthesis of asymmetric **DAEs** (vide supra, Figure 1a) with a reactive group (maleimide and NHS). Maleimides react with thiol groups (cysteine residues in proteins), and NHS esters react with amines (terminal amino groups of lysine residues in proteins). To characterize the structures of these asymmetric **DAEs** as complexes with CB7, ^1^H NMR titration studies involving **DAE-COOH** and **DAE-Male1** compounds were conducted. As shown in Figures S16 and S17, the carboxylic group of **DAE-COOH** and the maleimide group of **DAE-Male1** are located outside of the complexes with CB7. For example, the olefinic protons of the maleimide **DAE-Male1** undergo downfield shift upon addition of CB7. In contrast to the reactive groups, most of the aromatic signals (10 out of 14 **DAE** protons) and both trimethylammonium groups undergo upfield shifts, like we observed in the ^1^H NMR spectra of symmetric **DAE** (see above). Therefore, the CB7 complexation involves and thus also protects asymmetric **DAE** cores. Fortunately, the reactive groups of the CB7 complexes **DAE-COOH**@CB7 and **DAE-Male1**@CB7 are exposed and remain outside, which enables the reactions with any desired targets, in particular, with proteins.

As shown in Table 1 and Figures S18–S20, the photophysical properties of **DAE-COOH** and **DAE-COOH**@CB7 including absorption/emission spectra, fluorescence quantum yield (Φ_fl_), and fluorescence lifetime (τ_fl_) are similar to those of symmetric **DAE** and **DAE**@CB7. It is not surprising as all compounds have the same chromophore. The binding properties of **DAE-COOH**@CB7, such as the titration curve and the Job’s plot, are similar to those of **DAE**@CB7 complex and indicate the 1:2 stoichiometry. The difference between **DAE** and **DAE-COOH** in the number of switching cycles (*N*_1/2_ = 80 and 19, respectively; see Figures 3 and S21) indicates that asymmetric **DAE-COOH** is more susceptible to photobleaching than the parent compound **DAE**. Upon CB7 complexation, both parameters—the emission intensity and the photofatigue resistance—are improved (Φ_fl_ = 0.41 → 0.62 and *N*_1/2_ = 19 → 286, respectively). Despite the improved value of N_1/2_ for **DAE-COOH**@CB7 observed after complexation, the increase (15 times, *N*_1/2_ = 19 → 286) is lower than that of **DAE**@CB7 (32 times upon complexation, *N*_1/2_ = 80 → 2560). This kind of weaker improvement may be explained if we assume that the linker and the carboxylic acid group of **DAE-COOH** sterically hinder the binding with CB7. Nevertheless, these results clearly confirm that CB7 complexation improves the emission and the number of switching cycles of the guest **DAE** compounds (symmetric and asymmetric). We also conducted UV/PL titration and the Job’s plot experiments for **DAE-Male1**@CB7 (Figures S22 and S23) in order to clarify whether the maleimide group in this probe affects the binding between the host and guest counterparts. As a result, both experiments showed 1:2 host–guest inclusion, which proves that the maleimide group and the linker of **DAE-Male1** do not participate in the complexation with CB7.

To test the performance of our systems in bioconjugates, the four different asymmetric **DAEs** (**DAE-Male1**, **2**, **3** and **DAE-NHS**) with various reactive groups (maleimide and NHS ester) were conjugated with secondary antibodies (for details, see the Experimental Section). The DOL was determined by a standard spectroscopic method (UV–vis; Figure S24). The four bioconjugates have a similar dye/protein ratio (∼3.5–4.5).

### Confocal Bioimaging

**Figure 4:**
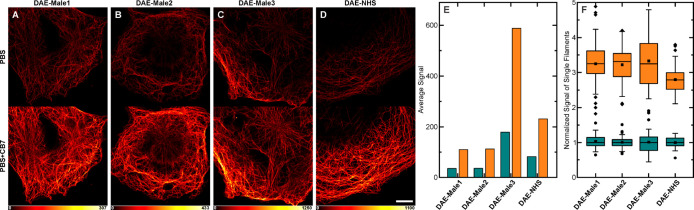
(A–D) Confocal images of fixed Cos7 cells stained with secondary antibodies against microtubules labeled with the indicated **DAE** before (top) and after (bottom) CB7 addition. The markers are switched on before the recording of each pixel, with a short pulse of 355 nm light. (E) Average signal intensity of selected single filaments before (green) and after (orange) CB7 addition. (F) Normalized intensity distribution of selected single filaments (*N* = 50). Scale bar in A–D: 10 μm.

We used indirect immunofluorescence (primary and secondary antibodies) for staining microtubules of fixed cells with bioconjugates (secondary antibodies) decorated with photoswitchable **DAEs** as markers. We labeled mammalian cells and observed them in a confocal fluorescence microscope. The samples were mounted on PBS without any additives. The images were dim, but they all demonstrated high labeling specificity (Figure 4). In order to assess whether the fluorophores can be involved in complexation under these conditions, CB7 was added to the medium at a high concentration of 2 mM. The large excess was necessary (Figure S25) to compete with the high concentration of cations in the buffer used as mounting medium (Na^+^, K^+^). On a timescale of a few seconds, a considerable enhancement of the signal was observed. To quantify this effect, all samples labeled with **DAE-Male1**, **-Male2**, **-Male3**, and **-NHS** bioconjugates were imaged under identical conditions. Five images were acquired before and after the addition of 2 mM CB7 (Figures 4A–D and S26) and then analyzed by measuring the maximum counts along line profiles (8 pixel size) drawn perpendicular to selected single tubulin filaments. For each image, 10 line profiles were measured, resulting in a total of 50 measurements for each sample and condition (in PBS before and after CB7 addition), which were used to calculate the quantitative values presented in Figure 4E,F. On average, ca. 3-fold fluorescence enhancement was observed after the addition of CB7 (Figure 4F). This suggests that the emission of **DAEs** is partially quenched in the bioconjugates compared with the free compounds in water. We hypothesize that two main effects may be responsible for this quenching. First, it may be caused by aggregation of the close-by markers that may not only quench the emission but even prevent on-switching. Possible remedies are based on the incorporation of charged side groups (e.g., sulfonates)^64^ or reducing the DOL values. The first option is already implemented in our **DAEs** having charged trimethylammonium groups, and the second one is inappropriate as it should result in dimmer images or longer recording times. Second, quenching by proteins has been reported for many fluorescent dyes. The quenching effect depends on the antibody and may be even increased upon target binding (i.e., in cells).^65^ For our probes, we also observed a significant dependence of the fluorescence intensity on the nature of the reactive group and/or the linker length. For maleimides, the quenching is reduced by extending the linker (Figure 4E), with the highest effect for **DAE-Male3**. On the whole, complexation with CB7 decreases the quenching effect and improves the emission efficiency.

**Figure 5:**
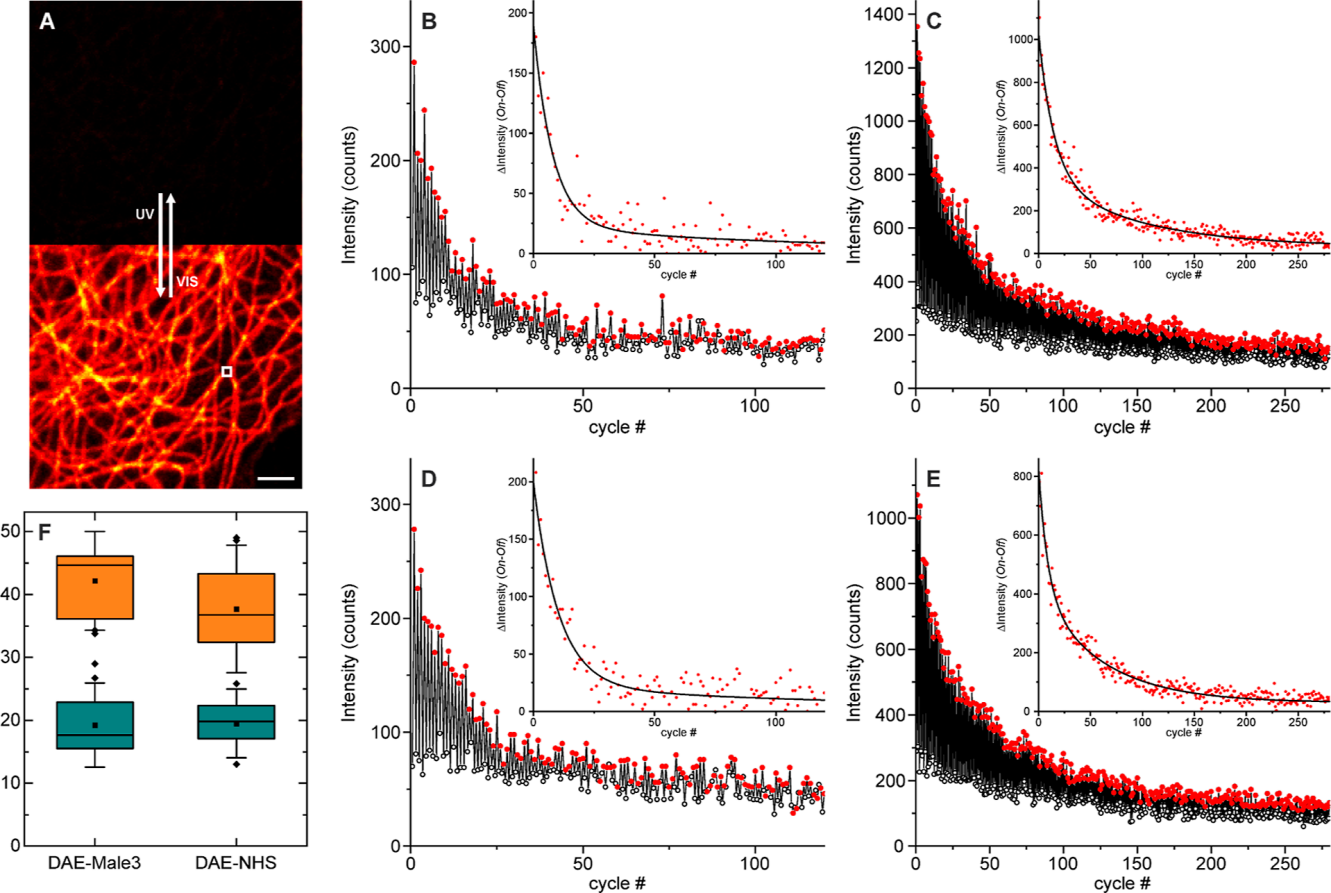
Photoswitching fatigue resistance observed on a confocal microscope for the labeled secondary antibodies on microtubules on fixed Cos7 cells. (A) Confocal image without and with activation (355 nm) after a switching experiment was performed in the indicated area (white square). Switching on and off on a sample labeled with **DAE-Male3** mounted in PBS without (B) and with (C) CB7 (2 mM) and on a sample labeled with **DAE-NHS** without (D) and with (E) CB7 (2 mM). The insets show the differences between two successive substeps (symbols) along with a biexponential fit (lines). (F) Boxplots of the mean (amplitude averaged) characteristic switching time (20 measurements on different positions for each case). Scale bar on A: 2 μm.

Next, we investigated the fatigue resistance on a confocal microscope in PBS buffer (Figure 5) before and after the addition of CB7 (2 mM). The two brightest bioconjugates were selected: **DAE-Male3** and **DAE-NHS**. To this end, we chose a position on a flat area of the cell (Figure 5A) and repeatedly irradiated the sample with 355 and 485 nm light for switching the fluorescence on and off, respectively, while measuring the intensity after each substep (Figure 5B–E). Identical conditions (laser powers and integration times) were used for all measurements. We observed that before the addition of CB7, due to quenching and low signal, the data were quite noisy (Figure 5B,D). In contrast, the signal (brightness) and the switching efficiency are apparently improved after complexation (Figure 5C–E), and the fatigue resistance is increased. For a quantitative assessment of the changes, we calculated the intensity changes (signal_ON_–signal_OFF_) on each switching step (insets in Figure 5B–E) and fitted the transient with a double exponential function, and the amplitude averaged lifetime was calculated. The results from 20 repetitions (different imaged areas) for each compound and mounting media (PBS and 2 mM CB7/PBS) were used to construct the boxplots in Figure 5F. Around a 2-fold increase in fatigue resistance was observed for both bioconjugates under conditions used for confocal imaging. It must be noted that off-switching looks incomplete, which may be due to a fraction of dye that has slow switching or is nonswitchable. The effect appears to be reduced after CB7 binding; thus, it may be due to aggregation or dye–surface interactions that are partially remediated by the complexation.

### Super-resolution RESOLFT Imaging

**Figure 6:**
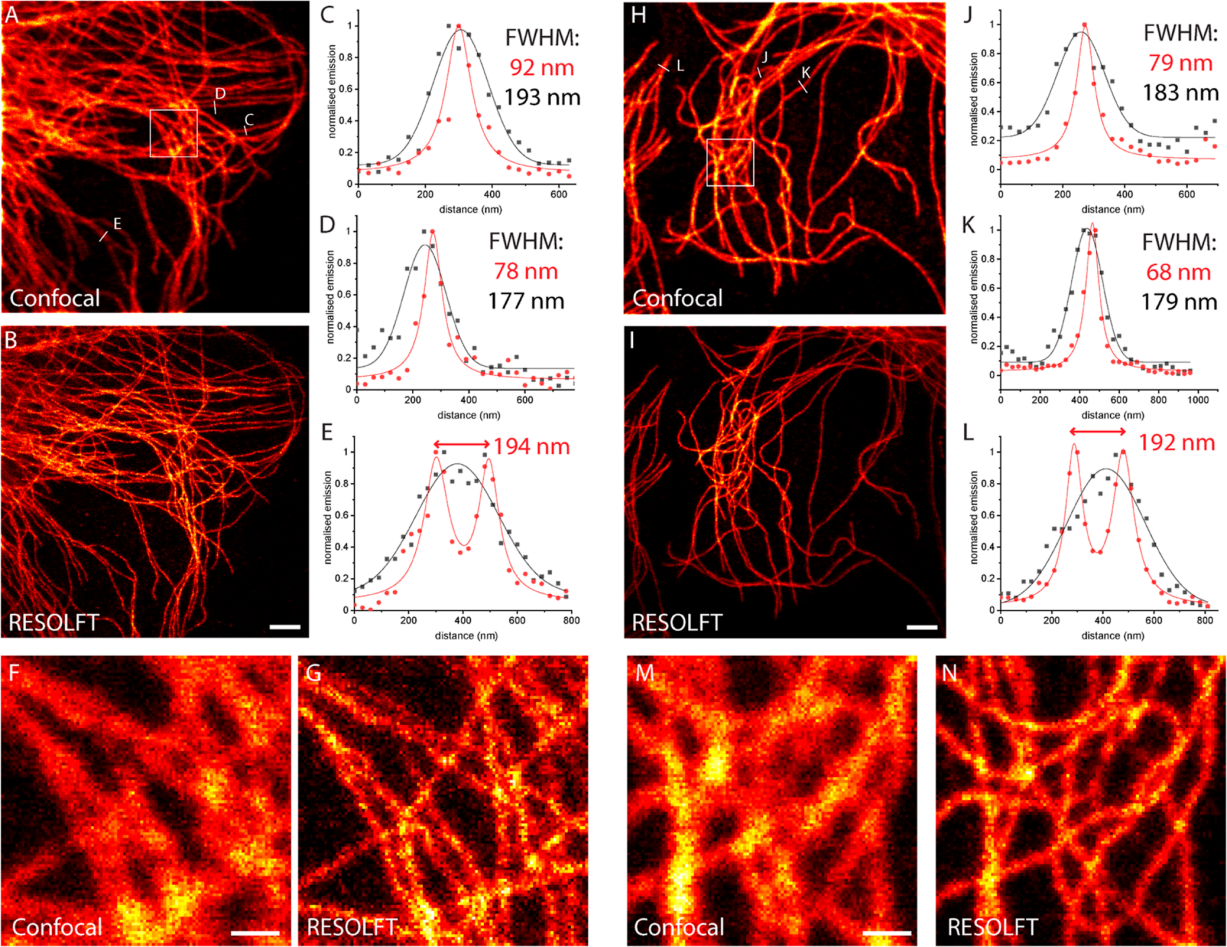
Confocal (A) and RESOLFT (B) images of microtubules on fixed CV1 cells stained with secondary antibodies labeled with **DAE-Male3** and mounted in PBS supplemented with CB7 (2 mM). (C–E) Line profiles along indicated lines show the experimental data (circles) and fitted data (lines). (F,G) Magnifications of the areas indicated in (A). The corresponding results with **DAE-NHS** (with CB7) at the same conditions: confocal (H) and RESOLFT (I) images and (J,K) line profiles. (M,N) Magnifications of the areas indicated in (H). Confocal data (black symbols and lines) were fitted with a Gaussian function and RESOLFT data (red symbols and lines) with a Lorentzian function. Scale bars: 2 μm (A, B, H, I); 0.5 μm (F, G, M, N).

Finally, encouraged by the increased brightness and fatigue resistance after complexation with CB7, we investigated the possibility of increasing the spatial resolution using RESOLFT microscopy. Imaging parameters, on-switching (355 nm), off-switching (488 nm), and excitation (488 nm) dwell times, as well as laser powers, were optimized on the sample mounted with CB7 (2 mM in PBS). RESOLFT imaging in PBS without CB7 resulted in very dim and noisy data due to the low signal and the poor fatigue resistance (Figures S27 and S28 for **DAE-Male3** and **DAE-NHS**, respectively). In the presence of CB7, imaging became possible, and the optical resolution beyond the diffraction limit was achieved (Figure 6A–G and 6H–N for **DAE-Male3**@CB7 and **DAE-NHS**@CB7, respectively). The gain is evident from the normalized line profiles along single filaments (Figure 6C,D,J,K), showing an apparent spatial resolution down to 70–90 nm. Neighboring filaments, unresolved on a confocal image, are clearly separated on the RESOLFT counterpart (Figure 6E,L). For further improvement on the optical resolution, it is mandatory to increase the fatigue resistance under the imaging conditions. Considering the significant differences observed between bulk experiments with mild irradiation intensities (see Figure 3) and cells stained with bioconjugates (Figure 5) under stronger/focused irradiation in a confocal microscope, several improvements can be proposed. In the first place, other branching points for the reactive group should be explored (compare the fatigue resistance between **DAE** and **DAE-COOH**) because this may result in a better protective effect of host molecules. The changes in the length and nature of the linker may also improve the switching fatigue, but the influence of these factors is usually rather difficult to predict as interactions with proteins are extremely complex. Finally, structural variations in the macrocycle (host) and/or DAE (guest) molecules are worth exploring in order to improve the stability of supramolecular binding. For example, a delicate molecular design (small changes) of fluorescent DAE may facilitate much stronger binding with CB7 as it is well known that some guest molecules show exceedingly strong binding affinity to CB7 (up to 10^17^ M^–1^).^47^ In addition, the covalent attachment of the dye (guest) and host molecule is also worth considering.

## Conclusions

We designed and realized a simple and bright photoswitching system based on a supramolecular guest-host principle. The protective cavity for the “guest” turn-on fluorophore (photochromic unit with highly emissive “closed-ring” isomer) is provided by a CB7 host molecule and resembles the protective polypeptide coil of the natural fluorescent proteins. The sulfone-containing **DAEs** having two trimethylammonium residues (−NMe_3_^+^) readily form stable 1:2 complexes with CB7. The complexation enhances the emission efficiency (Φ_fl_; from ∼0.40 to ∼0.63) and the photofatigue resistance. For symmetric **DAE** and asymmetric compound with a reactive group (**DAE-COOH**), we observed a 32-fold and 15-fold increase in the number of switching cycles, respectively. Thus, the switching performance of the fluorescent **DAEs** in an aqueous solution has been improved upon complexation, compared to the previously reported DAEs,^28,31−33,52^ measured in the same setup. In particular, the symmetric compound **DAE**@CB7 displayed 2560 full switching cycles in aqueous solutions until half-bleaching occurred. By incorporating the reactive groups (maleimide or NHS ester) to the complex **DAE**@CB7, specific cell labeling was demonstrated. The longer linker reduces the emission quenching observed upon conjugation with proteins more efficiently than the shorter one and thus induces brighter emission. We observed moderate CB7-induced enhancements in both brightness and fatigue resistance of **DAEs** in cellular environments (3-fold and 2-fold improvements, respectively). Further on, the supramolecular complex of **DAE-Male3**@CB7 and **DAE-NHS**@CB7 was applied in confocal and super-resolution RESOLFT microscopy. The principles of molecular design and the results are expected to provide new insights into the development of robust photoswitchable (supra)molecular systems applicable in aqueous solutions as fluorescent markers for conventional optical microscopy and super-resolution techniques.

## Supporting Information Available

Synthesis of all new compounds with their NMR, MS, and absorption/emission spectra, photoswitching experiments (cyclization QY, cycloreversion QY, and photofatigue resistance), lifetime decay profiles, titration experiments, ITC raw data, Job plots, DFT calculations, data on photofatigue resistance of **DAE-COOH** and **DAE-COOH**@CB7, DOL measurements, and microscopic images (confocal and RESOLFT) (PDF)
