# Supplementary material for: Supramolecular Complex of Photochromic Diarylethene and Cucurbit[7]uril: Fluorescent Photoswitching System for Biolabeling and Imaging
Source: J Am Chem Soc. 2022 Jul 27;144(31):14235–47. doi: 10.1021/jacs.2c05036 (PMC9376957; doi:10.1021/jacs.2c05036)
Supplement: Supplementary file 1 — ja2c05036_si_001.pdf [file ja2c05036_si_001.pdf]

Supporting Information

Supramolecular Complex of Photochromic Diarylethene and Cucurbit[7]uril: Fluorescent Photoswitching System for Biolabeling and Imaging

Dojin Kim,[a,‡] Ayse Aktalay,[b,‡] Nickels Jensen,[a] Kakishi Uno,[a] Mariano L. Bossi,[b,*] Vladimir N. Belov,[a,*] Stefan W. Hell[a,*]

[a] Department of NanoBiophotonics, Max Planck Institute for Multidisciplinary Sciences (MPI-NAT), 37077 Göttingen, Germany

[b] Department of Optical Nanoscopy, Max Planck Institute for Medical Research (MPI-MR), 69120 Heidelberg, Germany

‡ These authors contributed equally.

* Corresponding authors:

mariano.bossi@mr.mpg.de

vladimir.belov@mpinat.mpg.de

stefan.hell@mpinat.mpg.de

Abbreviations

ACN [acetonitrile], ap [antiparallel conformer], Ar [argon], calcd [calculated], CB7 [cucurbit[7]uril], CD_3OD [deuterated methanol], CF [closed form], d [day], DAE [diarylethene], DAIB [diacetoxyiodosobenzene], DCM [dichloromethane], DFT [density functional theory], DIPEA [*N,N*-diisopropyl ethyl amine], DMF [dimethylformamide], EA [ethyl acetate], ESI [electrospray ionization], EtOH [ethanol], equiv. [equivalents], FP [fluorescent protein], GFP [green fluorescent protein], h [hour], HPLC [high performance liquid chromatography], HRMS [high-resolution mass spectrometry], IRF [instrumental response function], K_2CO_3 [photassium carbonate], MeOH [methanol], min [minute], Na_2SO_4 [sodium sulfate], n-Hex [n-hexane], NHS [*N*-hydroxysuccinimidyl], NMR [nuclear magnetic resonance], OF [open form], p [parallel conformer], PBS [phosphate buffered saline], $Pd(dba)_2$ [bis(dibenzylideneacetone)palladium(0)], PL [photoluminescence], RESOLFT [reversible saturable optical fluorescence transitions], RP [reversed phase], rsFP [reversibly photoswitchable fluorescent protein], r.t. [room temperature], SPhos [2-dicyclohexylphosphino-2′,6′-dimethoxybiphenyl], TBAC [tetrabutylammonium chloride], TEMPO [2,2,6,6-tetramethylpiperidine 1-oxyl], TFA [trifluoroacetic acid], THF [tetrahydrofuran], TLC [thin layer chromatography], TSTU [*N,N,N',N'*-tetramethyl-*O*-succinimidyluronium tetrafluoroborate], UV/Vis [ultraviolet/visible light], 3D [three-dimensional].

Synthesis of **DAE**

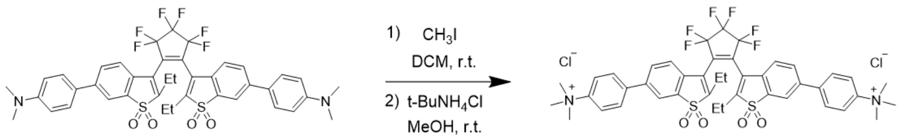

b **DAE**

Compound **b** was prepared according to the literature procedure.[S1] To a DCM solution (15 mL) containing compound **b** (200 mg, 0.25 mmol) was added iodomethane (1.42 g, 10 mmol, 40 equiv.). The reaction mixture was stirred at r.t. for 3 d. The precipitate was filtered and was washed with DCM. The solid product was dissolved in MeOH (10 mL), and a 10 mL MeOH solution containing TBAC (1.39 g, 5.0 mmol, 20 equiv.) was added dropwise. The solution was further stirred for 1 d. The reaction mixture was concentrated in vacuo, and the residue recrystallized from a mixture of EtOH and n-Hex. Yield 135 mg (60%) of **DAE** as an orange-yellow powder. ap:p = 5:3.

^{1}H-NMR (400 MHz, CD_3OD): δ (ppm) = 8.22 (d, J = 1.6 Hz, 1.25H, ap), 8.14 – 8.12 (m, 1.25H, ap), 8.11 (d, J = 1.8 Hz, 0.75H, p), 8.10 – 8.06 (m, 2.75H, ap/p), 8.05 – 7.97 (m, 4.5H, ap/p), 7.95 – 7.89 (m, 1.5H, ap/p), 7.65 (d, J = 8.0 Hz, 0.75H, p), 7.58 (d, J = 8.0 Hz, 1.25H, ap), 3.74 (s, 11.25H, ap), 3.71 (s, 6.75H, p), 2.79 – 2.46 (m, 4H, ap/p), 1.40 (t, J = 7.6 Hz, 2.25H), 1.06 (t, J = 7.6 Hz, 3.75H). ^{13}C NMR (101 MHz, CD_3OD): δ (ppm) = 150.7, 150.5, 148.7, 148.6, 143.1, 143.0, 141.5, 141.4, 138.1, 137.9, 134.3, 134.0, 130.3, 130.2, 123.0, 125.6, 125.4, 124.3, 122.2, 121.9, 121.8, 57.8, 20.4, 20.3, 12.4 12.1. ^{19}F NMR (367 MHz, CD_3OD): δ (ppm) = –111.1 (m, 4F, ap/p), –132.9 (m, 2F, ap/p).

HRMS (ESI+, *m/z*): M^{2+} calcd for $C_{43}H_{42}F_6N_2O_4S_2^{2+}$, 414.1240; found, 414.1250.

Synthesis of compound **d**

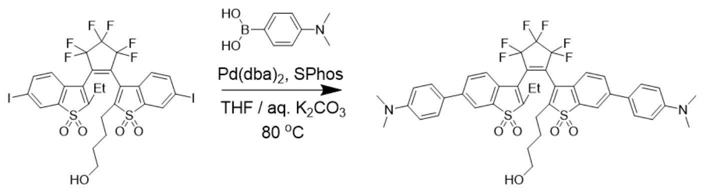

c **d**

Compound **c** was prepared according to the literature procedure.[S2] A flask was charged with compound **c** (150 mg, 0.18 mmol), 4-(dimethylamino)phenylboronic acid (72 mg, 0.44 mmol, 2.5 equiv.), Pd(dba)$_2$ (25 mg, 0.044 mmol, 0.25 equiv.), and SPhos (18 mg, 0.044 mmol, 0.25 equiv.). THF (20 mL) and saturated aqueous K_2CO_3 (20 mL) were added, and the mixture was degassed by Ar bubbling for 10 min. The solution was stirred at 80 °C for 1 h, while monitoring the progress of the reaction by TLC. After the reaction was complete, the mixture was poured into brine and extracted with DCM. The organic solution was dried over Na_2SO_4 and concentrated under reduced pressure. The crude material

was subjected to chromatography on silica gel with gradient elution (EA/n-Hex: 0/100 → 50/50) to give 112 mg (76%) of **d** as an orange powder. ap:p = 5:3.

^{1}H-NMR (400 MHz, DMF-d$_7$): δ (ppm) = 8.29 (dd, *J* = 3.7, 1.6 Hz, 1.25H, ap), 8.17 (d, *J* = 1.7 Hz, 0.75H, p), 8.11 (ddd, *J* = 8.2, 2.8, 1.8 Hz, 1.25H, ap), 7.91 (ddd, *J* = 7.8, 5.9, 1.8 Hz, 0.75H, p), 7.82 – 7.71 (m, 3.25H, ap/p), 7.71 – 7.63 (m, 2.75H, ap/p), 6.90 – 6.84 (m, 2.5H, ap), 6.80 (d, *J* = 8.5 Hz, 1.5H, p), 4.55 (t, *J* = 5.1 Hz, 0.37H, p), 4.47 (t, *J* = 5.1 Hz, 0.63H, ap), 3.59 (q, *J* = 6.2 Hz, 0.75H, p), 3.38 (ddt, *J* = 21.6, 10.9, 5.4 Hz, 1.25H, ap), 3.03 (s, 7.5H, ap), 2.98 (s, 4.5H, p), 2.81 – 2.47 (m, 4H, ap/p), 2.06 – 1.34 (m, 5.12H, ap/p), 1.00 (t, *J* = 7.6 Hz, 1.88H, p). ^{13}C NMR (101 MHz, DMF-d$_7$): δ (ppm) = 152.34, 152.31, 152.25, 148.7, 148.4, 147.8, 147.6, 145.4, 145.2, 145.1, 137.6, 137.50, 137.47, 131.9, 131.8, 131.41, 131.38, 128.94, 128.89, 128.84, 127.0, 126.9, 126.8, 126.7, 125.7, 125.64, 125.55, 125.50, 124.7, 124.4, 120.03, 119.97, 119.89, 113.59, 113.57, 113.51, 62.0, 61.9, 40.60, 40.56, 33.8, 33.7 26.6, 26.6, 25.5, 25.3, 20.1, 19.8, 12.9, 12.5. ^{19}F NMR (367 MHz, DMF-d$_7$): δ (ppm) = –109.8 (m, 4F, ap/p), –131.2 (m, 2F, ap/p).

HRMS (ESI+, *m/z*): $[M+2H]^{2+}$ calcd for $C_{43}H_{42}F_6N_2O_5S_2^{2+}$, 422.1214; found, 422.1215.

Synthesis of compound **e**

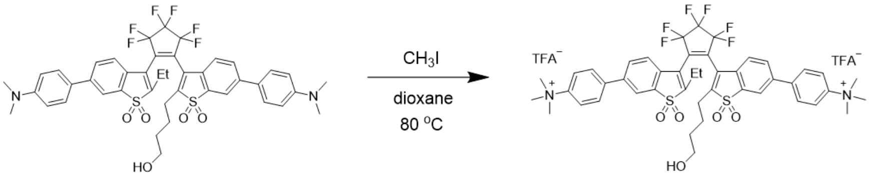

d **e**

To a dioxane solution (2 mL) containing compound **d** (54 mg, 64 μmol), was added iodomethane (2 mL, 32 mmol, 500 equiv.). The mixture was heated to 80 °C and stirred for 24 h. The precipitate was filtered off and subjected to preparative HPLC with gradient elution (0.1% aq. TFA / ACN, 85/15 → 10/90). Lyophilization yielded 52 mg (73%) of compound **e** as a yellow powder. ap:p = 7:3.

^{1}H-NMR (400 MHz, DMF-d$_7$): δ (ppm) = 8.57 (dd, *J* = 5.9, 1.6 Hz, 1.4H, ap), 8.47 (d, *J* = 1.6 Hz, 0.6H, p), 8.39 – 8.32 (m, 4H, ap/p), 8.30 – 8.21 (m, 4H, ap/p), 8.18 (dq, *J* = 6.3, 2.1 Hz, 0.6H, p), 8.16 – 8.11 (m, 1.4H, ap), 8.01 – 7.97 (m, 0.6H, p), 7.90 (t, *J* = 8.5 Hz, 1.4H, ap), 3.96 (s, 12.6H, ap), 3.91 (s, 5.4H, p), 3.61 (t, *J* = 6.2 Hz, 0.6H, p), 3.41 (t, *J* = 6.2 Hz, 1.4H, ap), 2.89 – 2.54 (m, 4H, ap/p), 2.08 – 1.34 (m, 4.9H, ap/p), 1.03 (t, *J* = 7.6 Hz, 2.1H, ap). ^{13}C NMR (101 MHz, DMF-d$_7$): δ (ppm) = 159.8, 159.5, 150.3, 149.5, 149.12, 149.09, 149.05, 142.8, 142.7, 142.5, 140.4, 140.3, 140.2, 137.6, 137.5, 137.4, 134.60, 134.58, 134.2, 134.1, 130.0, 129.94, 129.87, 129.8, 129.7, 129.6, 126.0, 125.94, 125.85, 124.3, 124.0, 122.68, 122.65, 122.59, 122.12, 122.08, 122.0, 120.0, 117.1, 62.0, 61.9, 57.74, 57.69, 33.7, 33.6, 26.7, 26.6, 25.4, 25.2, 20.3, 20.0, 12.8, 12.4. ^{19}F NMR (367 MHz, DMF-d$_7$): δ (ppm) = –109.5 (m, 4F,

ap/p), –130.7 (m, 2F, ap/p). HRMS (ESI+, *m/z*): M^{2+} calcd for $C_{45}H_{46}F_6N_2O_5S_2^{2+}$, 436.1371; found, 436.1372.

Synthesis of compound **DAE-COOH**

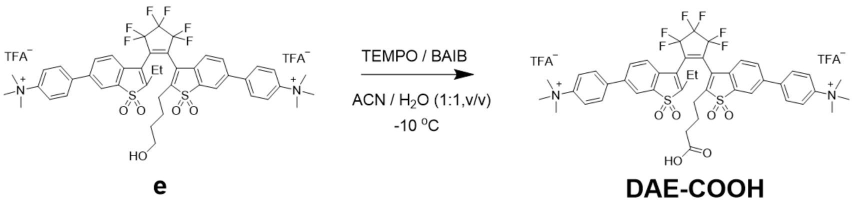

Compound **e** (30 mg, 27 μmol) was dissolved in a mixture of ACN (400 μL) and water (400 μL), and the solution was cooled to –10 °C. To this solution were added TEMPO (21 mg, 140 μmol, 5 equiv.) and DAIB (88 mg, 270 μmol, 10 equiv.), and the reaction mixture was stirred for 2 h, while monitoring the progress of the reaction by analytical HPLC. Purification was carried out by preparative HPLC with gradient elution (0.1% aq. TFA / ACN, 95/5 → 50/50). Lyophilization yielded 24 mg (79%) of compound **DAE-COOH** as a yellow powder. ap:p = 7:3.

^{1}H-NMR (400 MHz, DMF-d$_7$): δ (ppm) = 8.57 (dd, *J* = 18.2, 1.6 Hz, 1.4H, ap), 8.48 – 8.40 (m, 0.6H, p), 8.38 – 8.12 (m, 10H, ap/p), 7.98 – 7.89 (m, 2H, ap/p), 3.96 (d, *J* = 1.6 Hz, 12.6H, ap), 3.91 (d, *J* = 2.4 Hz, 5.4H, p), 2.88 – 2.55 (m, 4H, ap/p), 2.50 – 1.61 (m, 4H, ap/p), 1.42 (t, *J* = 7.5 Hz, 0.9H, p), 1.01 (t, *J* = 7.5 Hz, 2.1H, ap). ^{13}C NMR (101 MHz, DMF-d$_7$): δ (ppm) = 174.83, 174.77, 149.13, 149.06, 142.9, 142.8, 140.5, 140.3, 137.5, 134.6, 134.5, 130.02, 129.97, 129.90, 129.86, 129.7, 129.6, 126.1, 125.9, 124.8, 124.7, 123.98, 123.97, 122.74, 122.68, 122.62, 122.59, 122.1, 57.75, 57.70, 31.2, 25.75, 25.70, 23.96, 23.90, 19.99, 19.96, 12.8, 12.2. ^{19}F NMR (367 MHz, DMF-d$_7$): δ (ppm) = –109.1 (m, 4F, ap/p), –130.5 (m, 2F, ap/p). HRMS (ESI+, *m/z*): M^{2+} calcd for $C_{45}H_{44}F_6N_2O_6S_2^{2+}$, 443.1267; found, 443.1266.

Synthesis of compound **DAE-Male1**

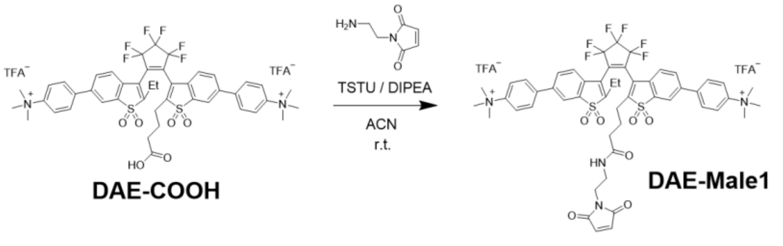

To an ACN solution (1.5 mL) containing compound **DAE-COOH** (32 mg, 28 μmol) and TSTU (17 mg, 57 μmol, 2 equiv.), was added DIPEA (15 mg, 114 μmol, 4 equiv.). The reaction mixture was stirred at r.t. for 10 min. 1-(2-Aminoethyl)maleimide hydrochloride (20 mg, 114 μmol, 4 equiv.) and DIPEA (15 mg, 114 μmol, 4 equiv.) were added to the reaction mixture which was then stirred for 30 min at r.t.

Purification was carried out by preparative HPLC with gradient elution (0.1% aq. TFA / ACN, 95/5 → 50/50). Lyophilization yielded 22 mg (61%) of compound **DAE-Male1** as a yellow powder. ap:p = 7:3. ^{1}H-NMR (400 MHz, DMF-d$_7$): δ (ppm) = 8.55 (dd, *J* = 14.4, 1.6 Hz, 1.4H, ap), 8.46 (t, *J* = 1.7 Hz, 0.6H, p), 8.38 – 8.31 (m, 4H, ap/p), 8.31 – 8.22 (m, 4H, ap/p), 8.21– 7.86 (m, 4H, ap/p), 7.02 (s, 0.6H, p), 7.00 (s, 1.4H, ap), 3.96 (s, 12.6H, ap), 3.91 (d, *J* = 0.8 Hz, 5.4H, p), 3.66 – 3.22 (m, 4H, ap/p), 2.91 – 2.59 (m, 4H, ap/p), 2.42 – 1.59 (m, 4H, ap/p), 1.42 (t, *J* = 7.5 Hz, 0.9H), 1.07 (t, *J* = 7.6 Hz, 2.1H). ^{13}C NMR (101 MHz, DMF-d$_7$): δ (ppm) = 172.8, 172.7, 172.32, 172.28, 149.11, 149.08, 149.06, 142.8, 142.7, 142.5, 140.5, 140.3, 140.2, 137.6, 137.5, 137.4, 135.6, 134.7, 134.6, 130.07, 129.95, 129.88, 129.86, 129.75, 129.6, 125.9, 125.8, 124.5, 124.0, 122.7, 122.6, 122.1, 57.8, 26.2, 24.14, 24.11, 20.3, 20.1, 12.8, 12.4. ^{19}F NMR (367 MHz, DMF-d$_7$): δ (ppm) = –109.2 (m, 4F, ap/p), –130.4 (m, 2F, ap/p). HRMS (ESI+, *m/z*): M^{2+} calcd for $C_{51}H_{50}F_6N_4O_7S_2^{2+}$, 504.1507; found, 504.1520.

Synthesis of compound **DAE-Male2**

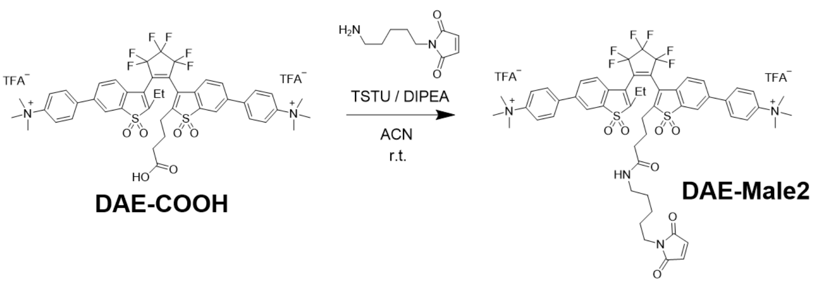

To an ACN solution (1 mL) containing compound **DAE-COOH** (25 mg, 22 μmol) and TSTU (14 mg, 45 μmol, 2 equiv.), was added DIPEA (12 mg, 90 μmol, 4 equiv.). The reaction mixture was stirred at r.t. for 10 min. N-(5-Aminopenthyl)maleimide hydrochloride (20 mg, 90 μmol, 4 equiv.) and DIPEA (12 mg, 90 μmol, 4 equiv.) were added to the reaction mixture, which was then stirred for 40 min at r.t. Purification was carried out by preparative HPLC with gradient elution (0.1% aq. TFA / ACN, 95/5 → 50/50). Lyophilization yielded 14.4 mg (50%) of compound **DAE-Male2** as a yellow powder. ap:p = 7:3.

^{1}H-NMR (400 MHz, DMF-d$_7$): δ (ppm) = 8.56 (dd, *J* = 14.7, 1.7 Hz, 1.4H, ap), 8.47 (d, *J* = 1.7 Hz, 0.6H, p), 8.38 – 8.31 (m, 4H, ap/p), 8.31 – 8.22 (m, 4H, ap/p), 8.22 – 7.87 (m, 4H), 7.03 (s, 0.6H, p), 7.02 (s, 1.4H, ap), 3.96 (d, *J* = 1.3 Hz, 12.6H, ap), 3.91 (d, *J* = 2.1 Hz, 5.4H, p), 3.53 – 3.03 (m, 4H, ap/p), 2.90 – 2.56 (m, 4H, ap/p), 2.46 – 1.22 (m, 10.9H, ap/p), 1.05 (t, *J* = 7.6 Hz, 2.1H, p). ^{13}C NMR (101 MHz, DMF-d$_7$): δ (ppm) = 172.30, 172.28, 172.2, 159.9, 159.5, 150.3, 149.11, 149.08, 149.05, 142.8, 142.7, 142.54, 142.48, 140.5, 140.3, 140.23, 140.20, 137.54, 137.48, 137.46, 137.4, 135.6, 134.7, 134.59, 130.04, 129.95, 129.88, 129.85, 129.75, 129.6, 126.0, 125.8, 124.6, 122.7, 122.6, 122.1, 122.03, 121.99, 57.8, 57.74, 57.69, 39.80, 39.77, 38.32, 38.31, 26.2, 26.1, 25.1, 25.0, 24.7, 24.5, 20.0, 12.8, 12.4. ^{19}F NMR (367 MHz, DMF-d$_7$): δ (ppm) = –109.2 (m, 4F, ap/p), –130.5 (m, 2F, ap/p).

HRMS (ESI+, *m/z*): M^{2+} calcd for $C_{54}H_{56}F_6N_4O_7S_2^{2+}$, 525.1742; found, 525.1754.

Synthesis of compound **SI-a**

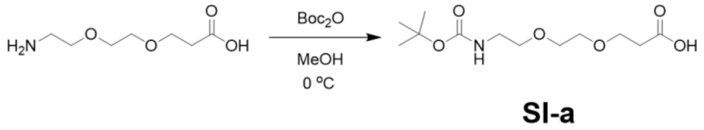

NH2-PEG2-COOH (100 mg, 0.56 mmol) was dissolved in 3 mL anhydrous MeOH, and the solution was stirred at 0 °C for 5 min. Di-tert-butyl dicarbonate (123 mg, 0.56 mmol, 1 equiv.) was added to the reaction mixture, which was then stirred for 30 min at 0 °C. The temperature was changed to r.t., and then the mixture was stirred for 90 min. The mixture was poured into DCM and brine, and extracted with DCM. The combined organic solutions were dried over Na_2SO_4 and evaporated. Lyophilization with 1,4-dioxane yielded 112 mg (72%) of compound **SI-a** as a pale yellow oil.
^{1}H-NMR (400 MHz, chloroform-*d*): δ (ppm) = 5.07 (s, 1H), 3.77 (t, *J* = 6.1 Hz, 2H), 3.65 – 3.56 (m, 4H), 3.56 – 3.47 (m, 2H), 3.34 – 3.21 (m, 2H), 2.79 – 2.52 (m, 2H), 1.44 (s, 9H).
HRMS (ESI+, *m/z*): $[M+Na]^+$ calcd for $C_{12}H_{23}NNaO_6^+$, 300.1418; found, 300.1420.

Synthesis of compound **SI-b**

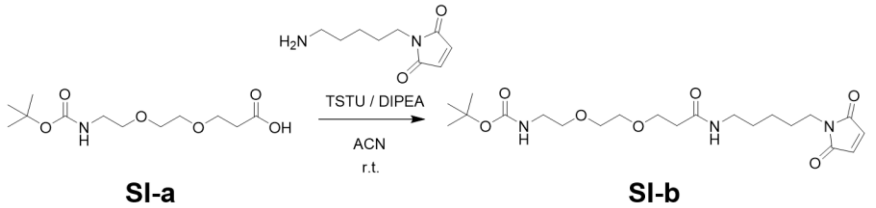

To a DMF solution (1.5 mL) containing compound **SI-a** (25 mg, 90 μmol) and TSTU (41 mg, 140 μmol, 1.5 equiv.), was added DIPEA (41 mg, 320 μmol, 3.5 equiv.). The reaction mixture was stirred at r.t. for 10 min. N-(5-Aminopenthyl)maleimide hydrochloride (59 mg, 270 μmol, 3 equiv.) and DIPEA (12 mg, 320 μmol, 3.5 equiv.) were added to the reaction mixture, which was then stirred for 30 min at r.t. The crude material was subjected to chromatography on silica gel with gradient elution (MeOH/DCM: 0/100 → 15/85) to give 36 mg (90%) of **SI-b** as a yellow oil.
^{1}H-NMR (400 MHz, Chloroform-*d*): δ (ppm) = 6.68 (s, 2H), 6.30 (s, 1H), 5.01 (s, 1H), 3.73 (t, *J* = 5.8 Hz, 2H), 3.62 (s, 4H), 3.57 – 3.48 (m, 4H), 3.35 – 3.26 (m, 2H), 3.22 (q, *J* = 6.9 Hz, 2H), 2.46 (t, *J* = 5.8 Hz, 2H), 1.64 – 1.48 (m, 4H), 1.44 (s, 9H), 1.35 – 1.28 (m, 2H).
HRMS (ESI+, *m/z*): $[M+Na]^+$ calcd for $C_{21}H_{35}N_3NaO_7^+$, 464.2367; found, 464.2364.

Synthesis of compound **SI-c**

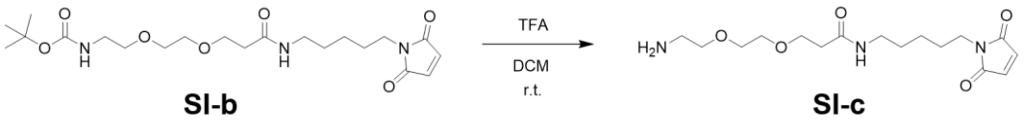

To a DCM solution (1.2 mL) containing compound **SI-b** (30 mg), was added TFA (300 μL). The reaction mixture was stirred at r.t. for 3 h. Evaporation and lyophilization with 1,4-dioxane yielded 30 mg (99%) of compound **SI-c** as a pale yellow oil.

^{1}H-NMR (400 MHz, Chloroform-*d*): δ (ppm) = 8.05 (s, 2H), 6.70 (s, 2H), 6.45 (s, 1H), 3.83 – 3.75 (m, 4H), 3.70 – 3.61 (m, 4H), 3.54 – 3.49 (m, 2H), 3.27 – 3.18 (m, 4H), 2.47 (t, *J* = 5.5 Hz, 2H), 1.65 – 1.49 (m, 4H), 1.34 – 1.28 (m, 2H).

HRMS (ESI+, *m/z*): $[M+Na]^+$ calcd for $C_{21}H_{35}N_3NaO_7^+$, 464.2367; found, 464.2364.

Synthesis of compound **DAE-Male3**

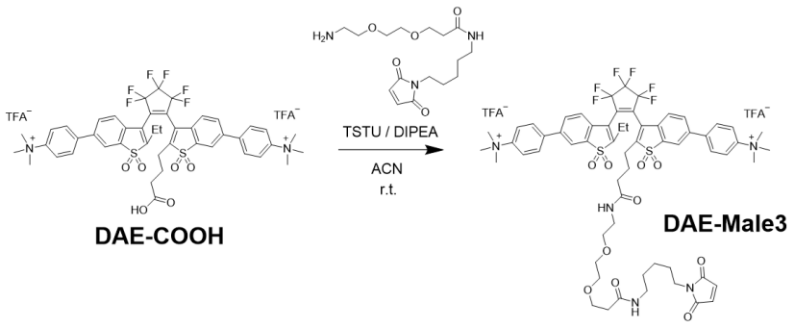

To a DMF solution (600 μL) containing compound **DAE-COOH** (10 mg, 9.0 μmol) and TSTU (5.4 mg, 18 μmol, 2 equiv.), was added DIPEA (9.3 mg, 36 μmol, 4 equiv.). The reaction mixture was stirred at r.t. for 10 min. Compound **SI-c** (9.2 mg, 27 μmol, 3 equiv.) and DIPEA (9.3 mg, 36 μmol, 4 equiv.) were added to the reaction mixture, which was then stirred for 30 min at r.t. Purification was carried out by preparative HPLC with gradient elution (0.1% aq. TFA / ACN, 95/5 → 70/30). Lyophilization yielded 8.8 mg (68%) of compound **DAE-Male3** as an orange yellow powder. ap:p = 7:3.

^{1}H-NMR (400 MHz, DMF-d_7): δ (ppm) = 8.56 (dd, *J* = 13.5, 1.7 Hz, 1.4H, ap), 8.46 (d, *J* = 1.6 Hz, 0.6H, p), 8.37 – 8.32 (m, 4H, ap/p), 8.30 – 8.23 (m, 4H, ap/p), 8.22 – 7.79 (m, 4H, ap/p), 7.03 – 6.99 (m, 2H, ap/p), 3.96 (s, 12.6H, ap), 3.91 (d, *J* = 2.3 Hz, 5.4H, p), 3.73 – 3.07 (m, 14H, ap/p), 2.90 – 2.55 (m, 4H, ap/p), 2.47 – 1.23 (m, 12.9H, ap/p), 1.04 (t, *J* = 7.6 Hz, 2.1H, ap). ^{13}C NMR (101 MHz, DMF-d_7): δ (ppm) = 172.5, 172.3, 171.33, 171.29, 149.10, 149.07, 149.04, 142.8, 142.7, 142.53, 142.47, 140.5, 140.3, 140.22, 140.19, 137.54, 137.48, 137.4, 135.6, 134.7, 134.6, 130.1, 130.0, 129.9, 129.8, 129.7, 129.6, 126.2, 125.9, 124.5, 123.9, 123.8, 122.7, 122.59, 122.57, 122.1, 122.0, 71.1, 71.0, 71.0, 70.9, 70.7, 70.5, 68.33, 68.30, 57.8, 57.74, 57.69, 40.0, 39.9, 39.7, 38.3, 37.62, 37.55, 29.1, 26.1, 25.0, 24.4, 20.0, 12.8, 12.4. ^{19}F NMR (367 MHz, DMF-d_7): δ (ppm) = –109.1 (m, 4F, ap/p), –130.3 (m, 2F, ap/p).

HRMS (ESI+, *m/z*): M^{2+} calcd for $C_{61}H_{69}F_6N_5O_{10}S_2^{2+}$, 604.7190; found, 604.7173.

Synthesis of compound **f**

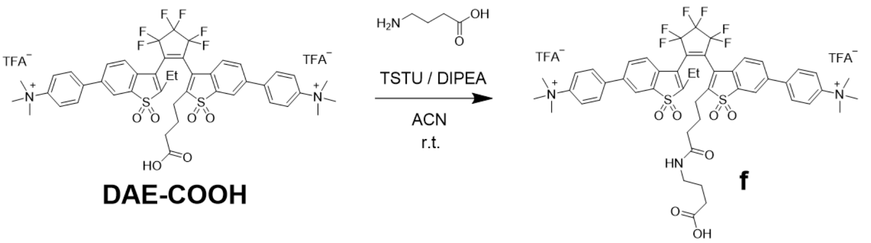

To an ACN solution (900 μL) containing compound **DAE-COOH** (25 mg, 22 μmol) and TSTU (14 mg, 45 μmol, 2 equiv.), was added DIPEA (12 mg, 90 μmol, 4 equiv.). The reaction mixture was stirred at r.t. for 10 min. γ-Aminobutyric acid (9.3 mg, 90 μmol, 4 equiv.) and DIPEA (12 mg, 90 μmol, 4 equiv.) were added to the reaction mixture which was then stirred for 40 min at r.t. Purification was carried out by preparative HPLC with gradient elution (0.1% aq. TFA / ACN, 95/5 → 50/50). Lyophilization yielded 18.7 mg (70%) of compound **f** as a yellow powder. ap:p = 7:3.

^{1}H-NMR (400 MHz, DMF-d$_7$): δ (ppm) = 8.55 (dd, *J* = 15.4, 1.6 Hz, 1.4H, ap), 8.47 (s, 0.6H, p), 8.38 – 8.31 (m, 4H, ap/p), 8.31 – 8.22 (m, 4H, ap/p), 8.22 – 7.86 (m, 4H, ap/p), 3.96 (d, *J* = 1.5 Hz, 12.6H, ap), 3.91 (d, *J* = 1.8 Hz, 5.4H, p), 3.28 – 3.08 (m, 2H, ap/p), 2.90 – 2.58 (m, 4H, ap/p), 2.48 – 1.62 (m, 8H, ap/p), 1.43 (t, *J* = 7.5 Hz, 0.9H, p), 1.05 (t, *J* = 7.5 Hz, 2.1H). ^{13}C NMR (101 MHz, DMF-d$_7$): δ (ppm) = 175.3, 175.2, 172.4, 159.9, 159.6, 149.1, 149.0, 142.8, 142.7, 142.53, 142.47, 140.4, 140.3, 140.21, 140.19, 137.54, 137.46, 137.45, 137.4, 134.7, 134.6, 134.2, 134.1, 130.0, 129.94, 129.87, 129.8, 129.7, 129.64, 129.58, 126.0, 125.8, 124.5, 123.9, 122.7, 122.60, 122.57, 122.5, 122.1, 122.0, 57.73, 57.68, 39.4, 32.4, 32.3, 26.17, 26.15, 24.7, 24.4, 20.3, 20.0, 12.8, 12.4. ^{19}F NMR (367 MHz, DMF-d$_7$): δ (ppm) = –109.4 (m, 4F, ap/p), –130.5 (m, 2F, ap/p).

HRMS (ESI+, *m/z*): M^{2+} calcd for $C_{49}H_{51}F_6N_3O_7S_2^{2+}$, 485.6531; found, 485.6539.

Synthesis of compound **DAE-NHS**

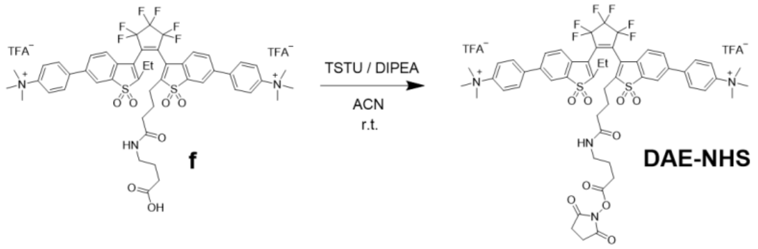

To an ACN solution (1 mL) containing compound **f** (20 mg, 17 μmol) and TSTU (7.5 mg, 25 μmol, 1.5 equiv.), was added DIPEA (7.8 mg, 60 μmol, 3.6 equiv.). The reaction mixture was stirred at r.t. for 30 min. Purification was carried out by preparative HPLC with gradient elution (0.1% aq. TFA / ACN,

95/5 → 50/50). Lyophilization yielded 12 mg (56%) of compound **DAE-NHS** as a yellow powder. ap:p = 7:3.

^{1}H-NMR (400 MHz, DMF-d$_7$): δ (ppm) = 8.55 (dd, *J* = 15.1, 1.7 Hz, 1.4H, ap), 8.46 (s, 0.6H, p), 8.38 – 8.31 (m, 4H), 8.30 – 8.22 (m, 4H), 8.21 – 7.86 (m, 4H), 3.95 (d, *J* = 2.5 Hz, 12.6H, ap), 3.91 (d, *J* = 1.9 Hz, 5.4H, p), 3.37 – 3.19 (m, 2H, ap/p), 2.96 – 2.93 (m, 4H, ap/p), 2.89 – 2.54 (m, 5H, ap/p), 2.51 – 1.57 (m, 7H, ap/p), 1.43 (t, *J* = 7.5 Hz, 0.9H, p), 1.05 (t, *J* = 7.5 Hz, 2.1H, ap). ^{13}C NMR (101 MHz, DMF-d$_7$): δ (ppm) = 172.58, 172.55, 171.4, 171.3, 170.14, 170.12, 150.2, 149.1, 149.0, 142.73, 142.68, 140.4, 140.3, 137.5, 137.4, 134.6, 130.0, 129.92, 129.85, 129.82, 125.9, 125.8, 124.5, 123.9, 122.6, 122.5, 122.1, 57.72, 57.67, 39.0, 29.2, 26.7, 26.4, 26.1, 25.9, 25.8, 24.3, 20.0, 12.8, 12.4. ^{19}F NMR (367 MHz, DMF-d$_7$): δ (ppm) = –109.4 (m, 4F, ap/p), –130.5 (m, 2F, ap/p).

HRMS (ESI+, *m/z*): M^{2+} calcd for $C_{53}H_{54}F_6N_4O_9S_2^{2+}$, 534.1613; found, 534.1613.

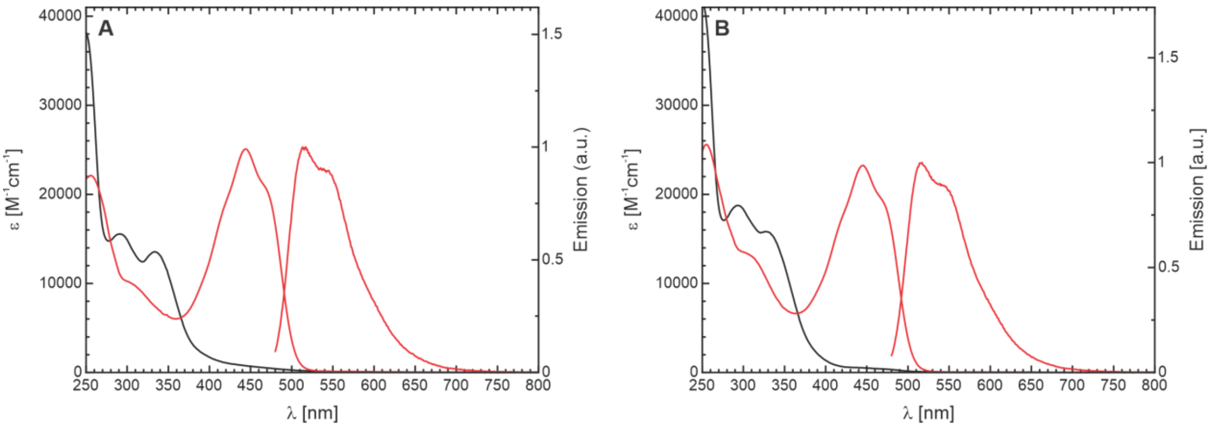

Figure S1. Absorption spectra (extinction coefficients) and normalized emission spectra of compounds **DAE** (A) and **DAE-COOH** (B) in aqueous solutions. The black and red lines are the spectra of open and closed isomers, respectively.

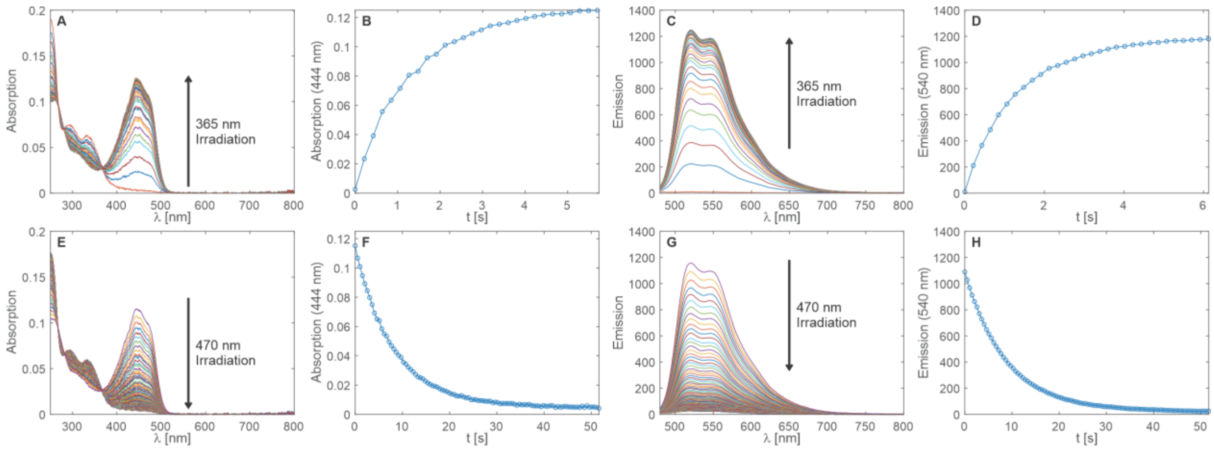

Figure S2. Absorption and emission changes of an aqueous solution of compound **DAE** (5.4 µM) under irradiation with 365 nm light (A-D) and 470 nm light (E-F).

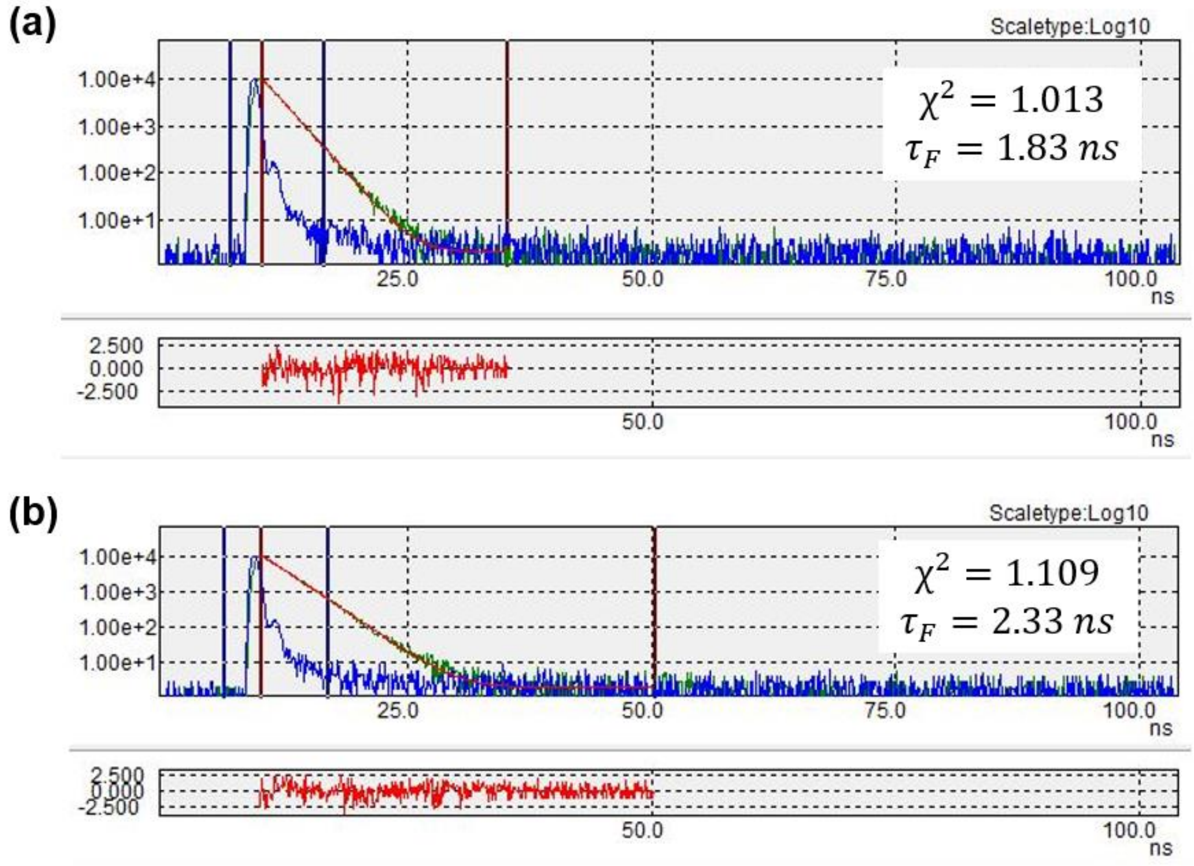

Figure S3. Lifetime decay profiles of (a) **DAE** and (b) **DAE**@CB7 complex in aqueous solution. The excitation and emission wavelengths were 405 nm and 555 nm, respectively. The green, blue, and red lines represent lifetime decay, instrumental response functions (IRF), and fitted curve, respectively. The lower red line represents the residual.

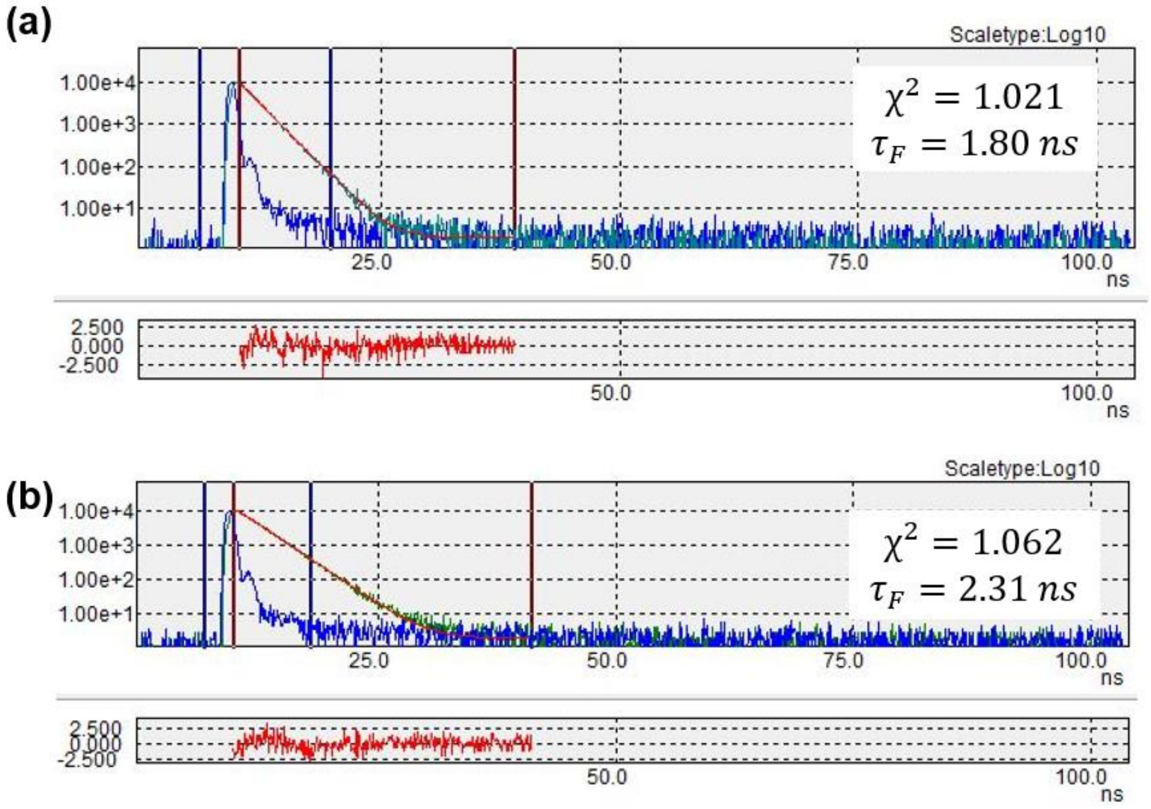

Figure S4. Lifetime decay profiles of (a) **DAE** and (b) **DAE**@CB7 complex in aqueous solution after argon purging for 15 min. The excitation and emission wavelengths were 405 nm and 555 nm, respectively. The green, blue, and red lines represent lifetime decay, instrumental response functions (IRF), and fitted curve, respectively. The lower red line represents the residual.

Table S1. Fluorescence quantum yields (Φ_{fl}), fluorescence lifetimes (τ_{fl}), radiative (k_r) and non-radiative (k_{nr}) rate constants of free **DAE** and **DAE**@CB7 complex at the photostationary state upon 365 nm irradiation in aqueous solution before and after argon purging.

	**DAE**				**DAE**@CB7			
	Φ_{fl}	τ_{fl} (ns)	k_r (s^{-1})	k_{nr} (s^{-1})	Φ_{fl}	τ_{fl} (ns)	k_r (s^{-1})	k_{nr} (s^{-1})
H_2O	0.40	1.83	2.19E8	3.28E8	0.63	2.33	2.70E8	1.59E8
H_2O (Ar purged)	0.41	1.80	2.28E8	3.28E8	0.63	2.31	2.73E8	1.60E8

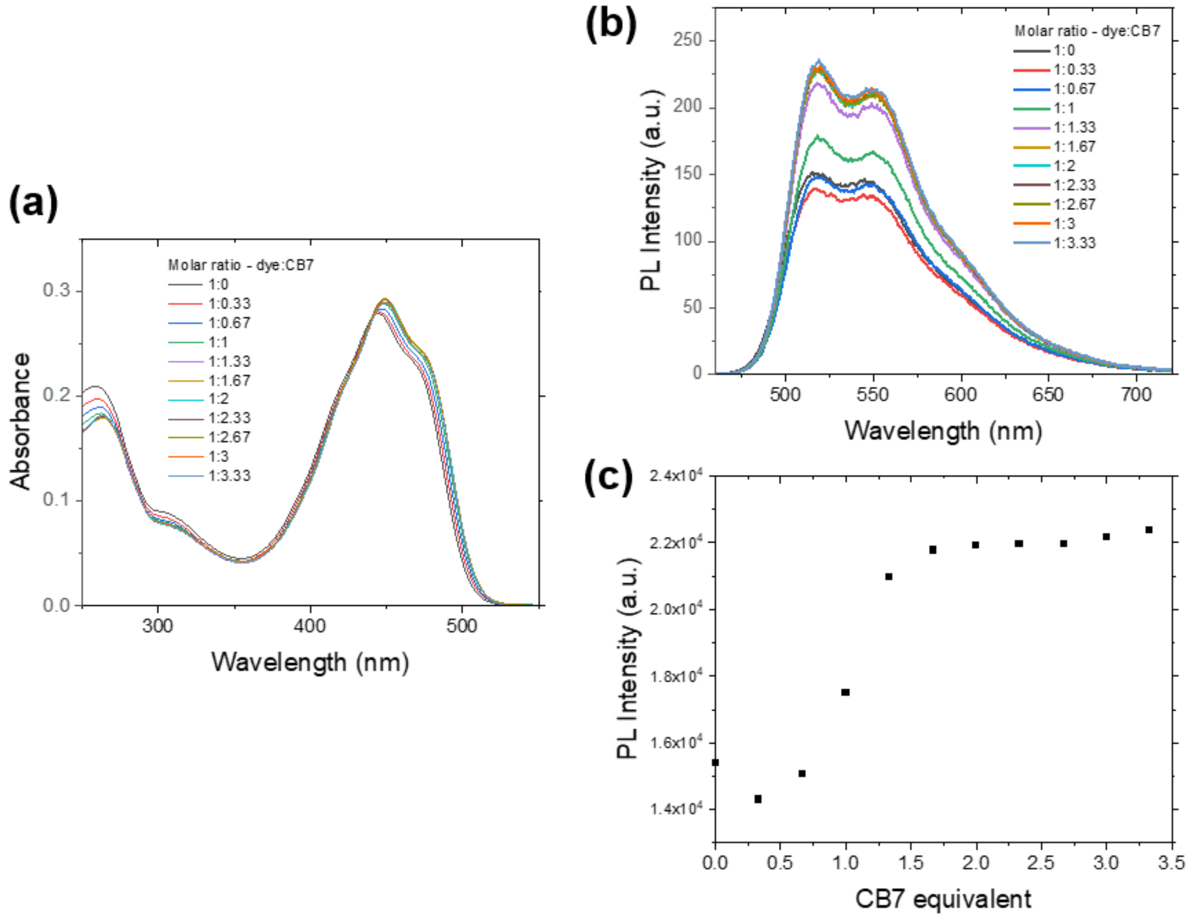

Figure S5. (a) UV/vis and (b) PL spectra of **DAE** (10^{-5} M) in water upon CB7 addition. (c) PL intensity changes upon CB7 addition.

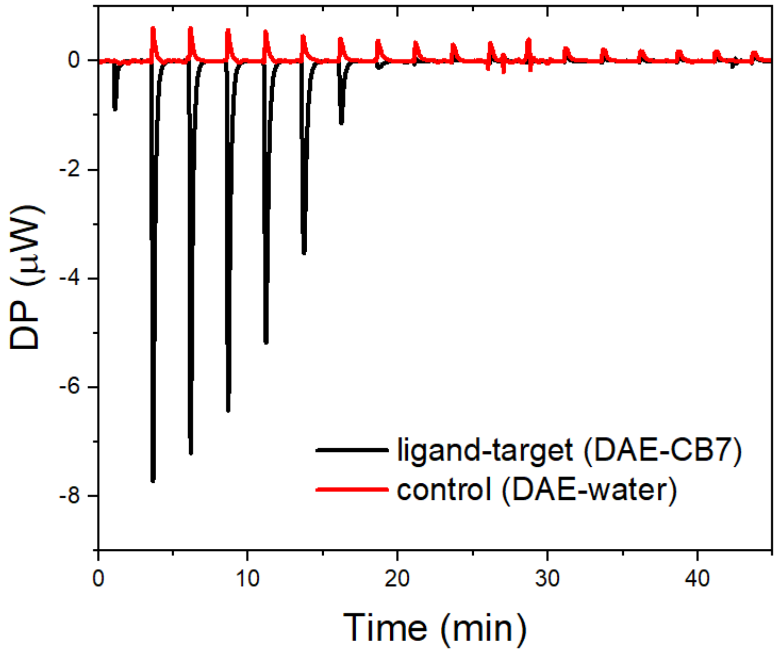

Figure S6. The raw data of the isothermal titration calorimetry (ITC) measurements: thermographs of titration of 1000 μM **DAE** into 100 μM CB7 (black line) and titration of 1000 μM **DAE** into pure water (red line). The ligand-target binding signal was subtracted by control signal, and used for Figure 2b in the main text.

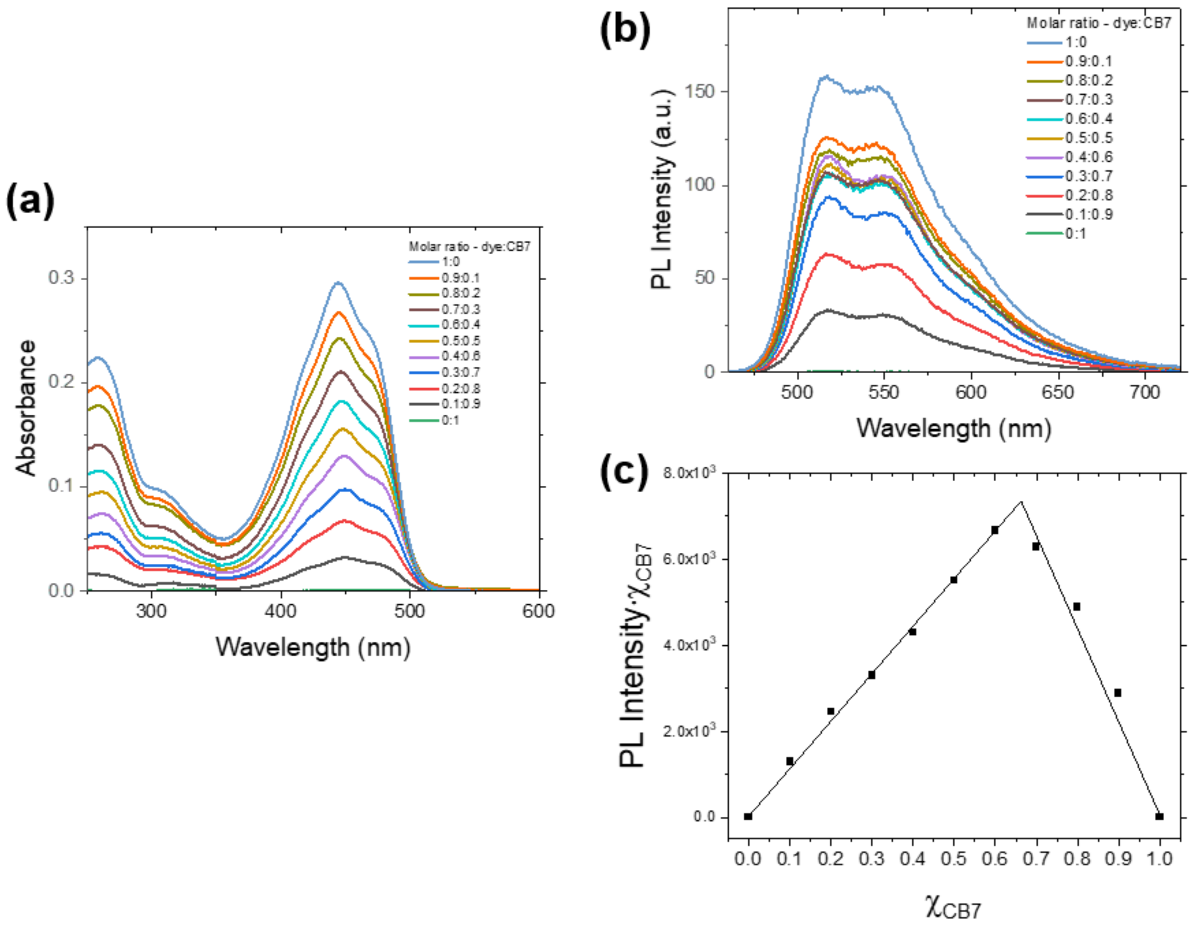

Figure S7. (a) UV/vis and (b) PL spectra of the mixed solutions of **DAE** and CB7 in water, [DAE]+[CB7]=10^{-5} M; (c) the Job's plot for complexation of **DAE** with CB7, derived from PL spectra (b), PL intensity: emission area, χ_{CB7}: molar fraction of CB7 in a mixture.

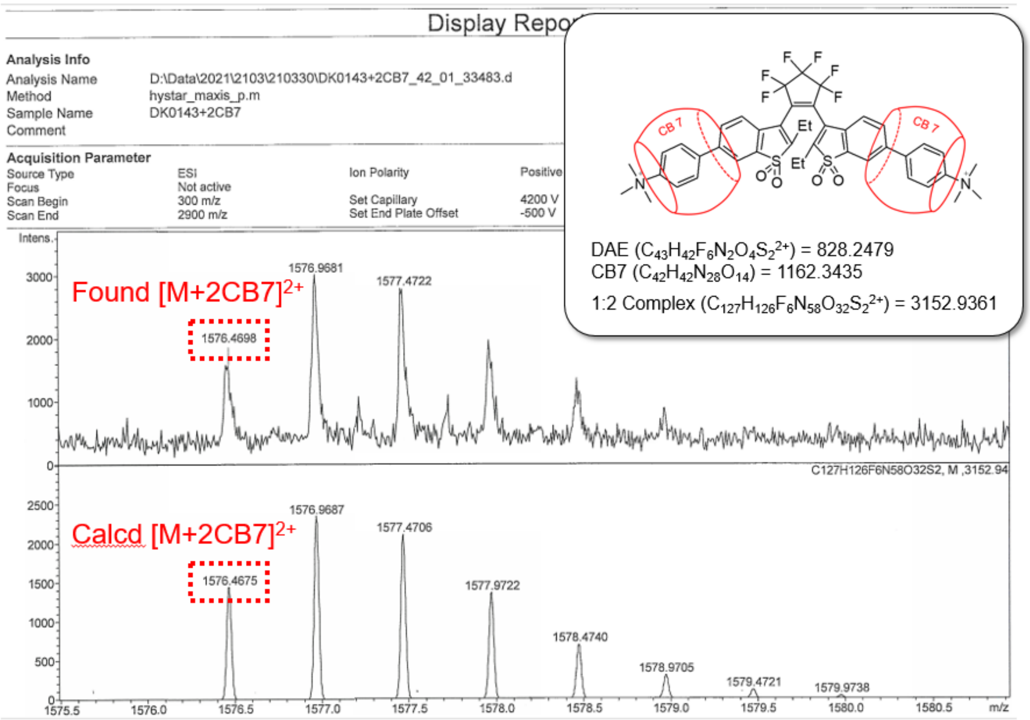

Figure S8. High resolution mass-spectrum (HRMS) of **DAE**@CB7; a peak with *m/z* = 1576.4698 supports 1:2 stoichiometry of the **DAE**@CB7 complex.

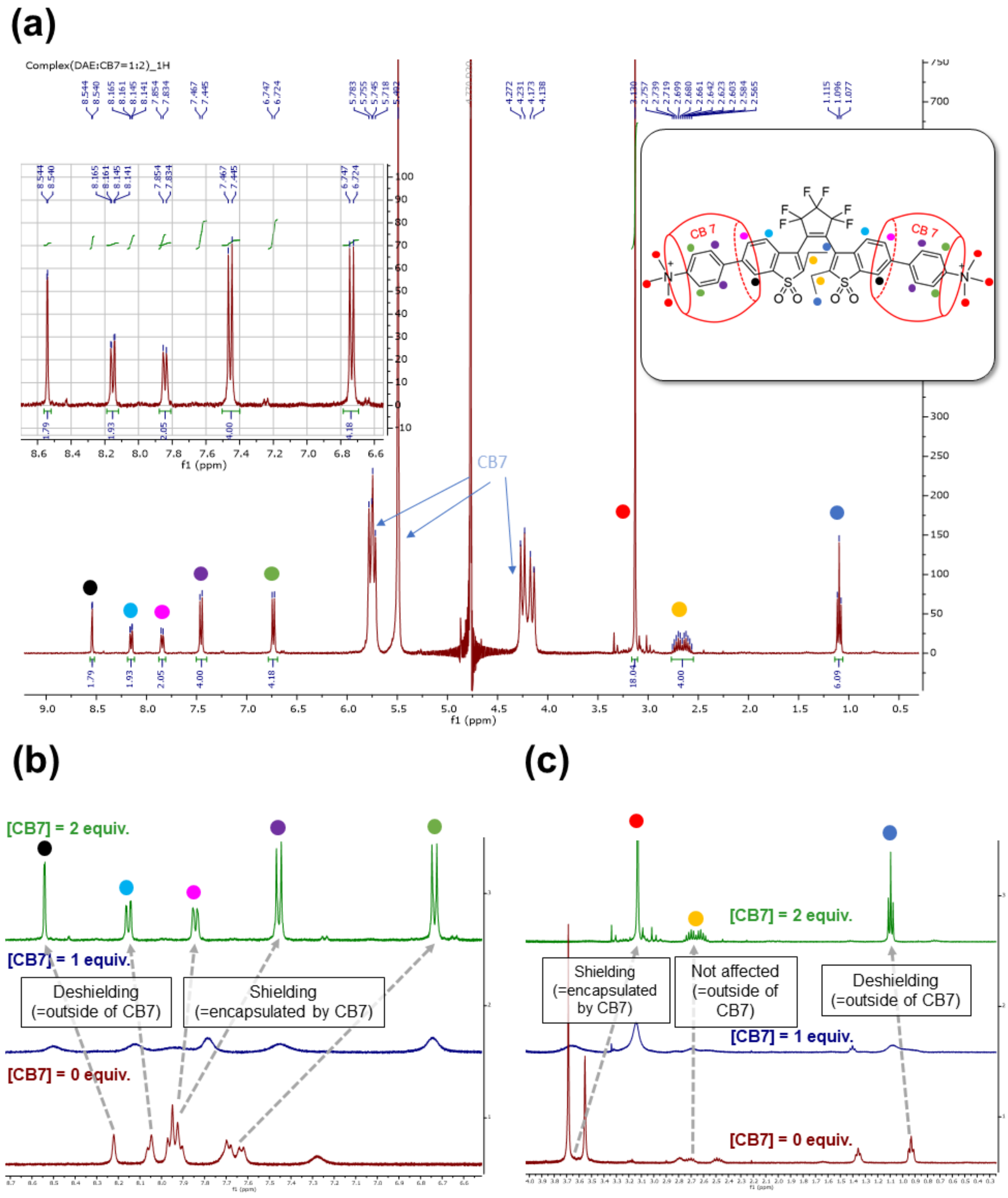

Figure S9. (a) ^{1}H NMR spectra of **DAE@CB7** complex in D_2O. CB7 titration induced changes in the resonance signals: (b) ^{1}H NMR aromatic region and (c) ^{1}H NMR aliphatic region of **DAE** in D_2O. [DAE] = 2 mM. The changes of the chemical shifts were observed for 10 out of total 14 aromatic protons. Shielding = encapsulation by CB7. The chemical shifts of ethyl groups at C-2/C-2' did not change considerably; these groups are situated outside of CB7. One set of signals of ethyl groups in the complex indicate the presence of only one (anti-parallel) conformer.

	Open isomer		Closed isomer
	Anti-parallel	Parallel	
**Front view**	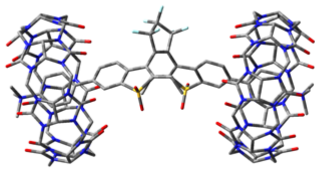	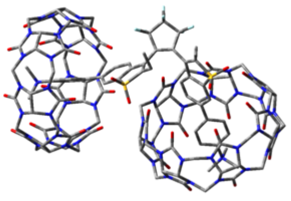	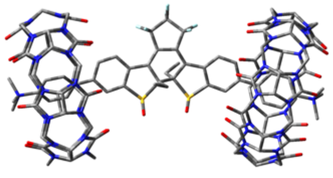
**Side view**	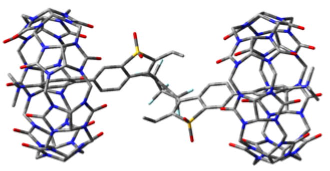	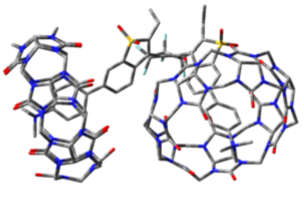	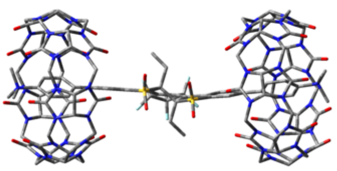
**Energy (kcal/mol)**	**+3.523**	**+8.731**	**0 (reference)**

Figure S10. Results of the DFT geometry optimization (B3LYP/6-31G) in water (CPCM) for the anti-parallel conformer, parallel conformer, and the closed-ring isomer of **DAE** in the CB7 complex: **DAE(ap)**@CB7, **DAE(p)**@CB7, and **DAE(c)**@CB7. All energies are given in *kcal/mol*, relative to the closed-ring isomer, **DAE(c)**@CB7.

	Open isomer		Closed isomer
	Anti-parallel	Parallel	
Front view	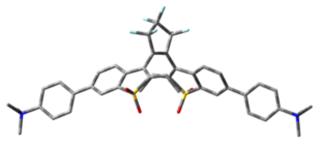	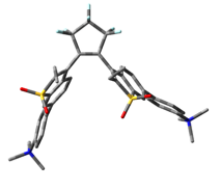	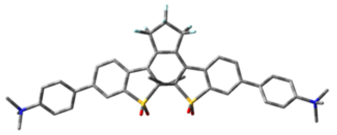
Side view	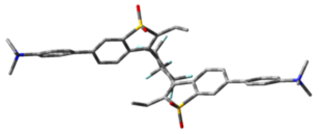	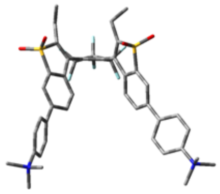	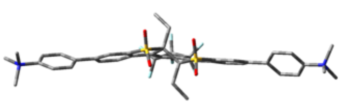
Energy (kcal/mol)	+4.214	+4.861	0 (reference)

Figure S11. Results of the DFT geometry optimization (B3LYP/6-31G) in water (CPCM) for the anti-parallel conformer of the open-ring isomer, parallel conformer of the open-ring isomer, and the closed-ring isomer of free **DAE**: **DAE(ap)**, **DAE(p)**, and **DAE(c)**. All energies are given in *kcal/mol* relative to the closed-ring isomer, **DAE(c)**.

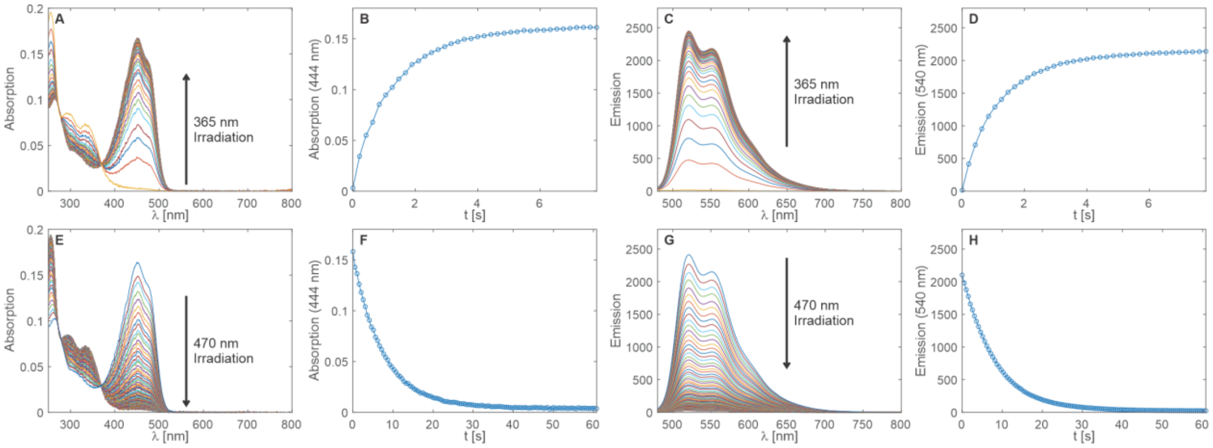

Figure S12. Absorption and emission changes of an aqueous solution of compound **DAE** (5.4 µM) containing CB7 (4 eq.) under irradiation with 365 nm light (A-D) and 470 nm light (E-F).

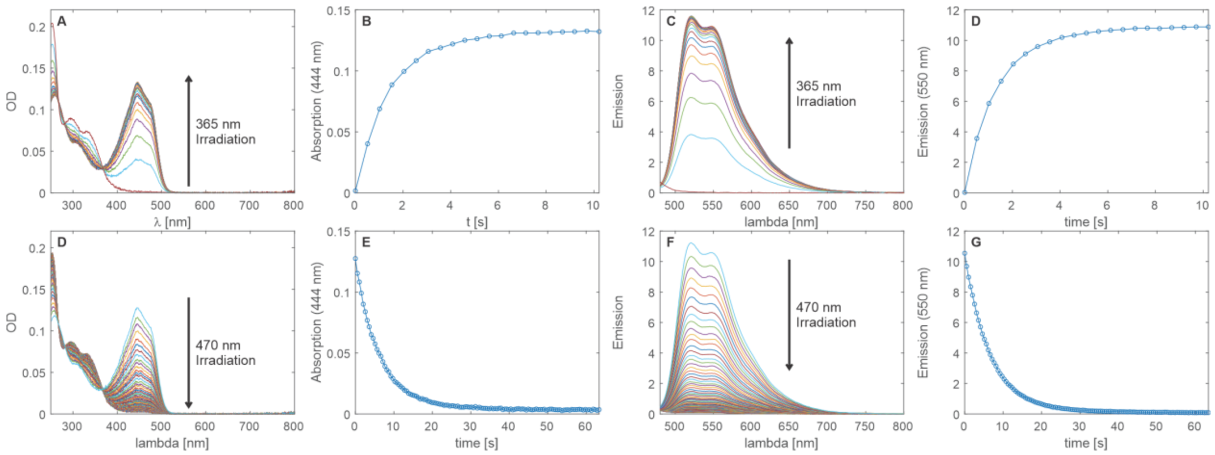

Figure S13. Absorption and emission changes of an aqueous solution of compound **DAE-COOH** (5.4 μM) under irradiation with 365 nm light (A-D) and 470 nm light (E-F)).

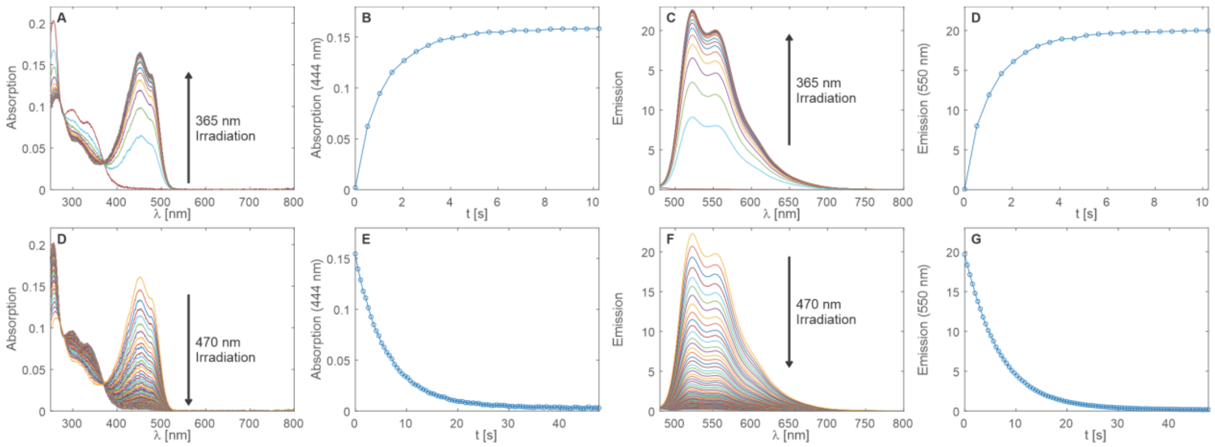

Figure S14. Absorption and emission changes of an aqueous solution of compound **DAE-COOH** (5.4 μM) containing CB7 (4 eq.) under irradiation with 365 nm light (A-D) and 470 nm light (E-F)).

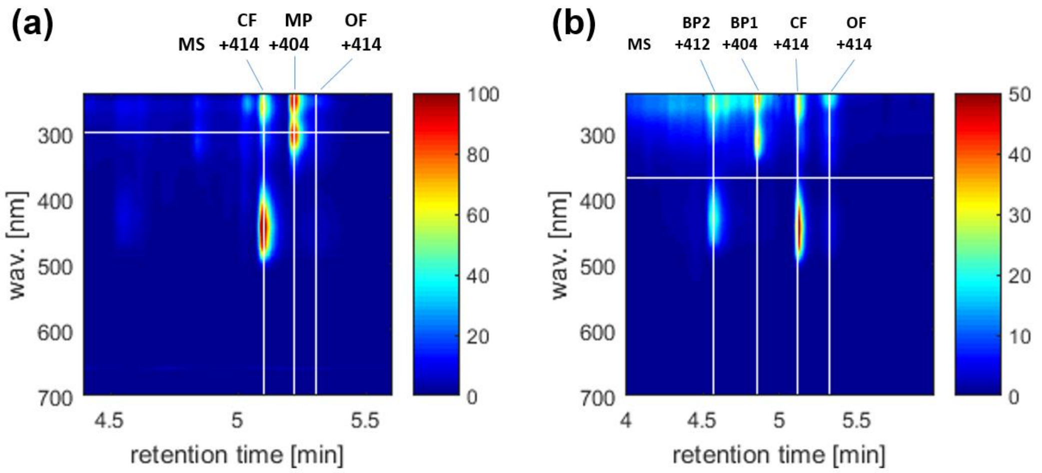

Figure S15. 2D maps of LC-MS (HPLC with the molecular mass detection; positive mode ESI-MS) after repetitive photoswitching experiments: (a) free **DAE** and (b) **DAE**@CB7 were irradiated with 365 nm light in aqueous solutions. OF: open form, CF: closed form, MP: main byproduct, BP1: main byproduct 1, and BP2: main byproduct 2. The molecular masses of MP and BP1 (808), as well as BP2 (824), correspond to –2F+H_2O, as well as –2F+2OH transformations of the $(CF_2)_3$ bridge.

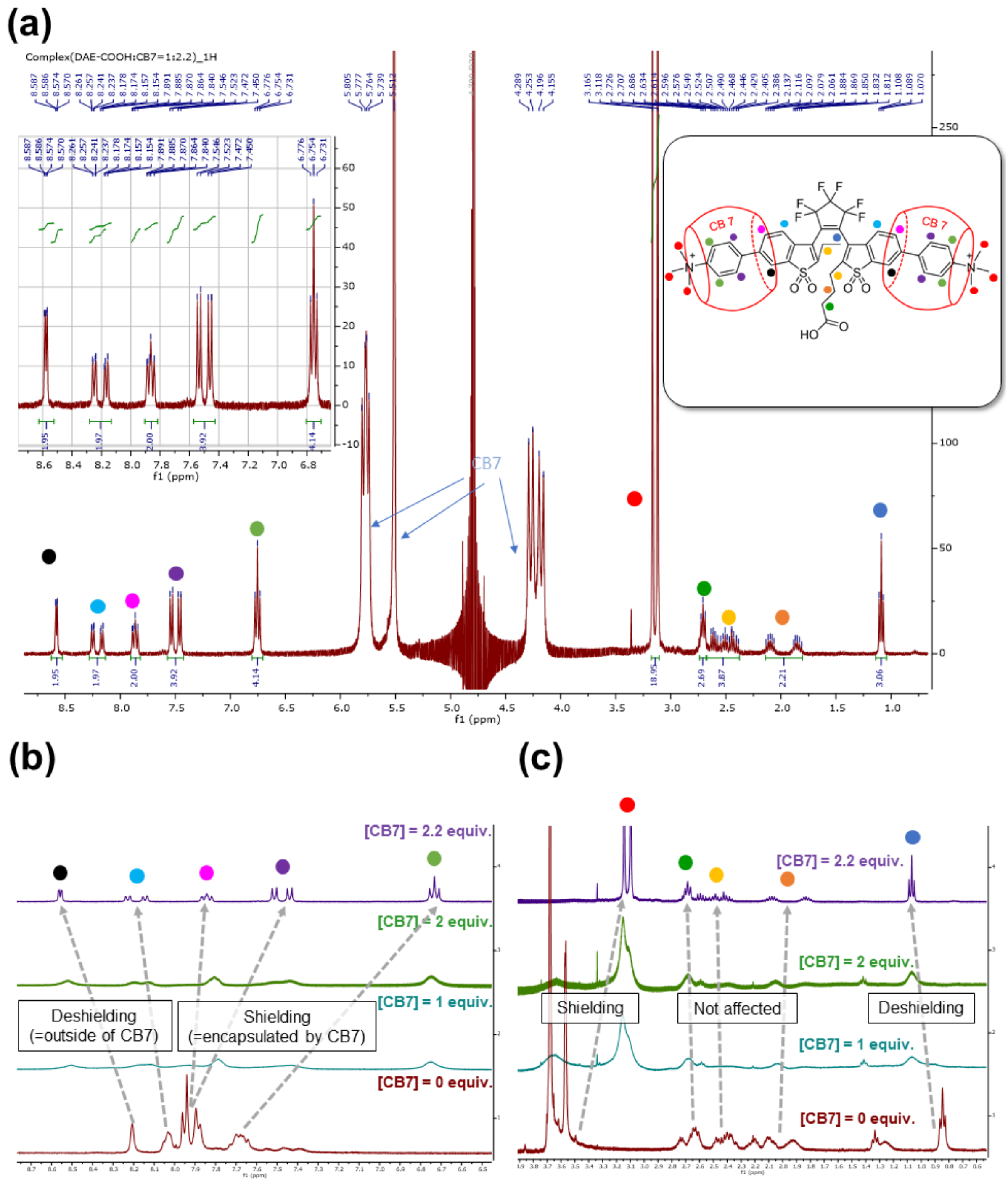

Figure S16. (a) ^{1}H NMR spectra of **DAE-COOH**@CB7 complex in D_2O. CB7 titration induced changes in the chemical shifts: (b) ^{1}H NMR aromatic region and (c) ^{1}H NMR aliphatic region of **DAE-COOH** in D_2O. [DAE-COOH] = 2 mM. The signals of 10 out of total 14 aromatic protons are shifted (due to encapsulation by CB7). The positions of the signals of CH_2 groups (ethyl and carboxyalkyl groups) are not particularly affected, as these residues are located outside of CB7. The 1:2 complex **DAE-COOH**@CB7 displays one set of signals (of an anti-parallel conformer).

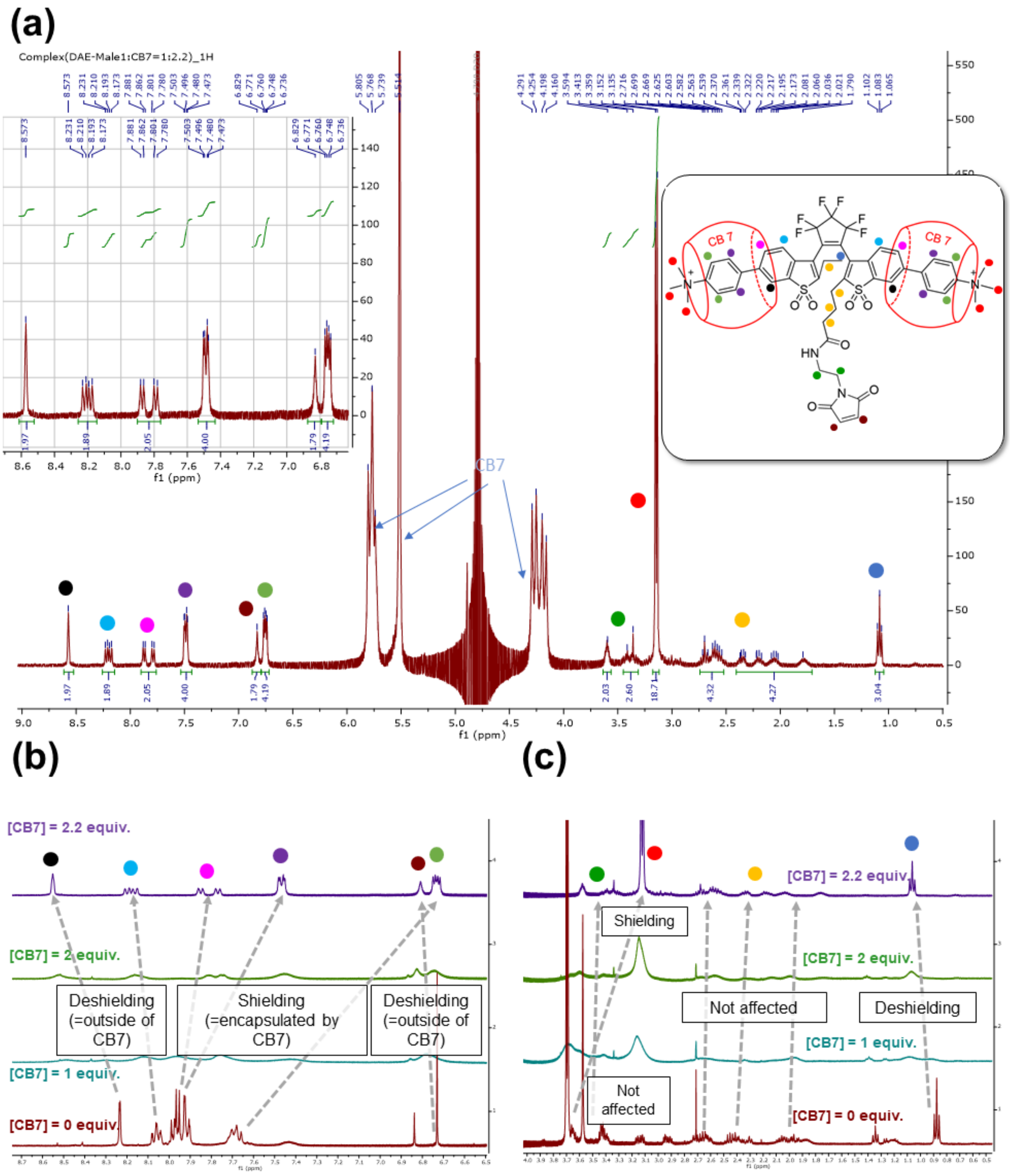

Figure S17. (a) ^{1}H NMR spectra of **DAE-Male1**@CB7 complex in D_2O. CB7 titration induced changes in the resonance signals: (b) ^{1}H NMR aromatic region and (c) ^{1}H NMR aliphatic region of **DAE-Male1** in D_2O. [DAE-Male1] = 2 mM. The signals of 10 out of total 14 aromatic protons are shifted (due to encapsulation by CB7). The positions of the signals of CH_2 groups (ethyl and amidoalkyl groups) are not particularly affected, as these residues are located outside of CB7. **DAE-Male1**@CB7 (1:2 complex) displays one set of signals(of an anti-parallel conformer).

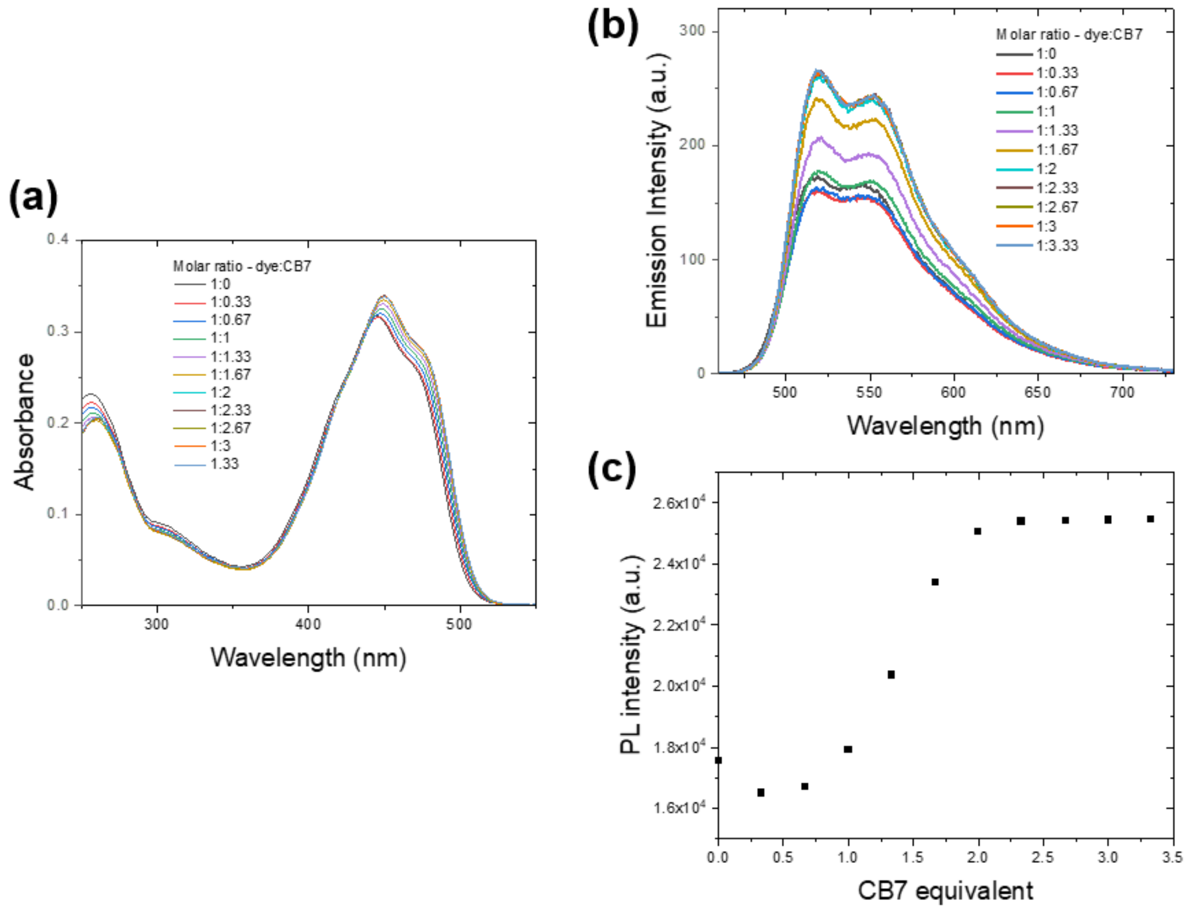

Figure S18. (a) UV/vis and (b) PL spectra of **DAE-COOH** (10^{-5} M) in aqueous solutions upon CB7 addition. (c) PL intensity change upon CB7 addition.

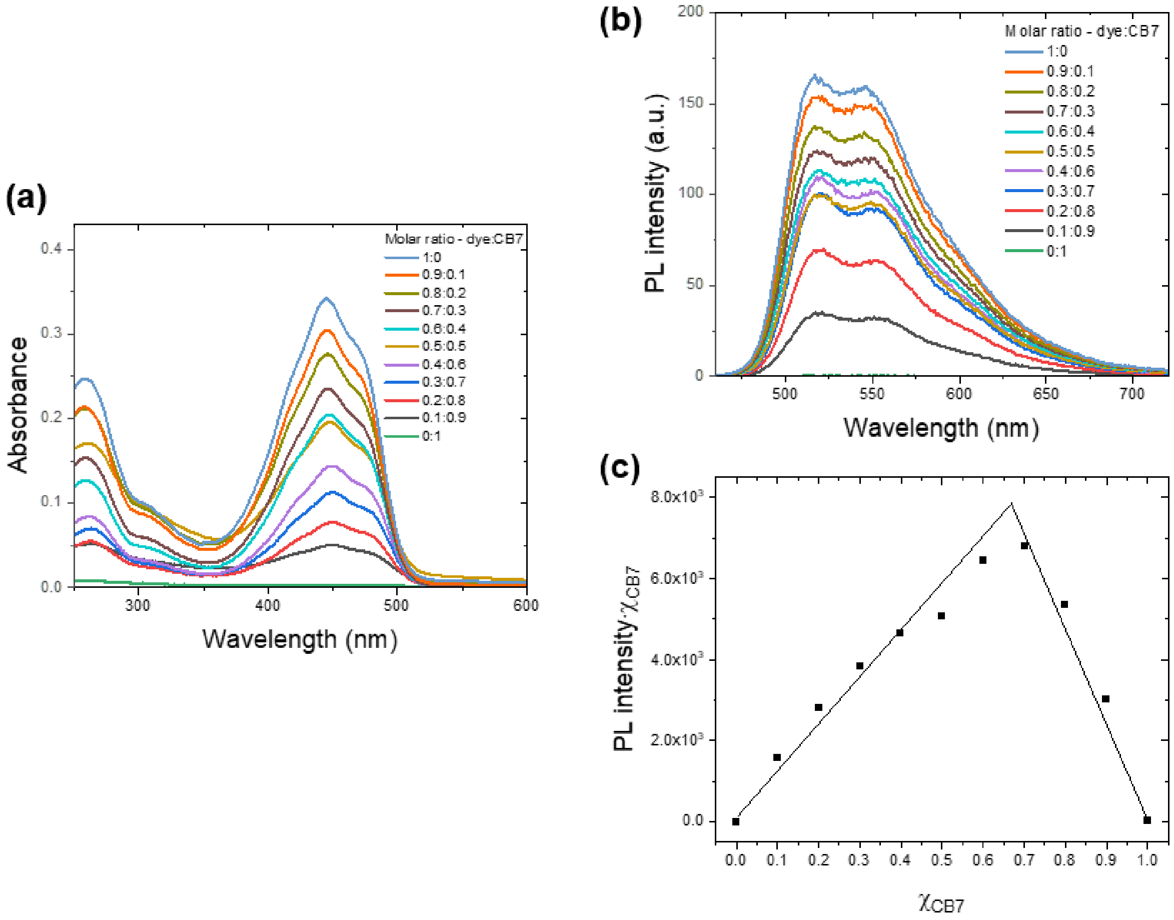

Figure S19. (a) UV/vis and (b) PL spectra of the aqueous solutions containing **DAE-COOH** and CB7, [DAE-COOH]+[CB7]=10^{-5} M. (c) the Job's plot for complexation of **DAE-COOH** with CB7 derived from the PL spectra (b), PL intensity: emission area, χ_{CB7}: molar fraction of CB7 in a mixture.

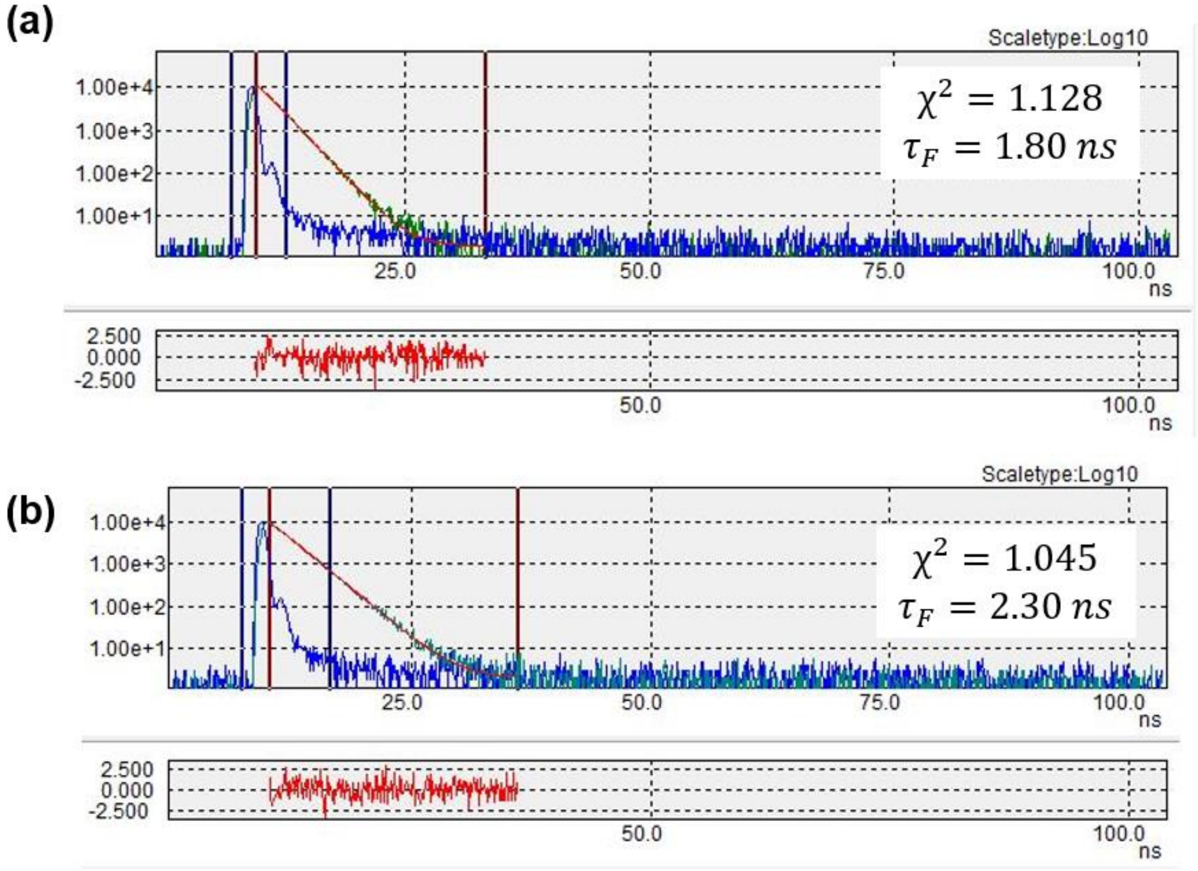

Figure S20. Lifetime decay profiles of (a) **DAE-COOH** and (b) **DAE-COOH**@CB7 complex in aqueous solution. The excitation and emission wavelengths were 405 nm and 555 nm, respectively. The green, blue, and red lines represent lifetime decay, IRF, and fitted curve, respectively. The lower red line represents the residual.

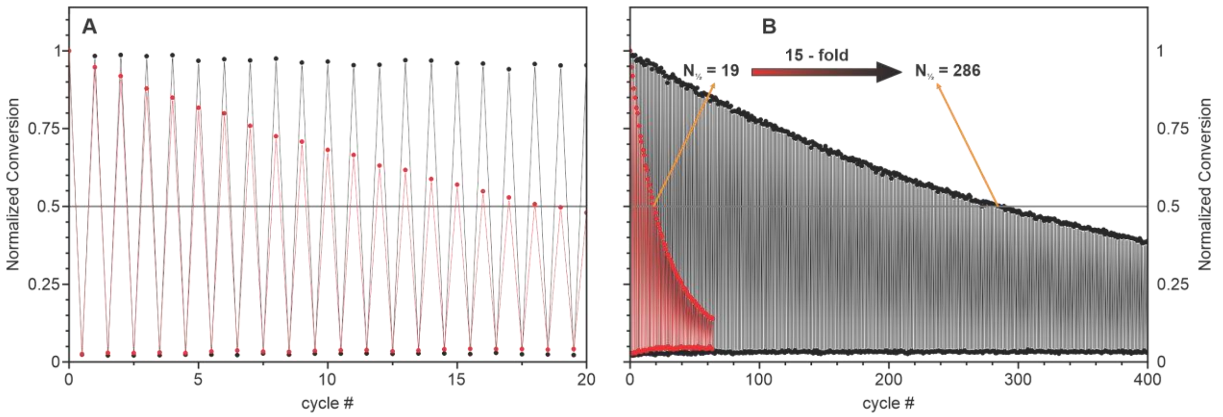

Figure S21. Reversible photoswitching cycles of free **DAE-COOH** (red) and **DAE-COOH**@CB7 (black) complex dissolved in pure water: (a) A zoom into the first 20 cycles, (b) Full cycles up to N=400.

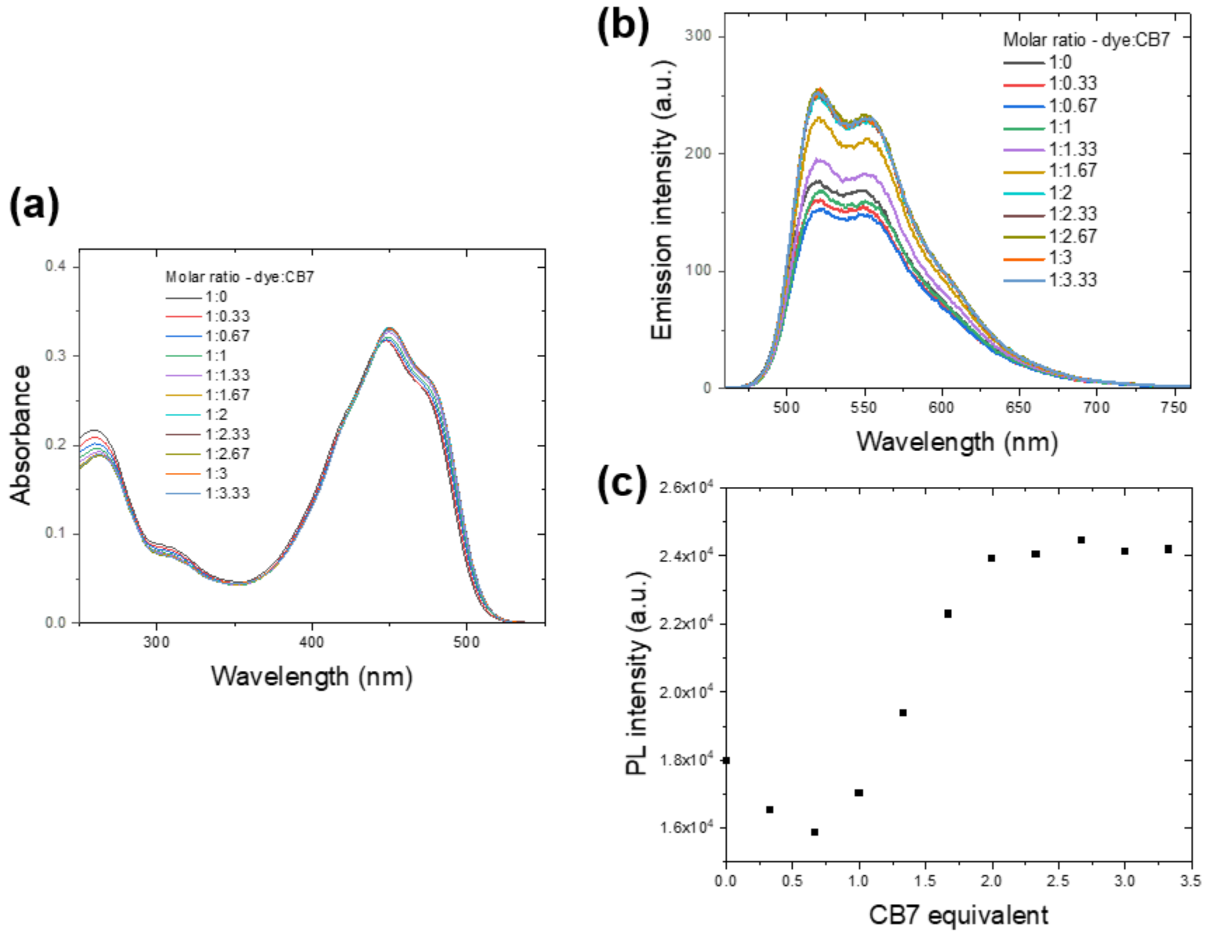

Figure S22. (a) UV/vis and (b) PL spectra of **DAE-Male1** (10^{-5} M) in aqueous solution upon addition of CB7. (c) the PL intensity changes upon CB7 addition.

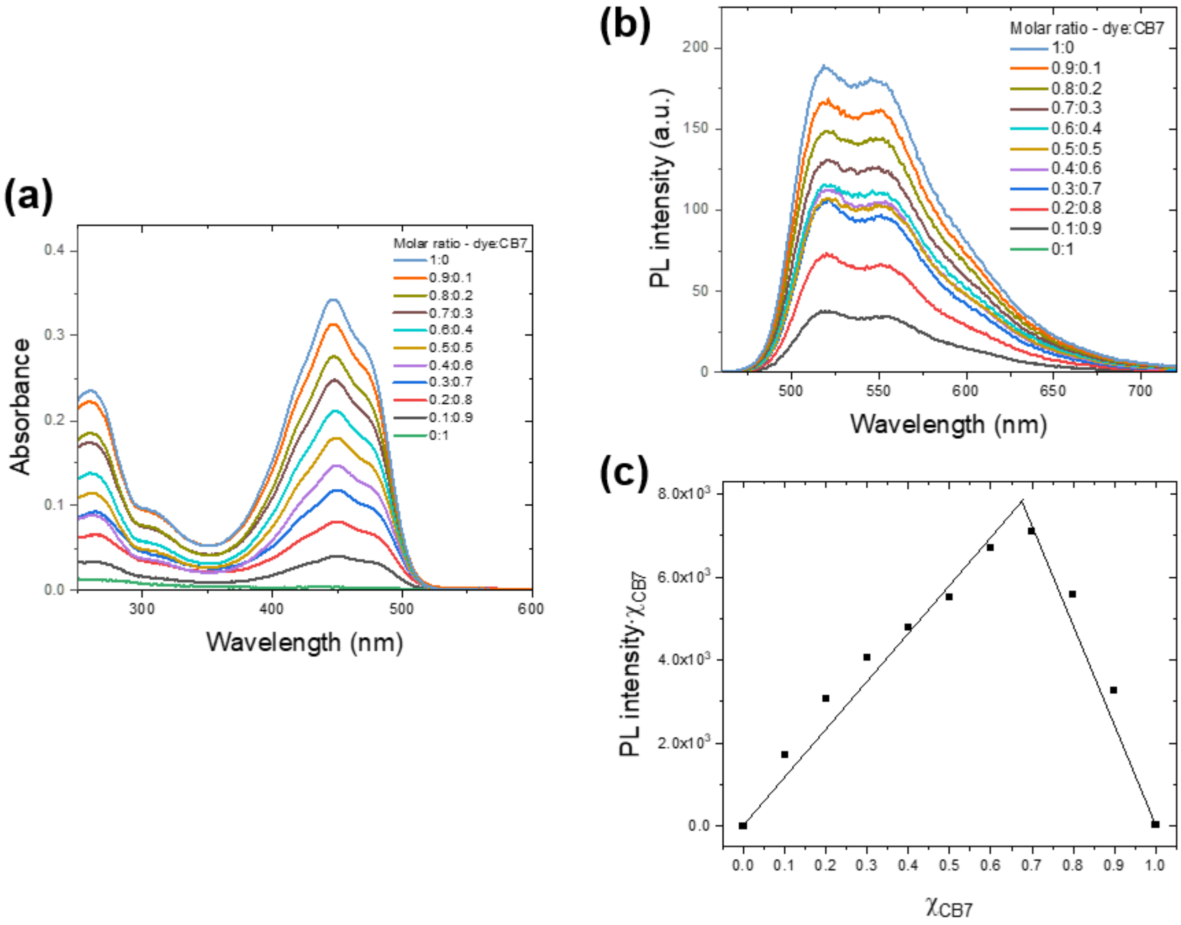

Figure S23. (a) UV/vis and (b) PL spectra of the aqueous solutions: mixtures of **DAE-Male1** and CB7, [DAE-Male1]+[CB7]=10^{-5} M; the (c) Job's plot for complexation of **DAE-Male1** with CB7, derived from the PL spectra (b), PL intensity: emission area, χ_{CB7}: molar fraction of CB7 in a mixture.

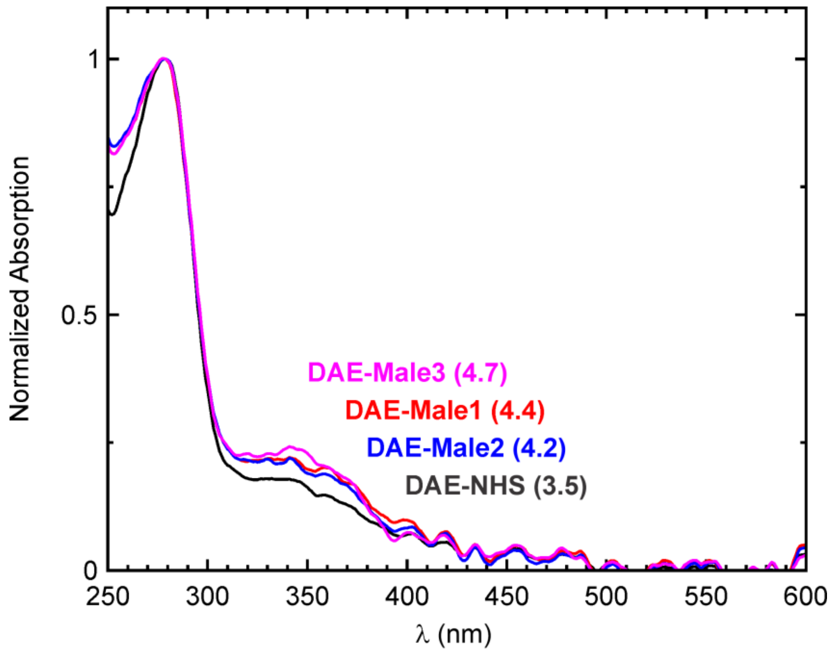

Figure S24. Absorption spectra of the prepared bioconjugates: secondary antibodies labeled with the indicated reactive DAEs. Spectra were normalized to the absorption at 280 nm.

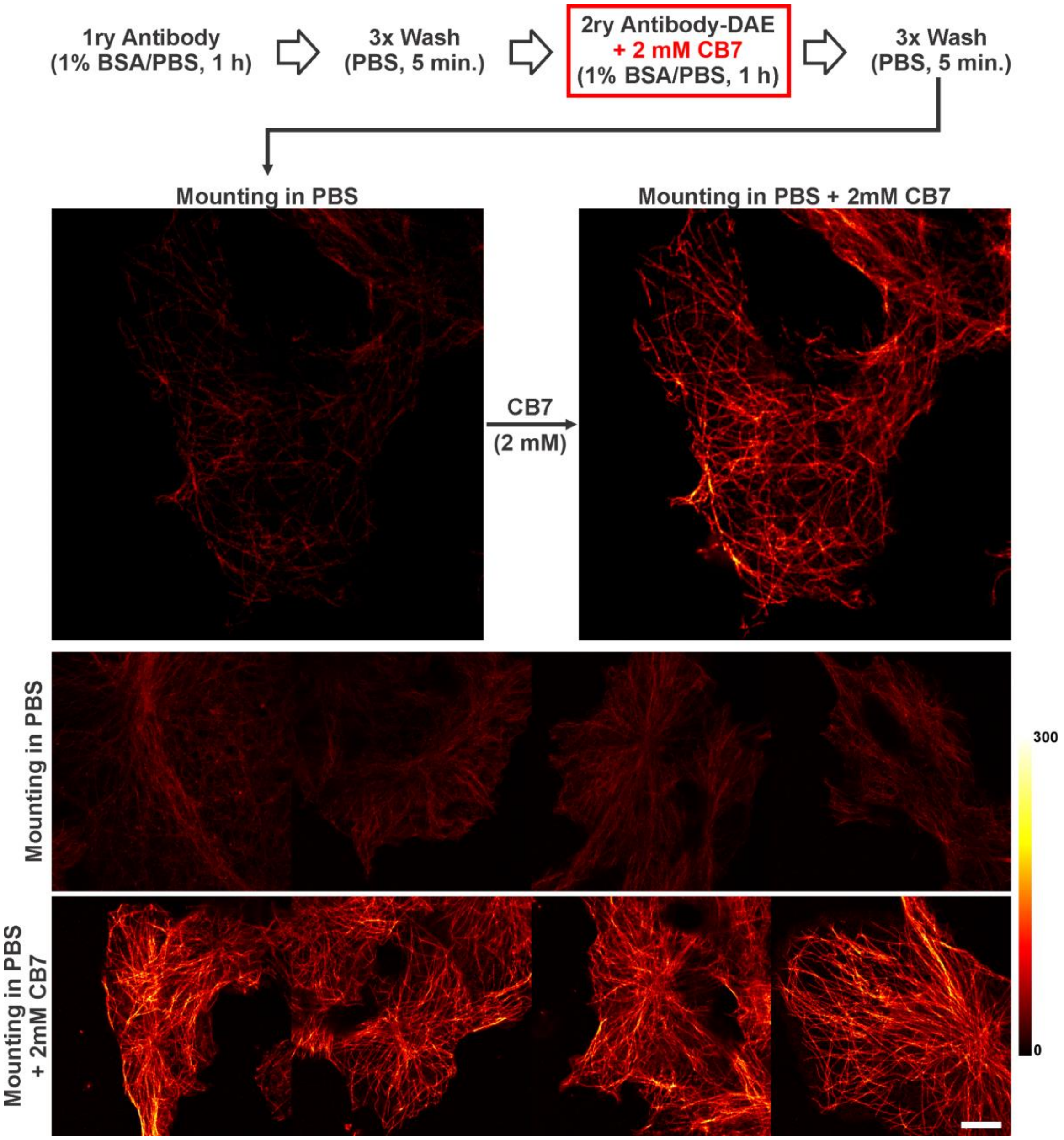

Figure S25. Confocal images of fixed Cos7 cells, stained with secondary antibodies labeled with **DAE-NHS**, with addition of CB7 (2 mM) during the staining with the secondary. A schematic representation of the protocol is presented on the top. The confocal images were obtained by mounting in PBS (middle row). In the bottom row, images of other cells and other fields of view in the same sample are presented after the addition of CB7 (2 mM). The markers are switched-on before the recording of each pixel, with a short pulse of 355 nm light. We conclude from the experiment that the CB7-DAE complex is dissociated during the washing steps with PBS, probably due to the removal of CB7 during the washing step and/or the presence of single-charged cations (Na^+ and K^+) in the PBS buffer. Scale bar: 10 µm

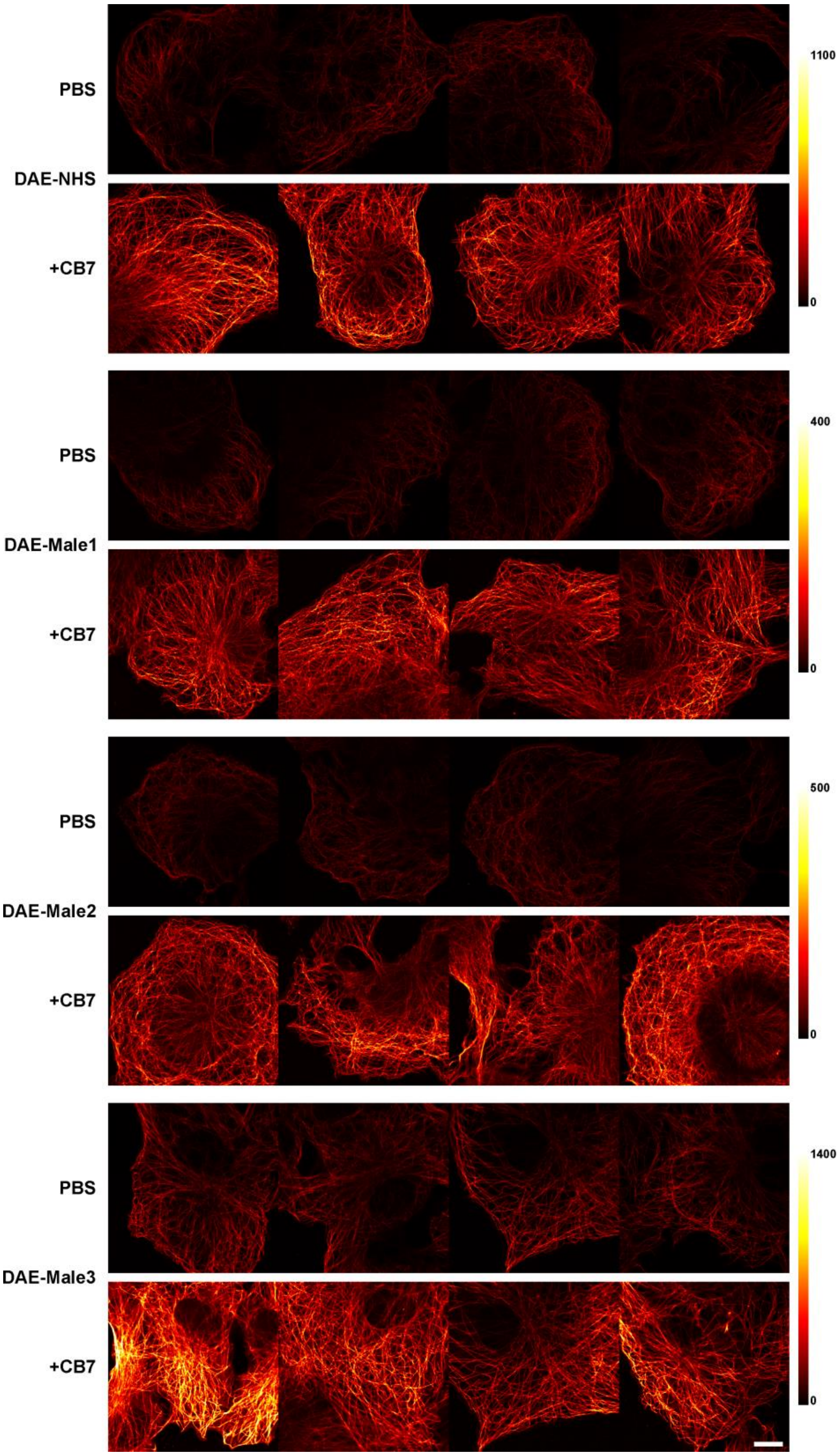

Figure S26. Confocal images of fixed Cos7 cells, stained with secondary antibodies labeled with the indicated DAE before (top) and after (bottom) CB7 addition. The markers are switched-on before the recording of each pixel with a short pulse of 355 nm light. The images relate to Figure 5 in the main text: the same samples and imaging conditions. Scale bar: 10 µm.

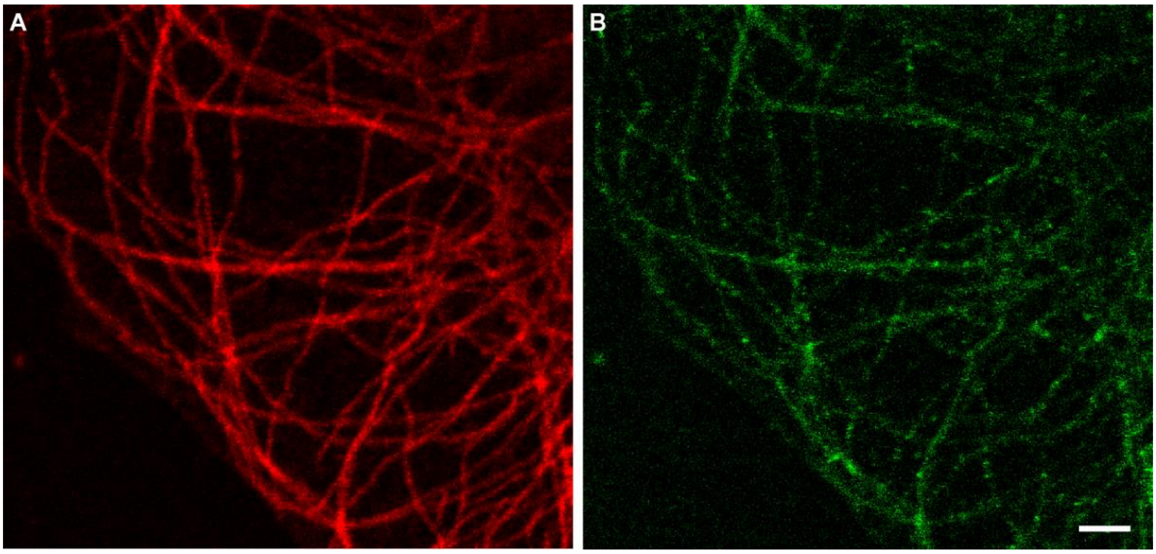

Figure S27. Confocal (A) and RESOLFT (B) images of fixed Cos7 cells, stained with secondary antibodies labeled with **DAE-Male3**; mounted in PBS *without CB7*. The image here corresponds to the image displayed in Figure 7: the same sample and imaging conditions. Scale bar: 2 µm.

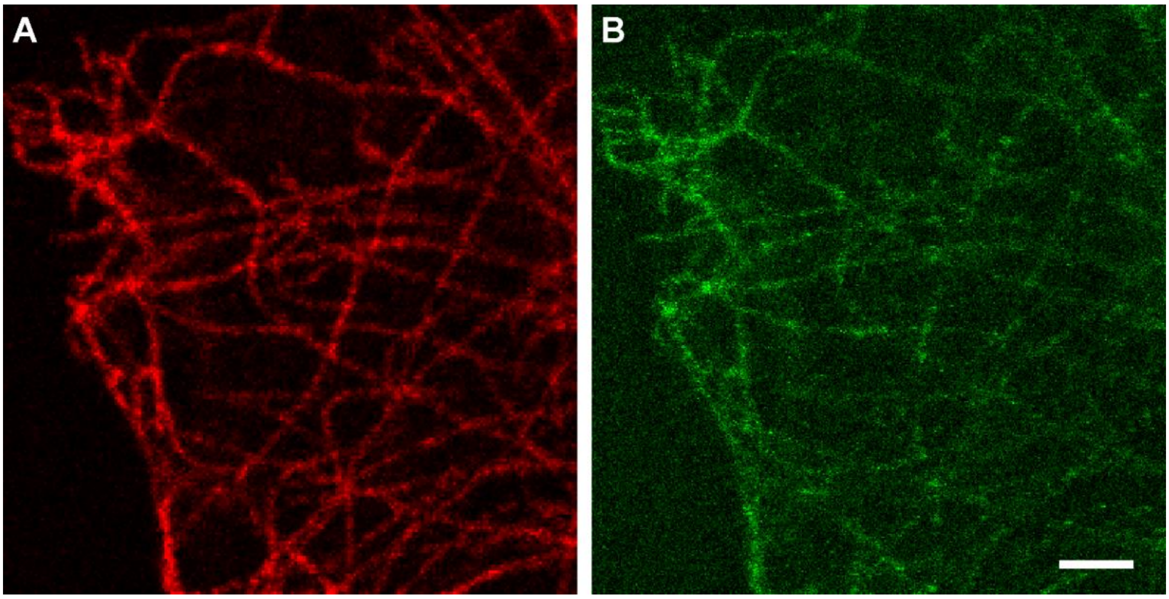

Figure S28. Confocal (A) and RESOLFT (B) images of fixed Cos7 cells, stained with secondary antibodies labeled with **DAE-NHS**, mounted in PBS *without CB7*. The image correspond to the one displayed in Figure 7: the same sample and imaging conditions. Scale bar: 2 µm.

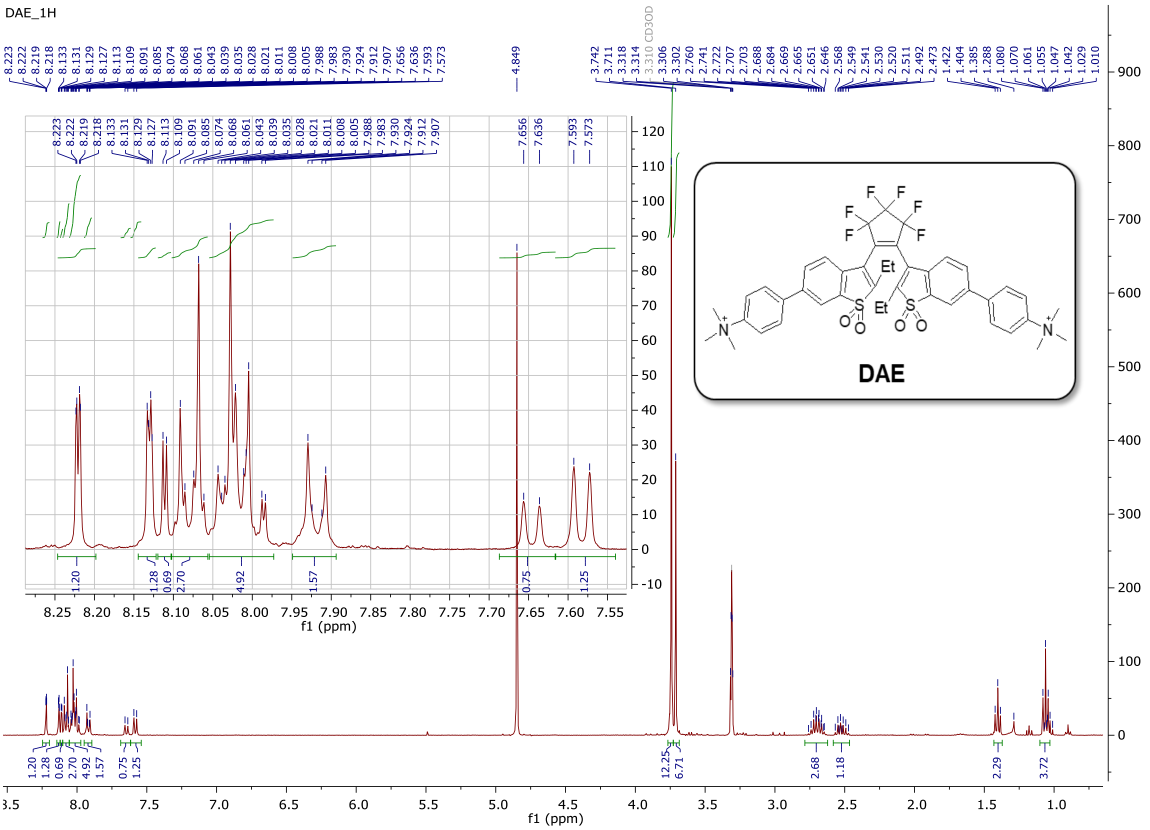

Figure S29. ^{1}H NMR spectrum (400 MHz, CD_3OD) of **DAE**.

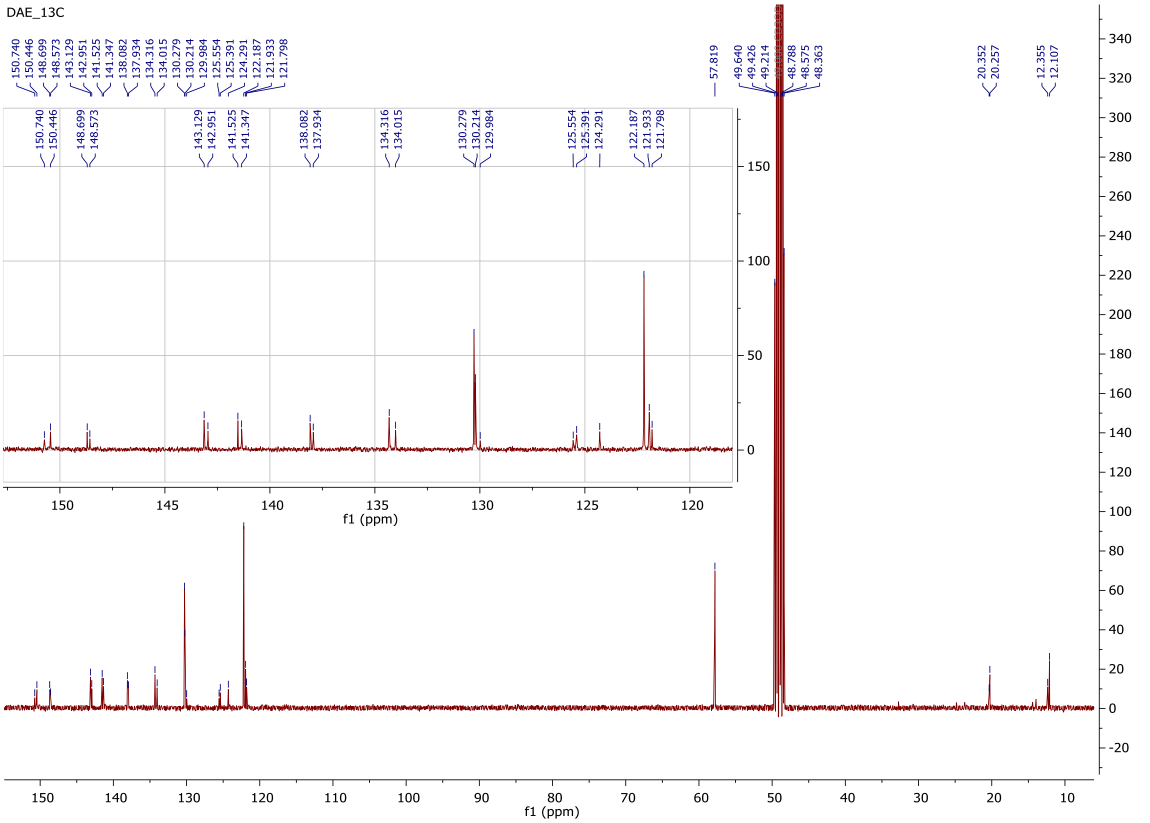

Figure S30. ^{13}C NMR spectrum (101 MHz, CD_3OD) of **DAE**.

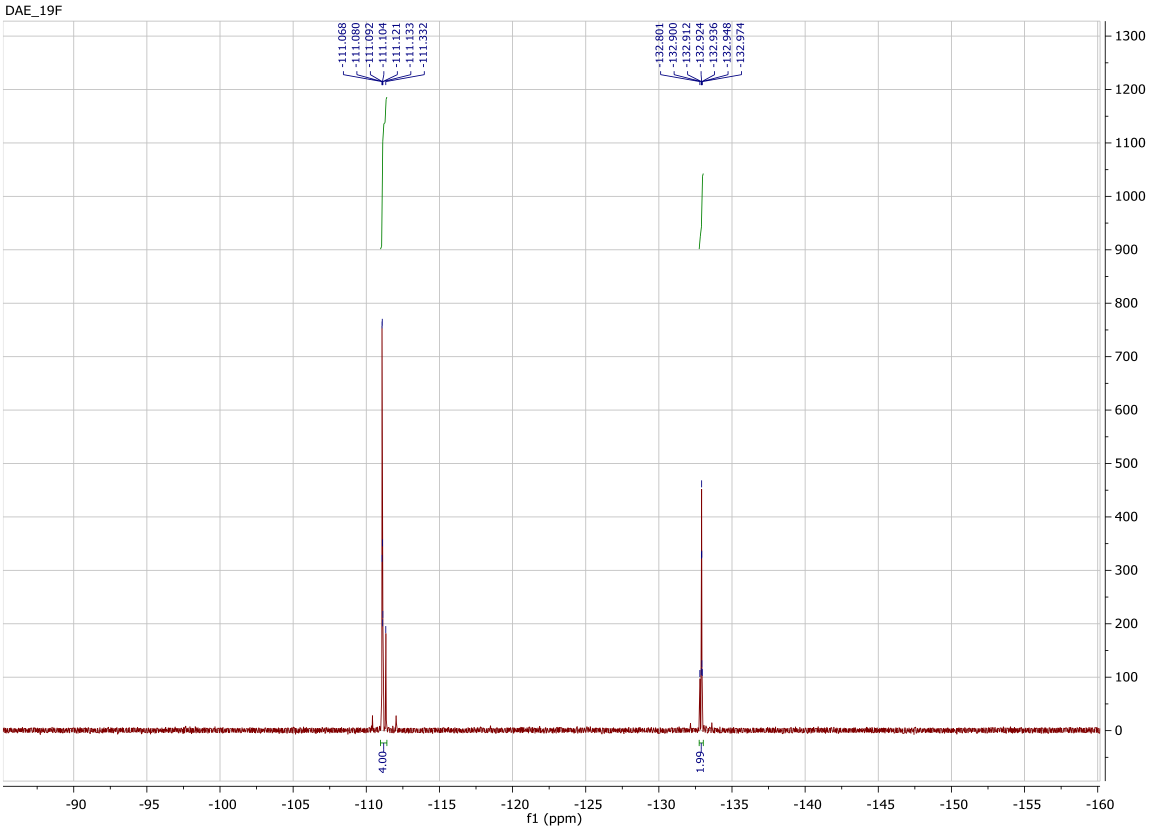

Figure S31. ^{19}F NMR spectrum (367 MHz, CD_3OD) of **DAE**.

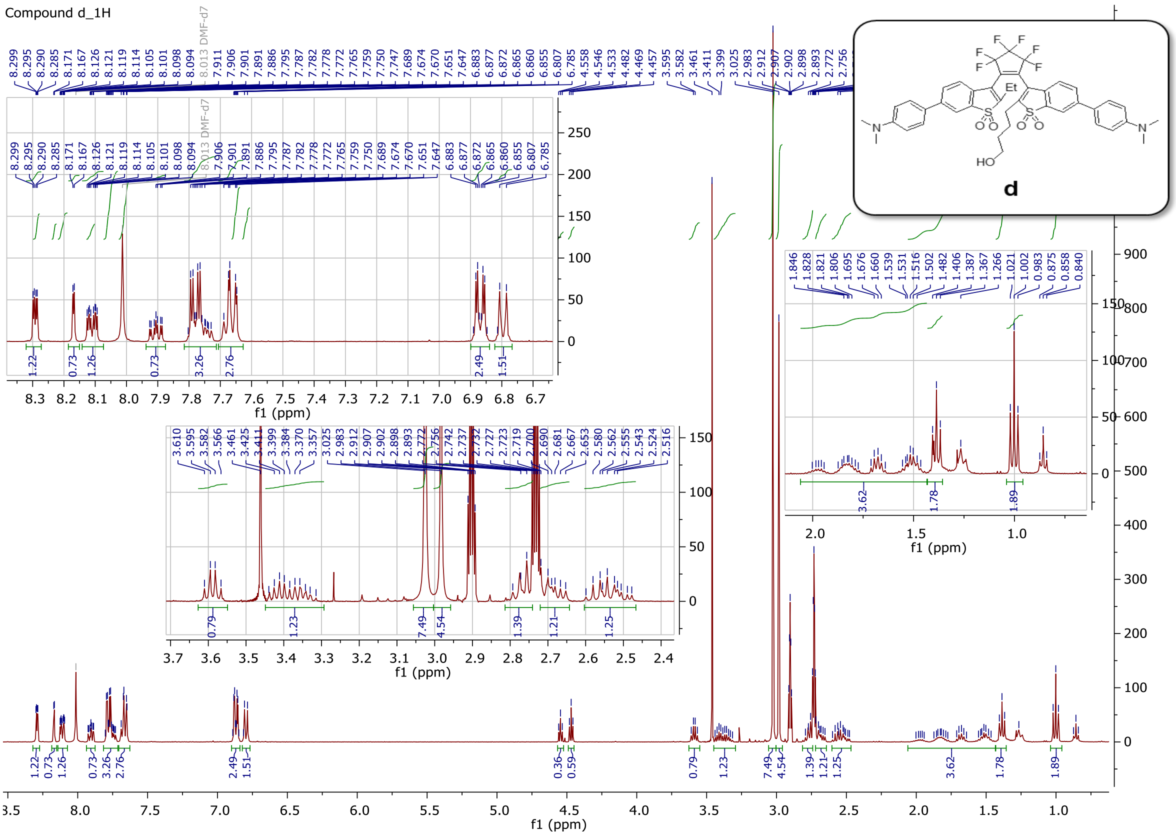

Figure S32. ^{1}H NMR spectrum (400 MHz, DMF-d_7) of compound **d**.

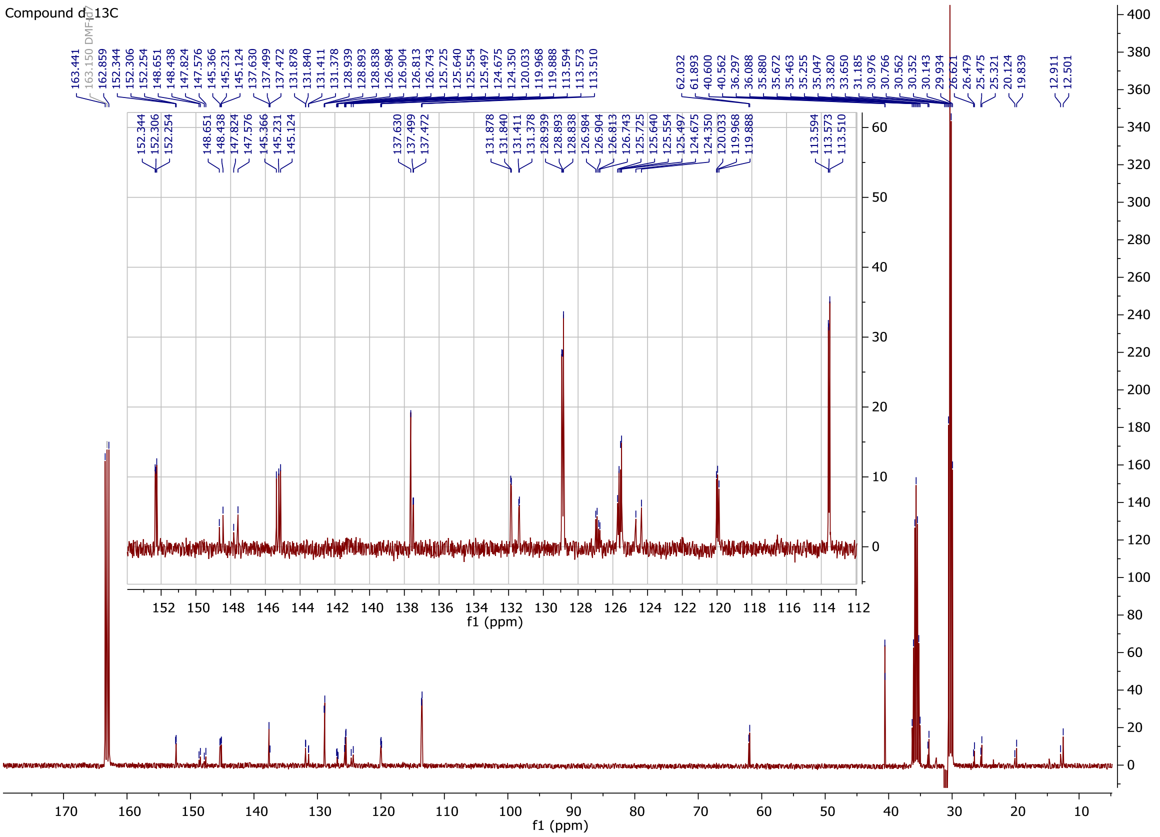

Figure S33. ^{13}C NMR spectrum (101 MHz, DMF-d_7) of compound **d**.

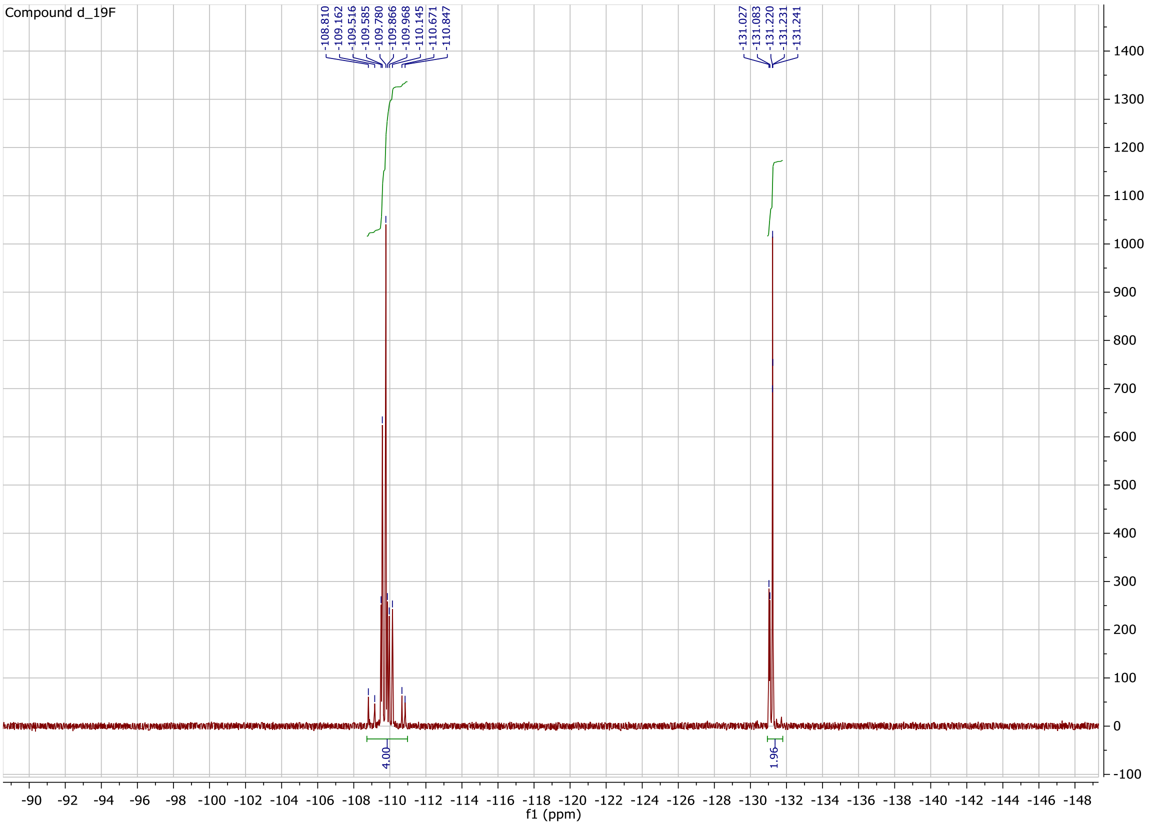

Figure S34. ^{19}F NMR spectrum (367 MHz, DMF-d_7) of compound **d**.

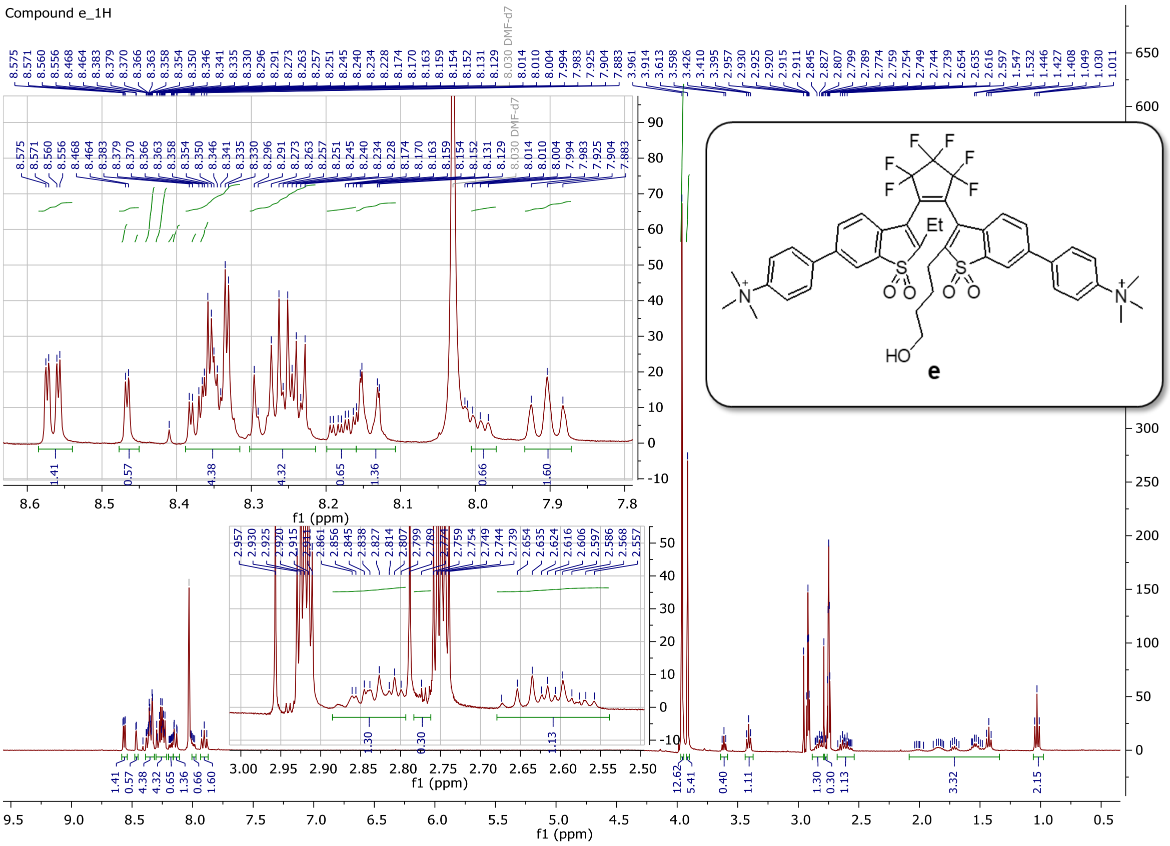

Figure S35. ^{1}H NMR spectrum (400 MHz, DMF-d_7) of compound **e**.

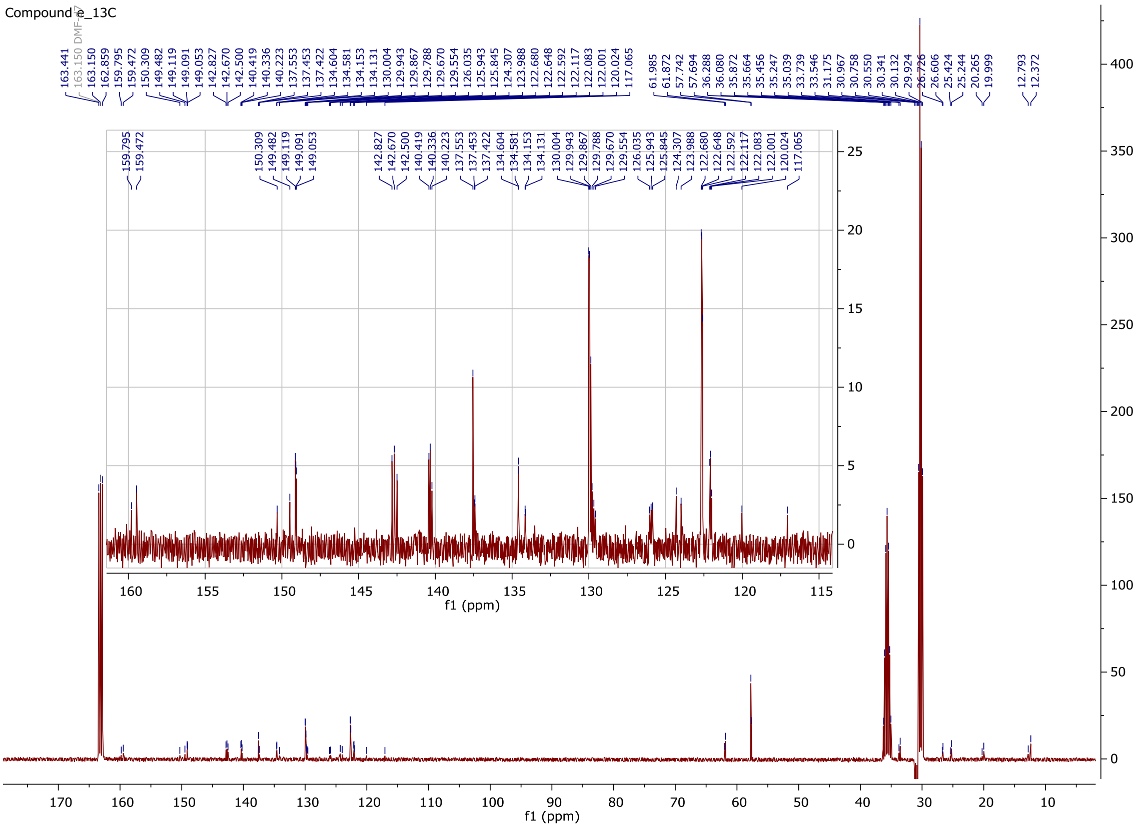

Figure S36. ^{13}C NMR spectrum (101 MHz, DMF-d_7) of compound **e**.

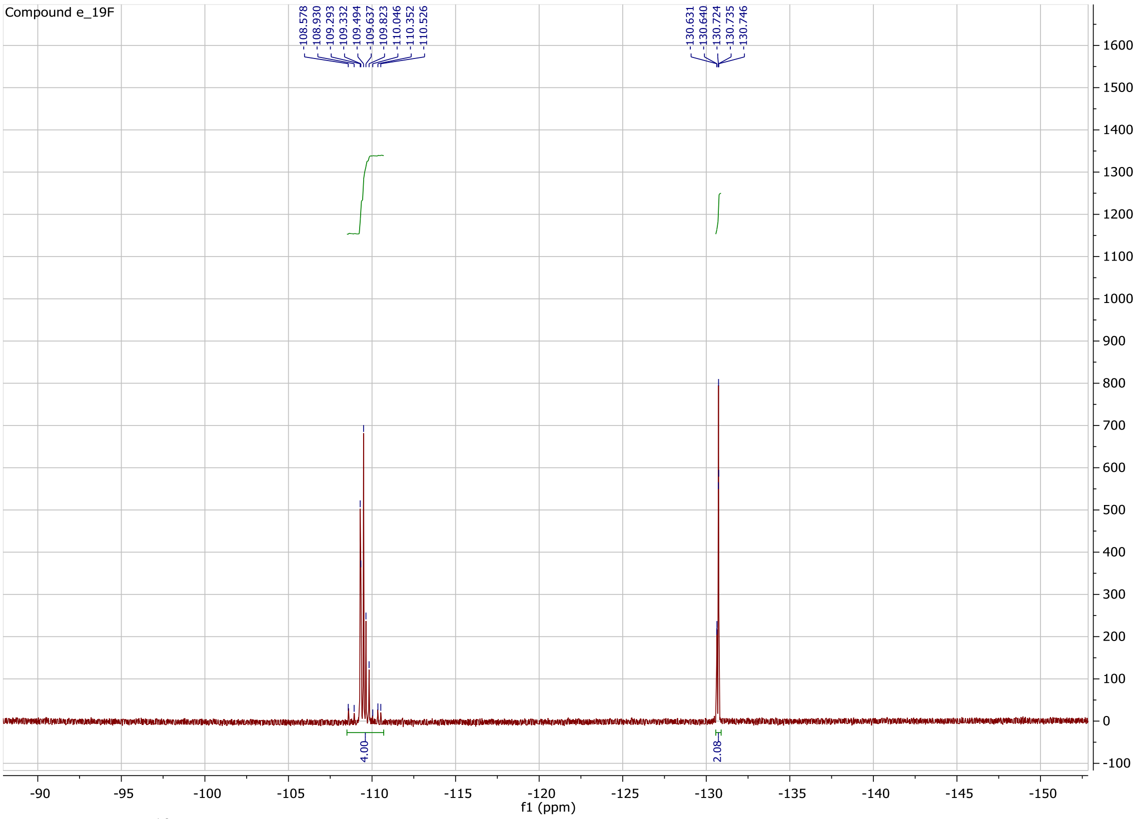

Figure S37. ^{19}F NMR spectrum (367 MHz, DMF-d_7) of compound **e**.

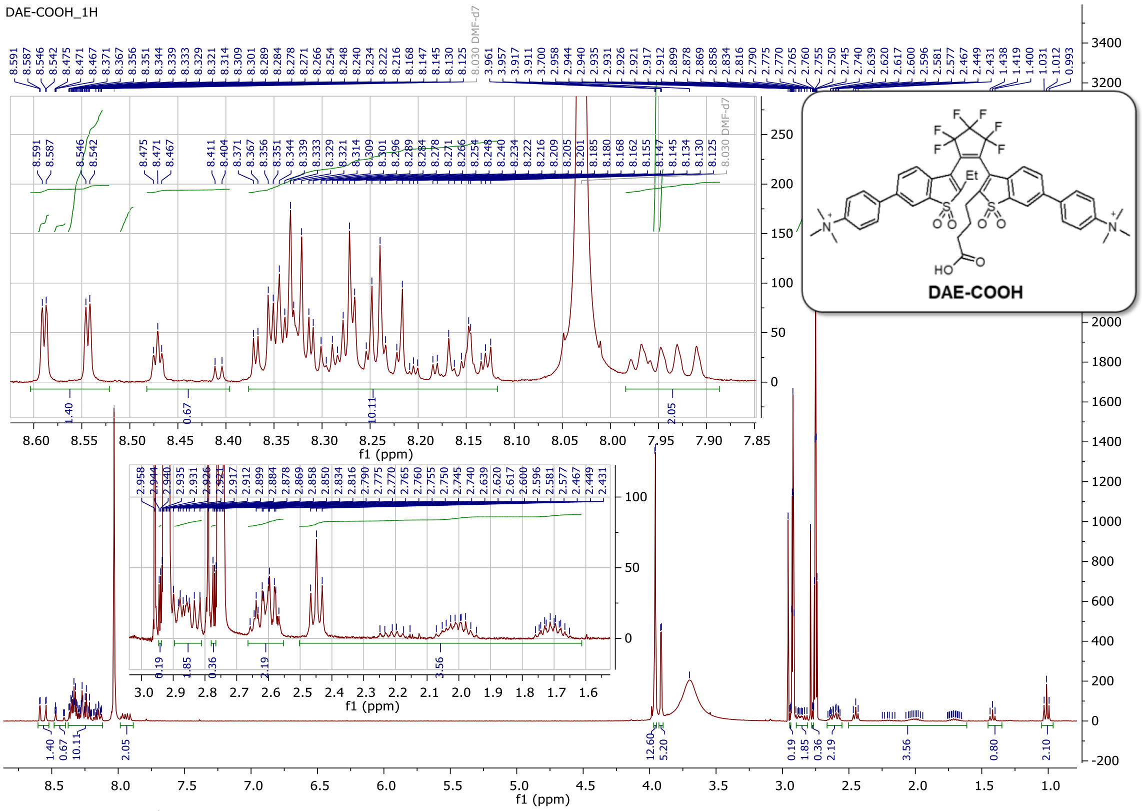

Figure S38. ^{1}H NMR spectrum (400 MHz, DMF-d_7) of compound **DAE-COOH**.

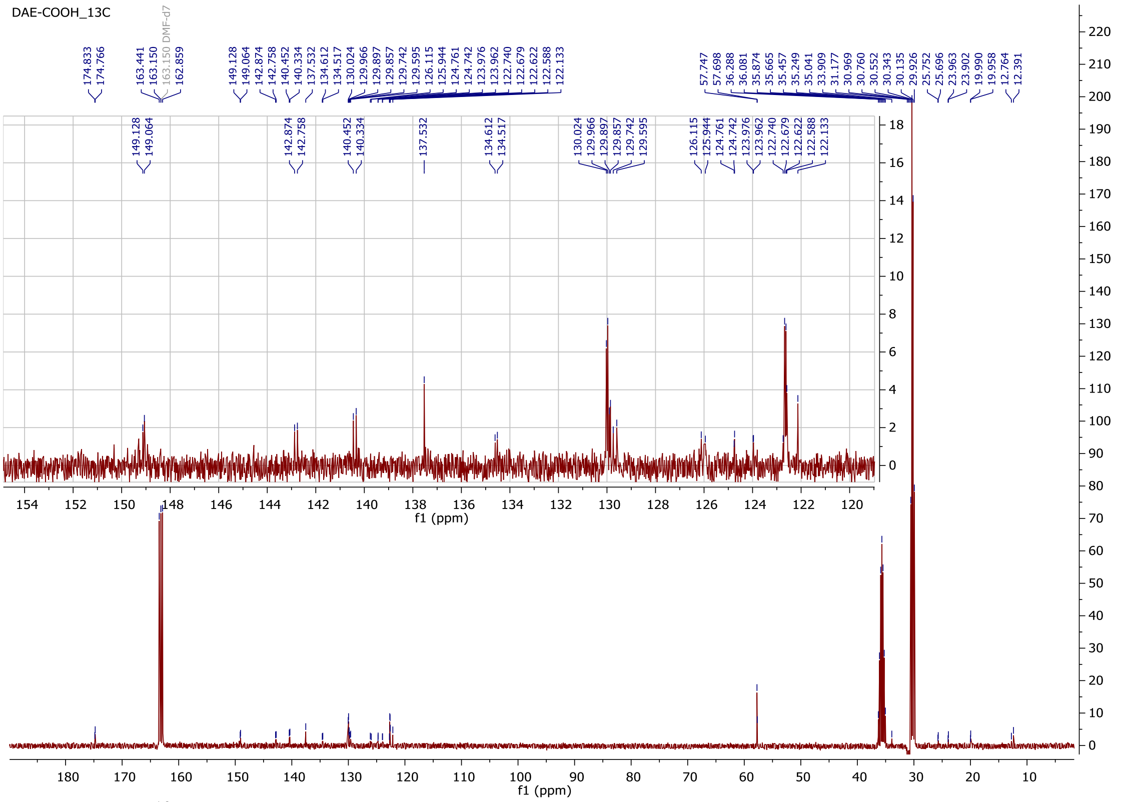

Figure S39. ^{13}C NMR spectrum (101 MHz, DMF-d_7) of compound **DAE-COOH**.

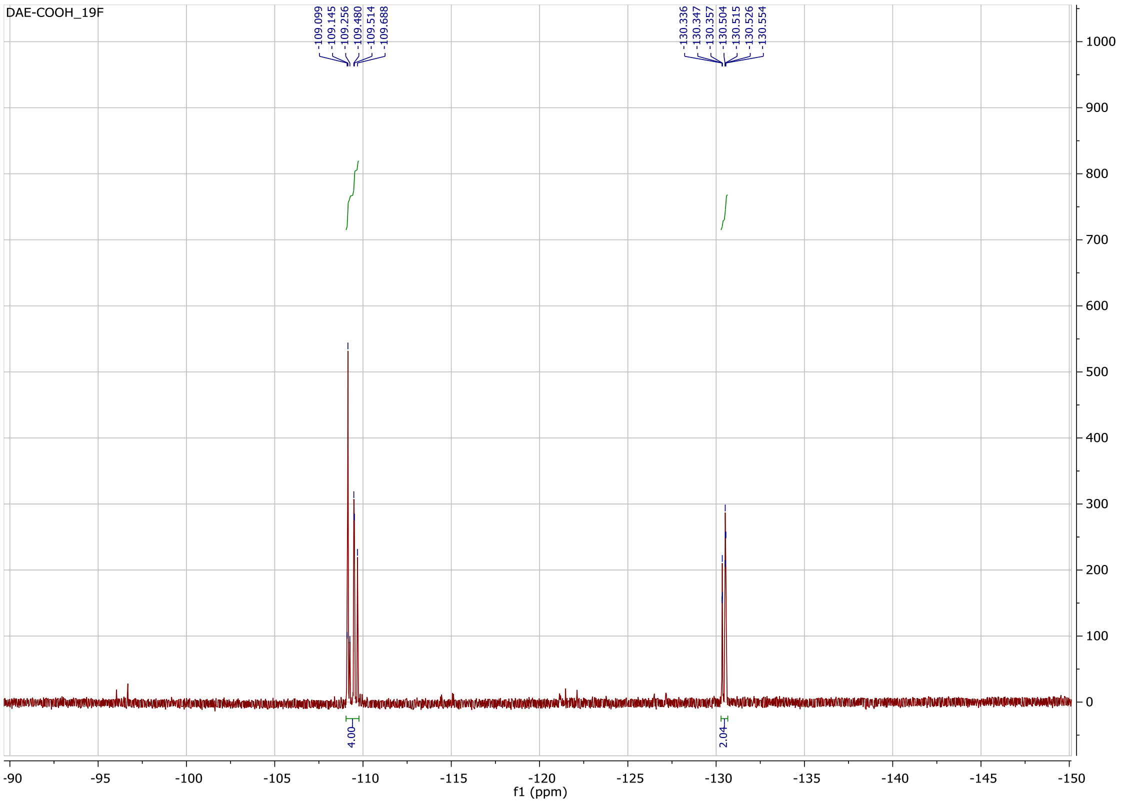

Figure S40. ^{19}F NMR spectrum (367 MHz, DMF-d_7) of compound **DAE-COOH**.

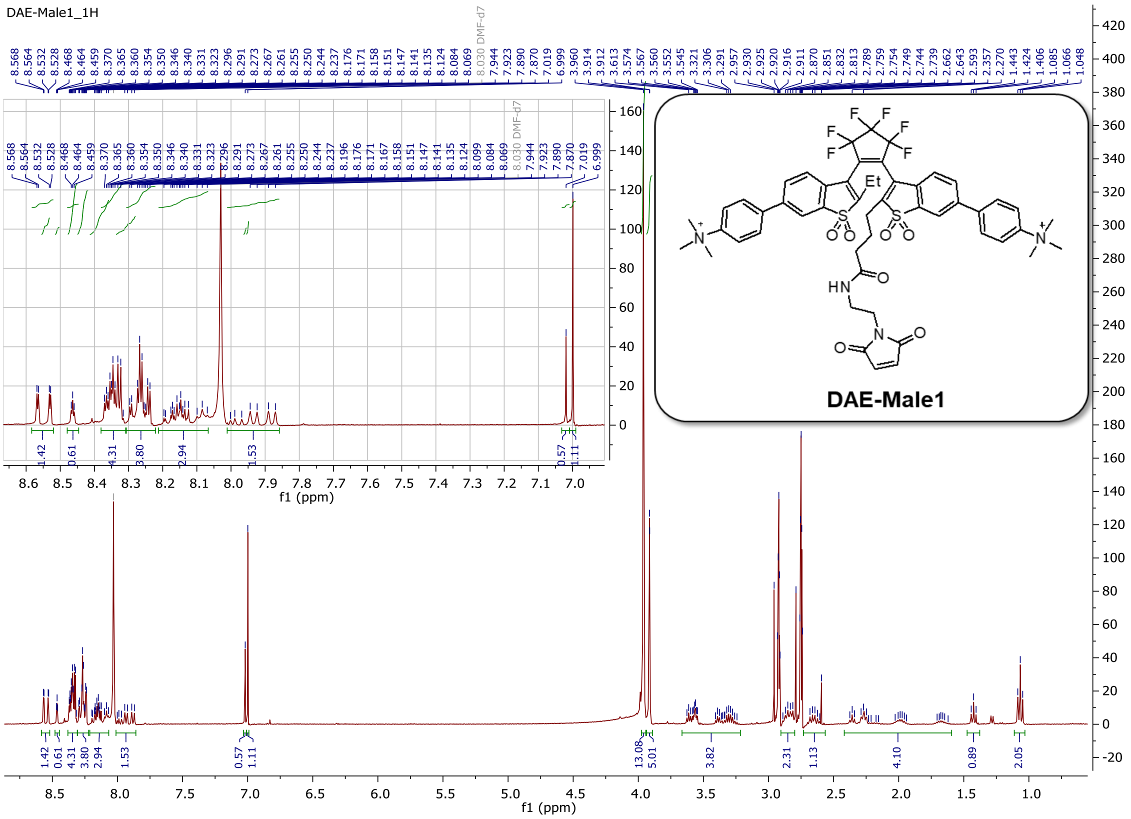

Figure S41. ^{1}H NMR spectrum (400 MHz, DMF-d_7) of compound **DAE-Male1**.

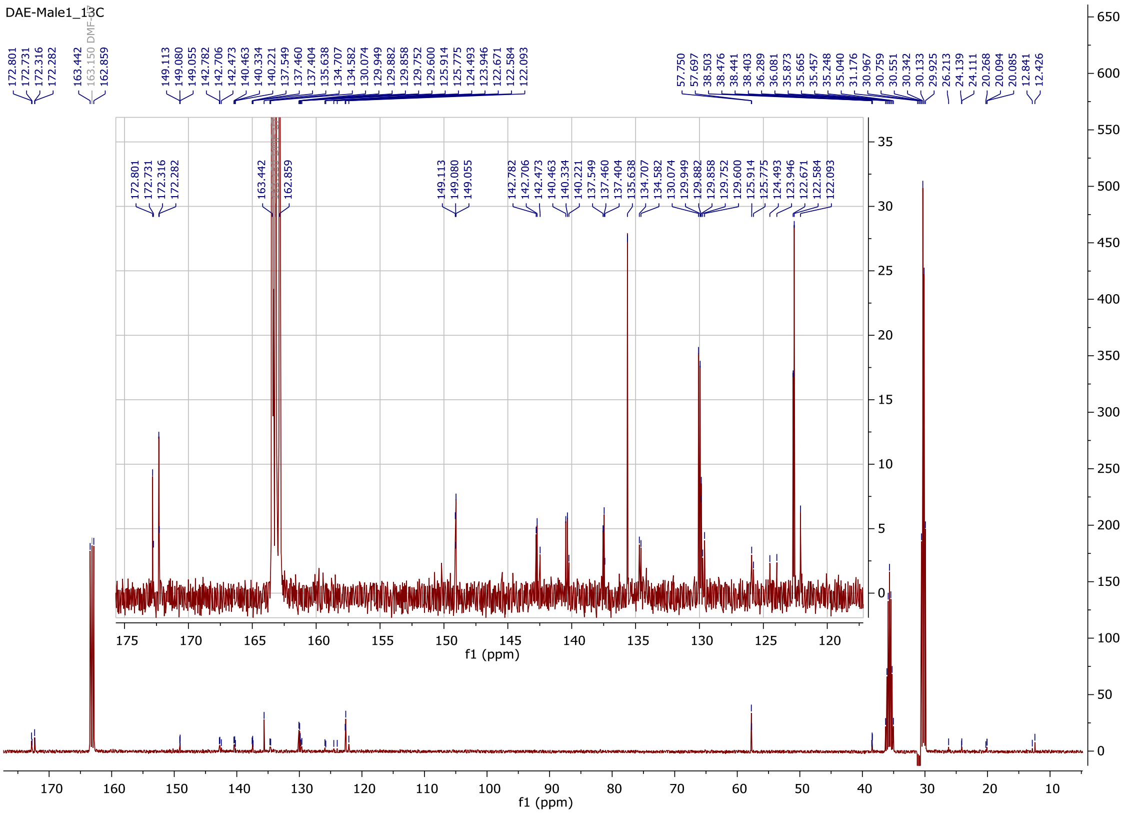

Figure S42. ^{13}C NMR spectrum (101 MHz, DMF-d_7) of compound **DAE-Male1**.

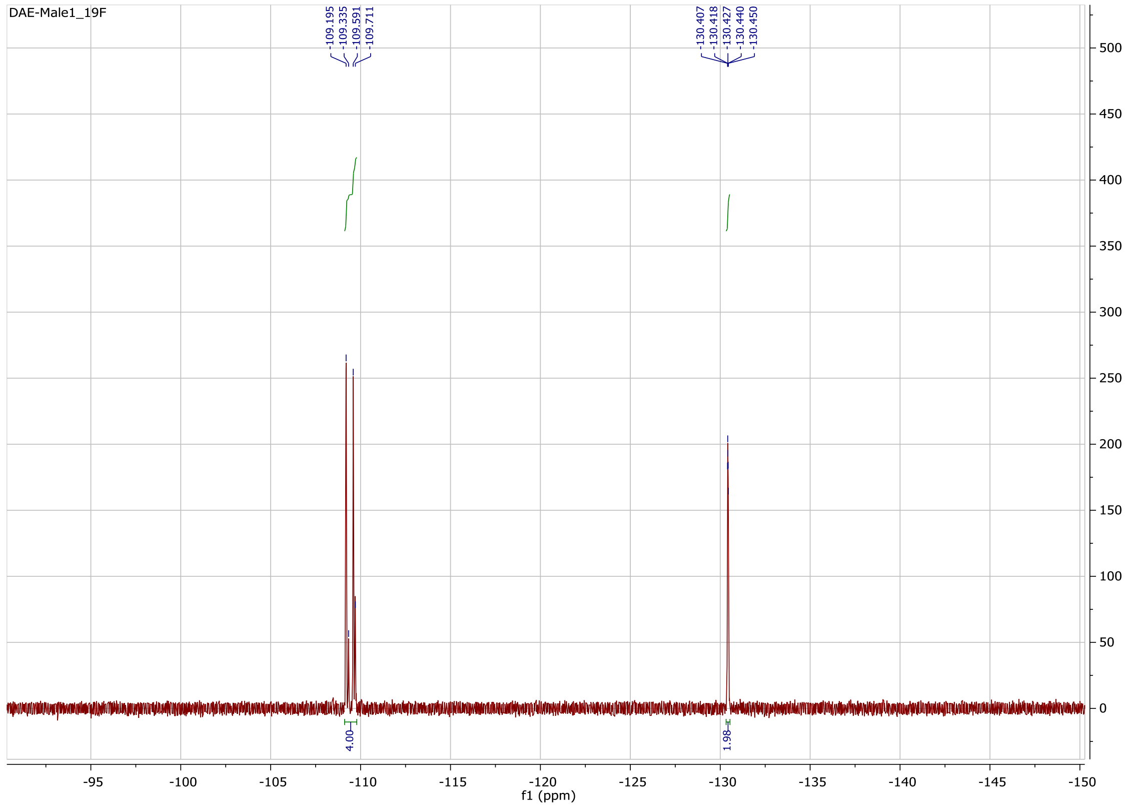

Figure S43. ^{19}F NMR spectrum (367 MHz, DMF-d_7) of compound **DAE-Male1**.

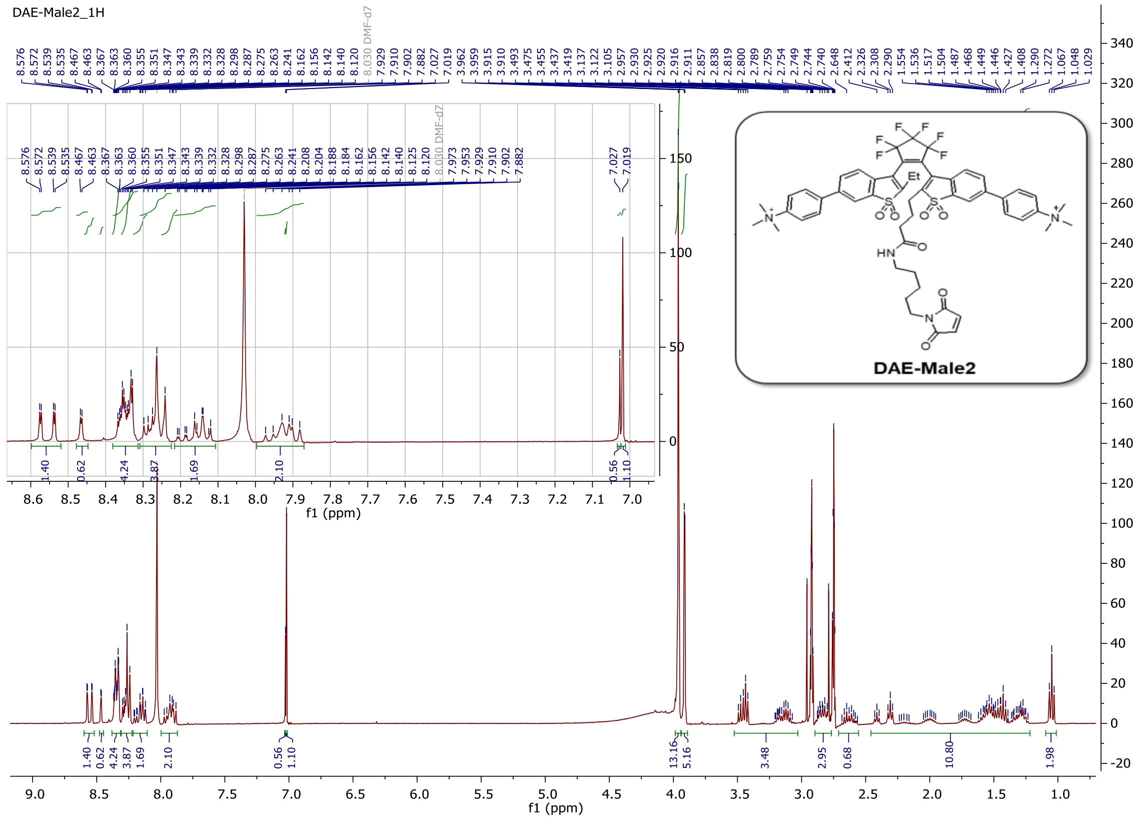

Figure S44. ^{1}H NMR spectrum (400 MHz, DMF-d_7) of compound **DAE-Male2**.

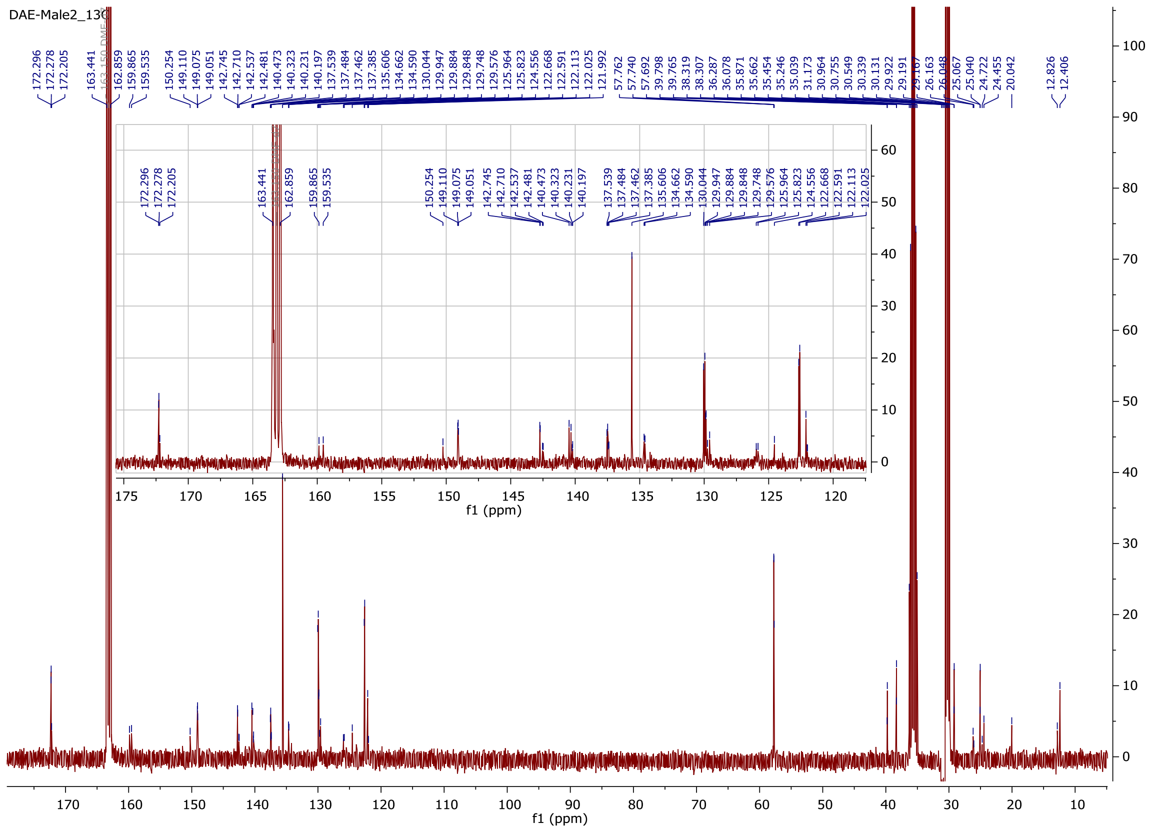

Figure S45. ^{13}C NMR spectrum (101 MHz, DMF-d_7) of compound **DAE-Male2**.

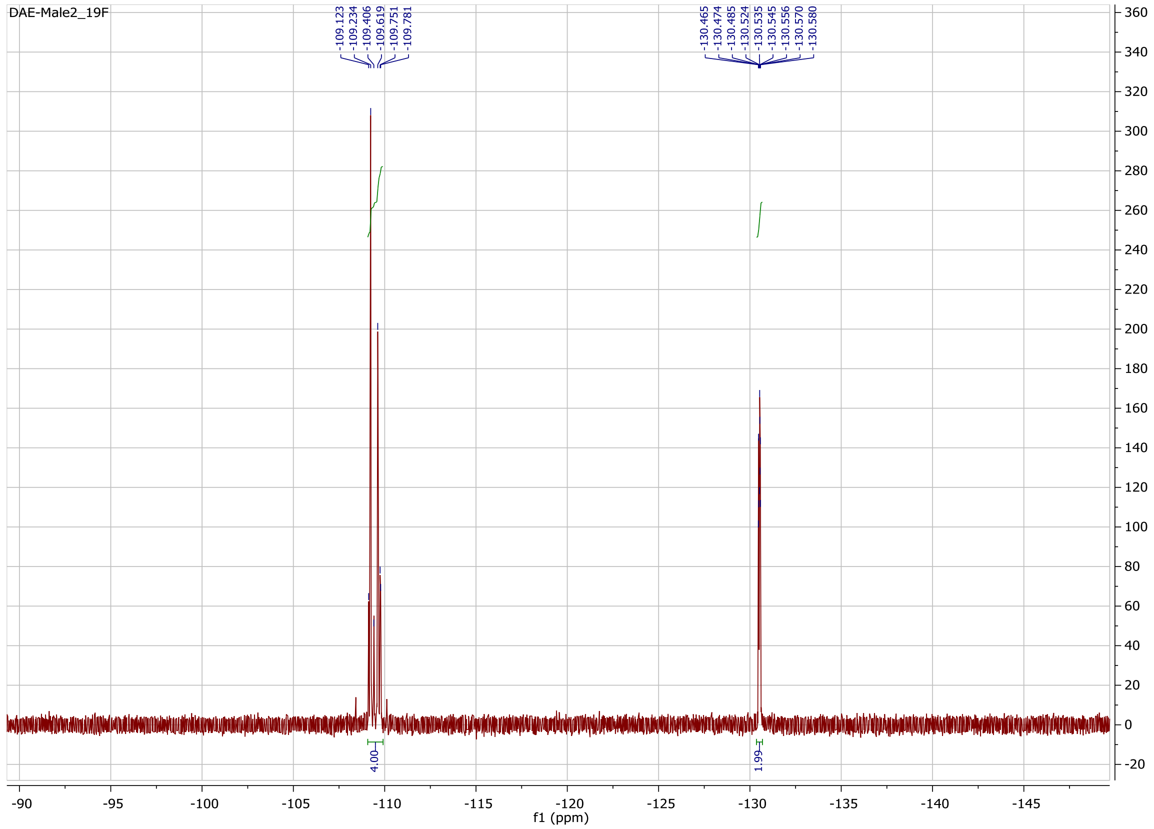

Figure S46. ^{19}F NMR spectrum (367 MHz, DMF-d_7) of compound **DAE-Male2**.

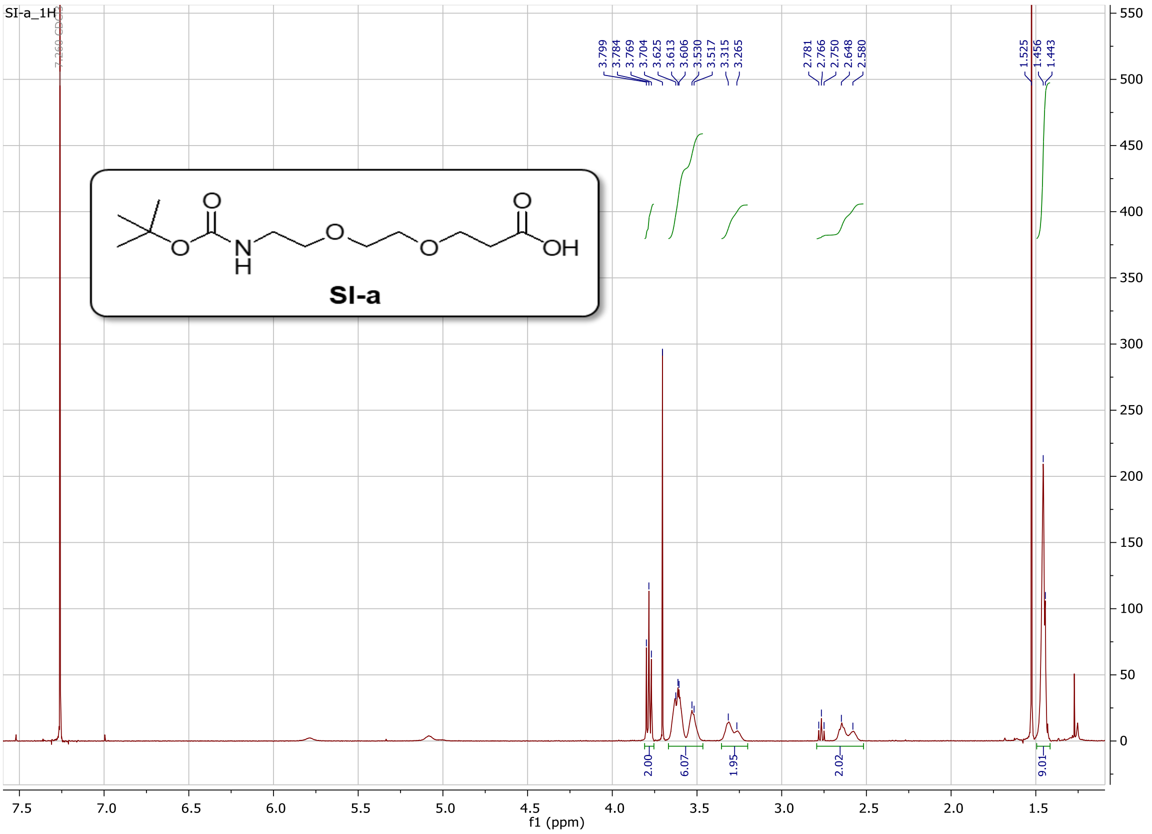

Figure S47. ^{1}H NMR spectrum (400 MHz, $CDCl_3$) of compound **SI-a**.

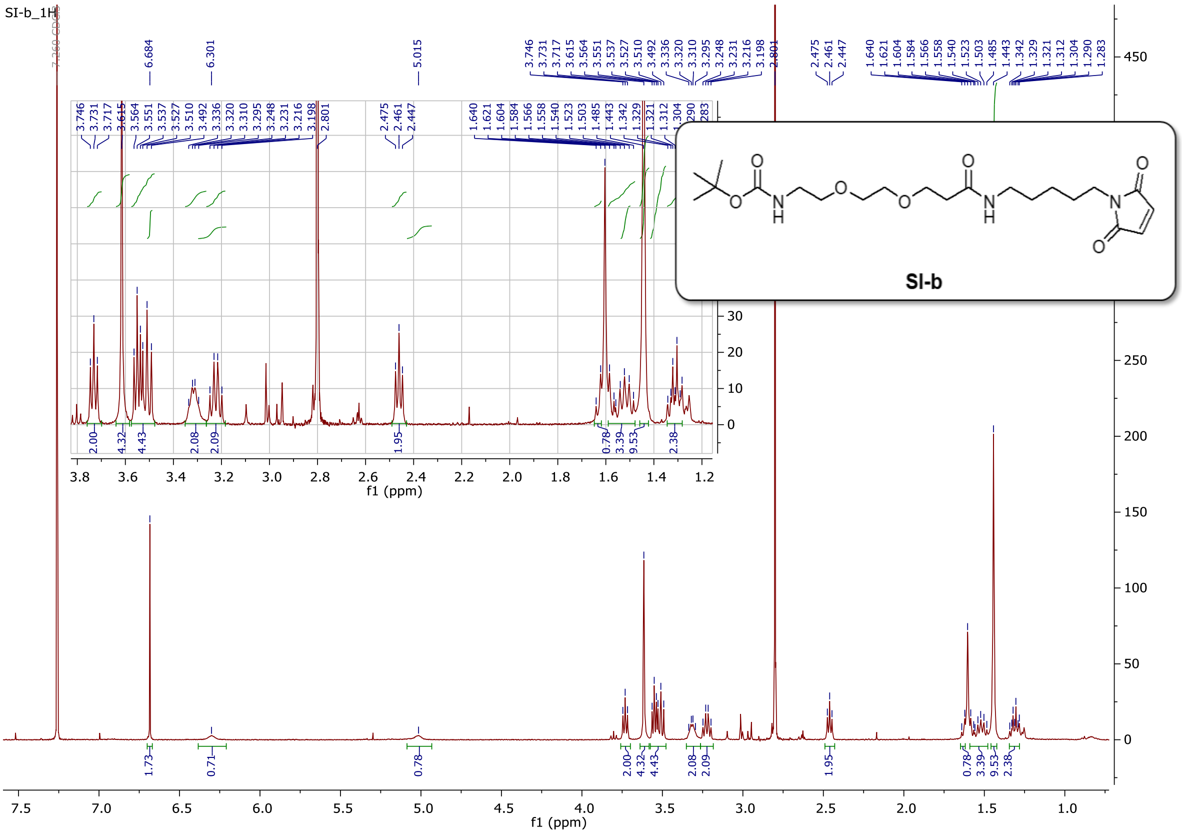

Figure S48. ^{1}H NMR spectrum (400 MHz, $CDCl_3$) of compound **SI-b**.

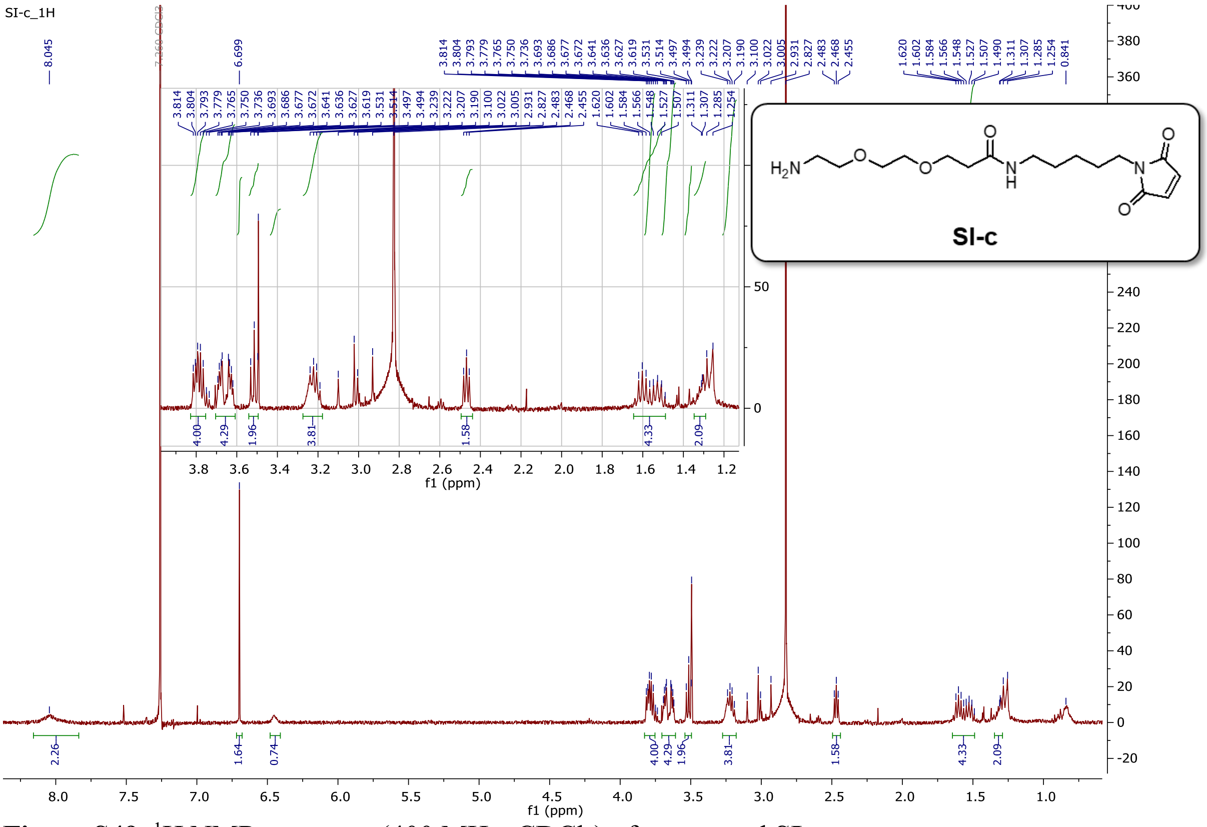

Figure S49. ^{1}H NMR spectrum (400 MHz, $CDCl_3$) of compound **SI-c**.

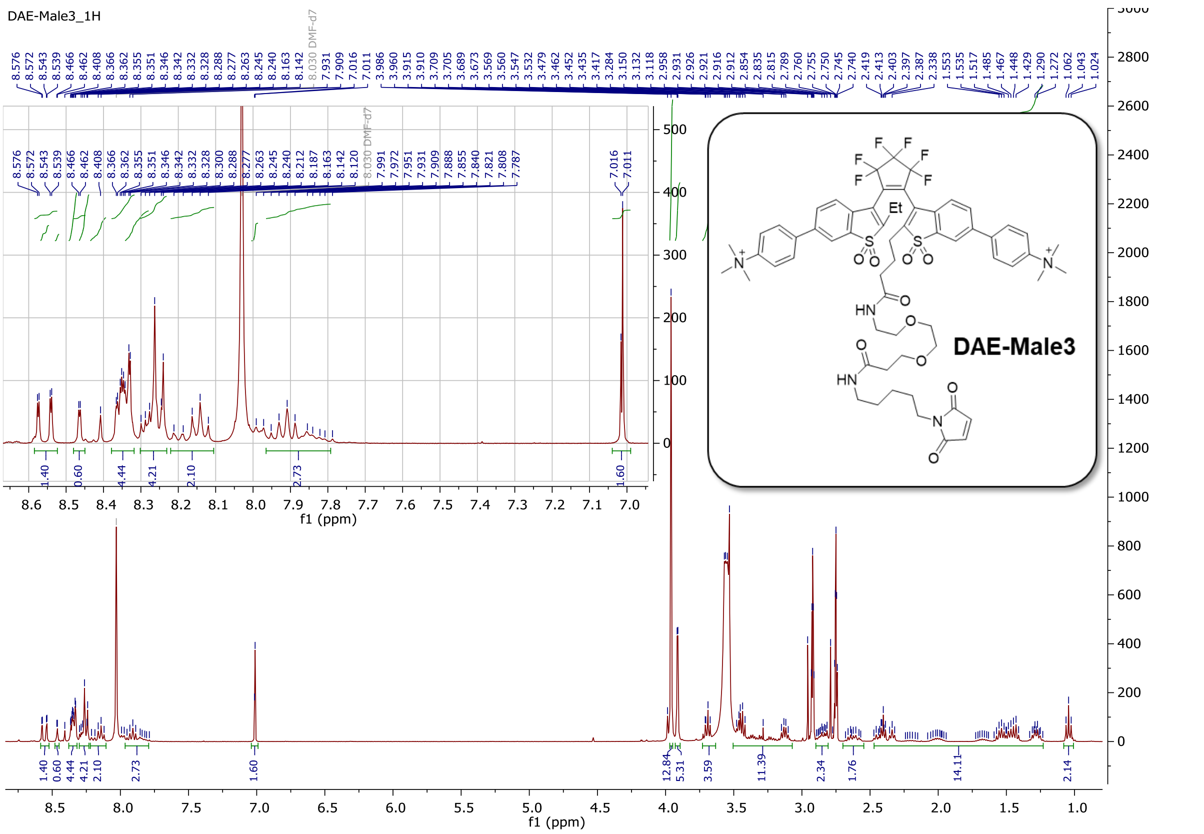

Figure S50. ^{1}H NMR spectrum (400 MHz, DMF-d_7) of compound **DAE-Male3**.

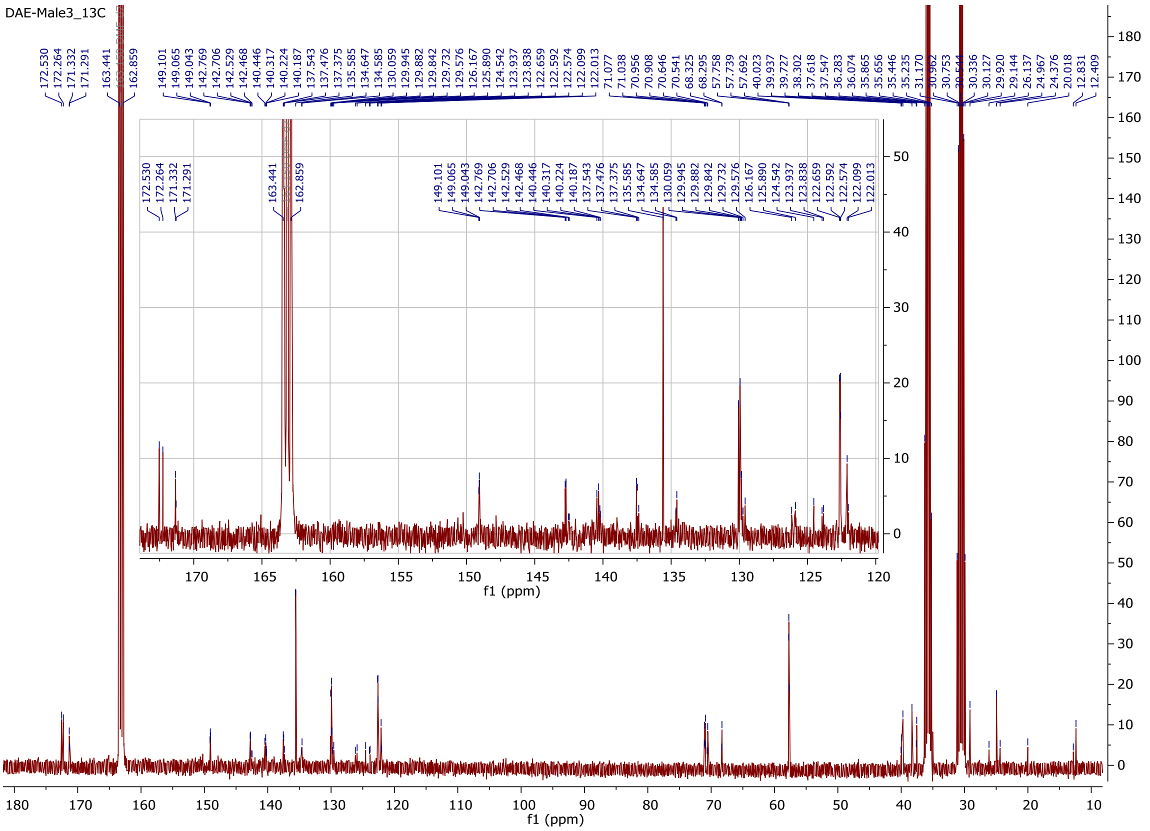

Figure S51. ^{13}C NMR spectrum (101 MHz, DMF-d_7) of compound **DAE-Male3**.

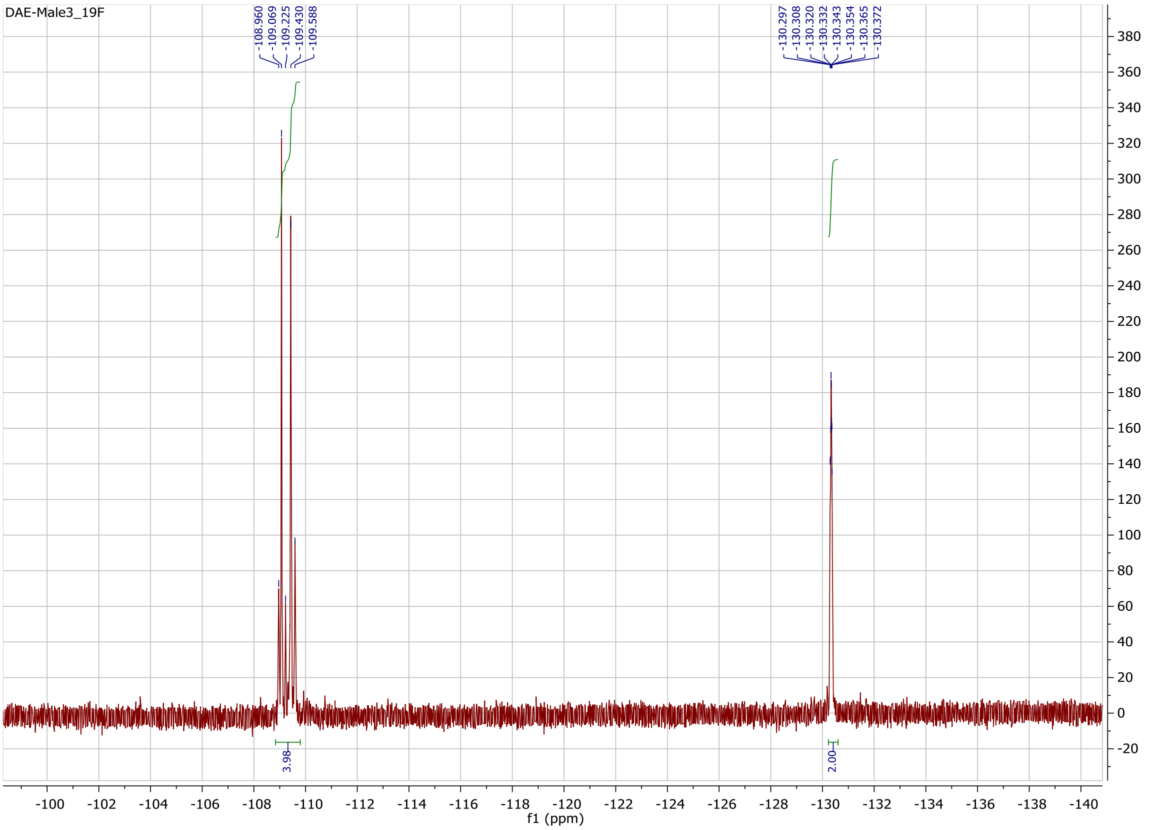

Figure S52. ^{19}F NMR spectrum (367 MHz, DMF-d_7) of compound **DAE-Male3**.

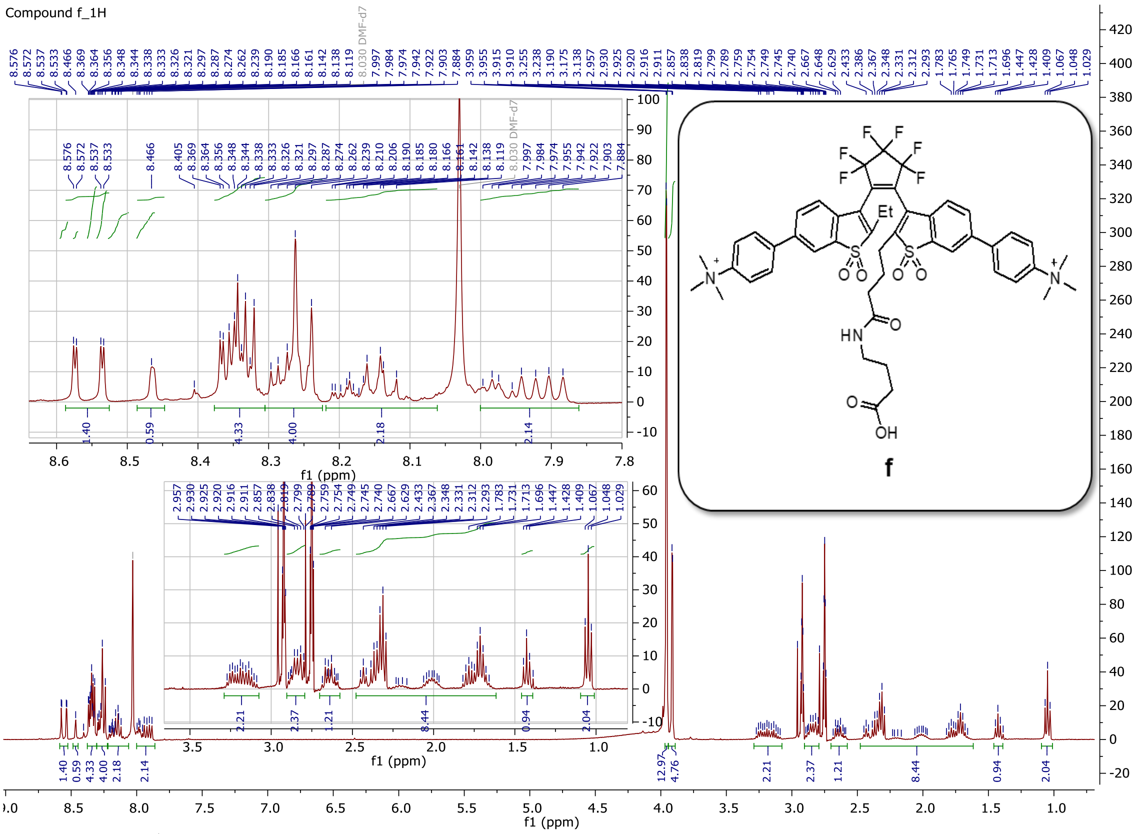

Figure S53. ^{1}H NMR spectrum (400 MHz, DMF-d_7) of compound **f**.

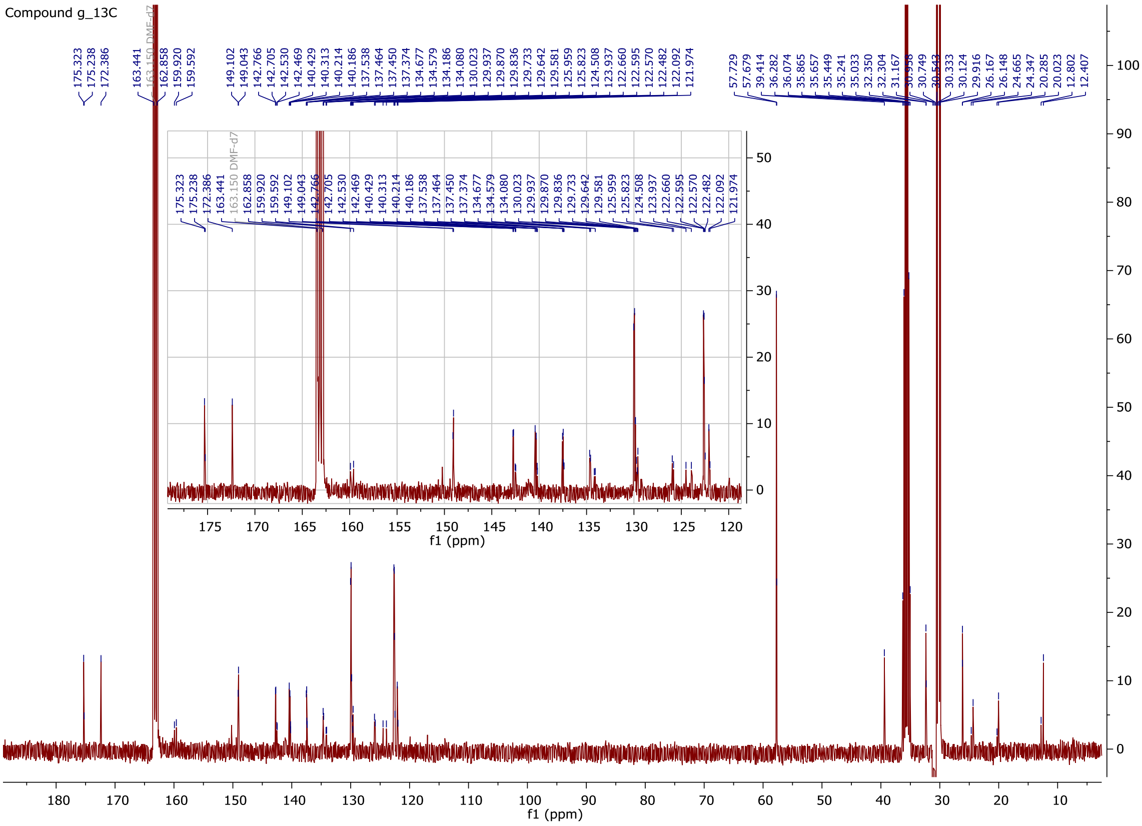

Figure S54. ^{13}C NMR spectrum (101 MHz, DMF-d_7) of compound **f**.

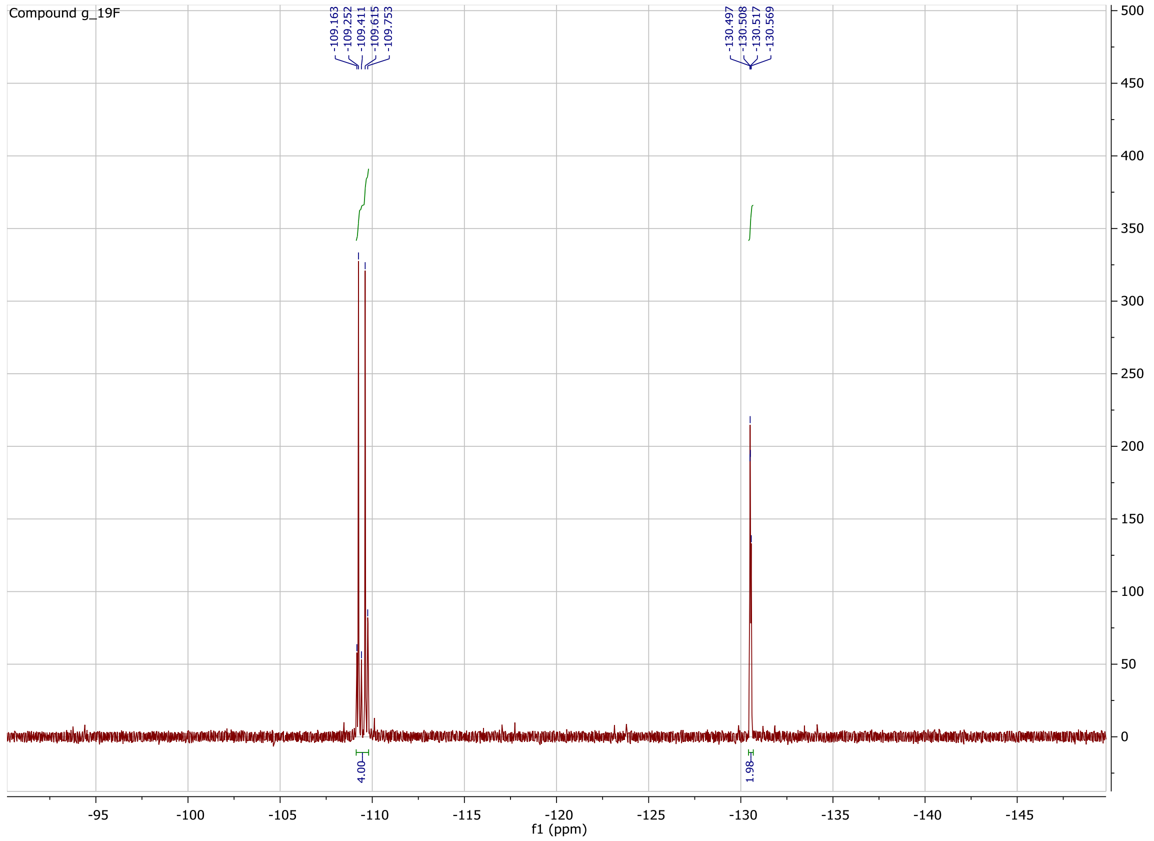

Figure S55. ^{19}F NMR spectrum (367 MHz, DMF-d_7) of compound **f**.

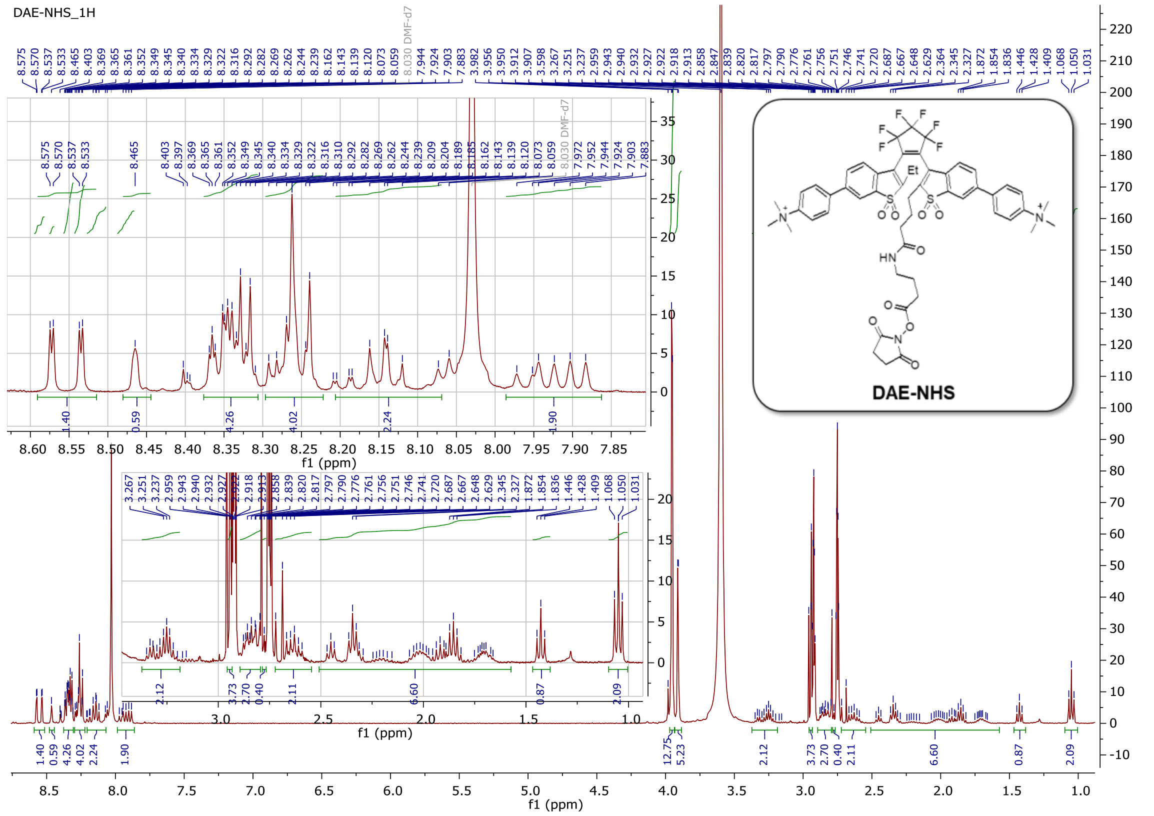

Figure S56. ^{1}H NMR spectrum (400 MHz, DMF-d_7) of compound **DAE-NHS**.

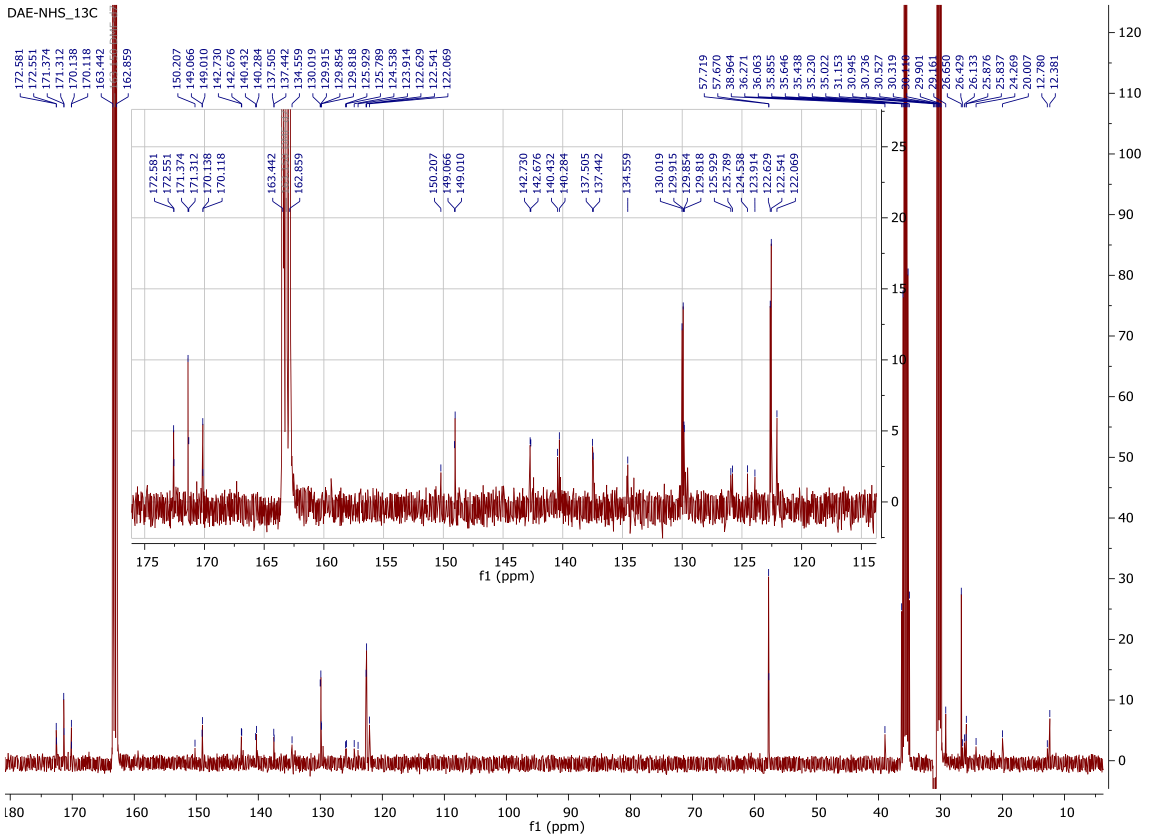

Figure S57. ^{13}C NMR spectrum (101 MHz, DMF-d_7) of compound **DAE-NHS**.

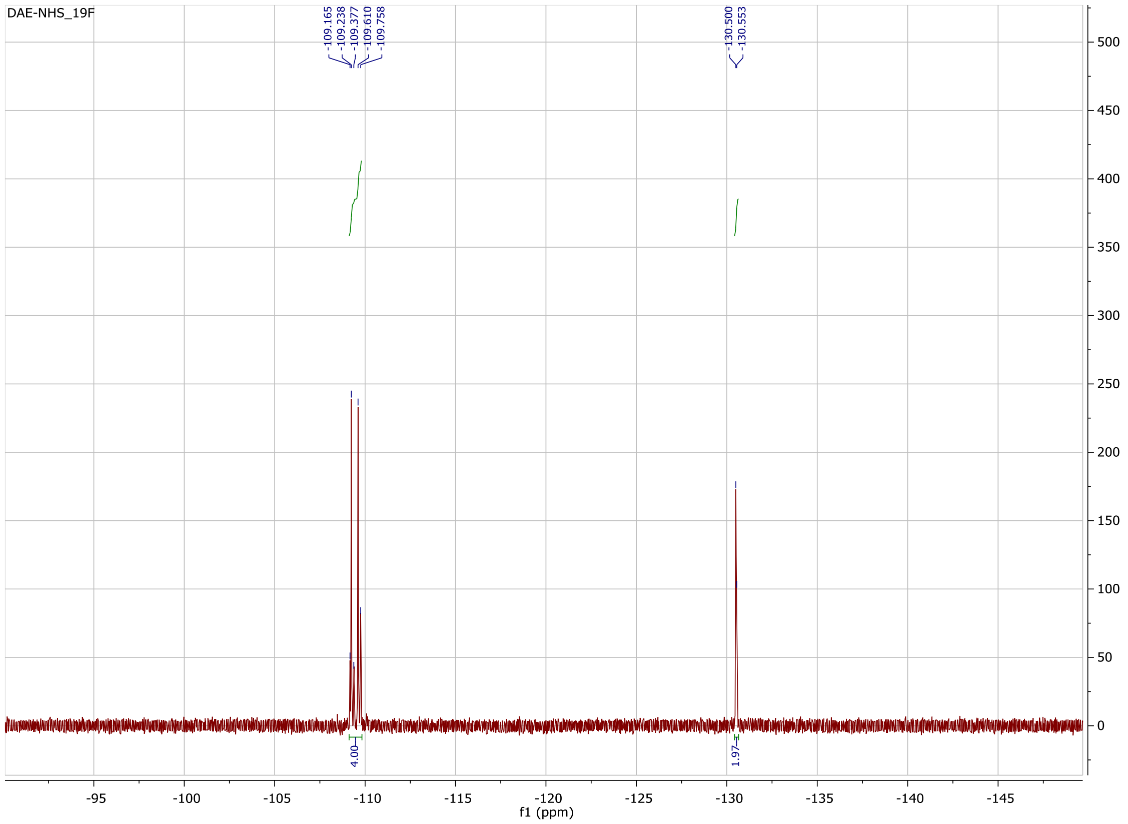

Figure S58. ^{19}F NMR spectrum (367 MHz, DMF-d_7) of compound **DAE-NHS**.

Supplementary References

[S1] Iwai, R.; Morimoto, M.; Irie, M., Turn-on mode fluorescent diarylethenes: effect of electron-donating and electron-withdrawing substituents on photoswitching performance. *Photochem. Photobiol. Sci.* **2020,** *19* (6), 783-789.

[S2] Uno, K.; Aktalay, A.; Bossi, M. L.; Irie, M.; Belov, V. N.; Hell, S. W., Turn-on mode diarylethenes for bioconjugation and fluorescence microscopy of cellular structures. *Proc. Natl. Acad. Sci. U. S. A.* **2021,** *118*, e2100165118.
